# First Structure–Activity-Relationship Study of Potent G2A Antagonists

**DOI:** 10.1021/acs.jmedchem.6c00079

**Published:** 2026-06-11

**Authors:** Victor Hernandez-Olmos, Jan Heering, Felix F. Lillich, Beatrice Marinescu, Sheila Nevermann, Johanna H. M. Ehrler, Dmytro S. Radchenko, Yurii S. Moroz, Astrid Kaiser, Andreas Krämer, Stefan Knapp, Manfred Schubert-Zsilavecz, Mohamad Wessam Alnouri, Stefan Offermanns, Dieter Steinhilber, Marco Sisignano, Ewgenij Proschak

**Affiliations:** † Fraunhofer Institute for Translational Medicine and Pharmacology ITMP, Theodor-Stern-Kai 7, 60596 Frankfurt am Main, Germany; ‡ Fraunhofer Cluster of Excellence Immune-Mediated Diseases CIMD, 60596 Frankfurt am Main, Germany; § Institute of Pharmaceutical Chemistry, 9173 Goethe University Frankfurt, Max-von-Laue-Str. 9, 60438 Frankfurt am Main, Germany; ∥ Institute of Clinical Pharmacology, Pharmazentrum Frankfurt/ZAFES, Goethe-University, D-60590 Frankfurt am Main, Germany; ⊥ 376198 Enamine Ltd. 78 Winston Churchill Street, Kyiv 02094, Ukraine; # Chemspace LLC, 85 Winston Churchill Street, Suite 1, Kyiv 02094, Ukraine; ∇ National Taras Shevchenko University of Kyiv, 60 Volodymyrska Street, Kyiv 01601, Ukraine; ○ Department of Pharmacology, 28258 Max Planck Institute for Heart and Lung Research, 61231 Bad Nauheim, Germany; ◆ Center for Molecular Medicine, Goethe University Frankfurt, 60590 Frankfurt, Germany

## Abstract

G2A inhibition has recently been proposed as a novel therapeutic approach to treat oxaliplatin-induced neuropathic pain (OINP) and breast cancer. However, very few G2A antagonists are known to date. In this study, we report the discovery of a novel series of G2A antagonists developed within our research group, along with the first comprehensive structure–activity relationship (SAR) investigation for this class of compounds. Utilizing a rational design approach, we systematically explored the effects of structural modifications on G2A receptor binding and functional activity. The SAR study identified key molecular features critical for potent G2A inhibition. Two of the newly discovered compounds exhibited submicromolar activity and acceptable selectivity profile among GPCRs.

## Graphical abstract

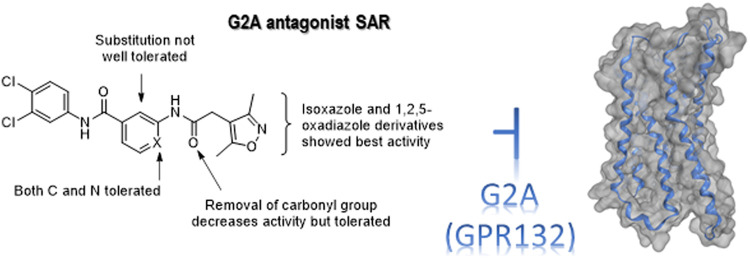

## Introduction

The G protein coupled receptor 132 (GPR132 or G2A), a member of the G protein-coupled receptor (GPCR) family, has been implicated in various physiological processes, including immune regulation, inflammation, and pain modulation.^1^ In a recent publication, we have described potent and selective G2A agonists.^2^ However, G2A antagonists have also emerged as potential therapeutic agents for a wide range of diseases and conditions. G2A shows the strongest expression in leukocytes, neutrophils, and macrophages,^3–5^ and is also found in peripheral sensory neurons that coexpress the transient receptor potential vanilloid 1 channel (TRPV1).^6^ In this context G2A has been shown to play a key role in the development and persistence of oxaliplatin-induced neuropathic pain (OINP) through activation of PKC (protein kinase C) and subsequent sensitization of TRPV1. Therefore, it is suggested that OINP can indeed be reduced by inhibition of G2A.^7^ Furthermore, inhibition of G2A has also been proposed as a novel treatment against breast cancer.^8,9^ Additionally, G2A deficient mice have been reported to be able to modulate severity of invasive pulmonary aspergillosis (IPA).^10^

Lipids are considered to activate G2A, in particular, lysophosphatidylcholine (LPC) was first described as an agonist.^5^ However, this paper was later retracted^11^ and several other studies confirmed its lack of agonist effects.^12^ Another study concluded that LPC is only a weak antagonist of G2A, with an IC_50_ greater than 10 μM.^4^ Regarding synthetic inhibitors, one work by Brown et al. showed preliminary data identifying compounds SB-583355 (Figure 1A) and GSK1820795A (Figure 1B), a telmisartan analog, as G2A inhibitors,^12^ while a very recent work linked the potent G2A antagonist NOX-6–18 (Figure 1C) as a potential treatment for diabetes.^13^

**Figure 1:**
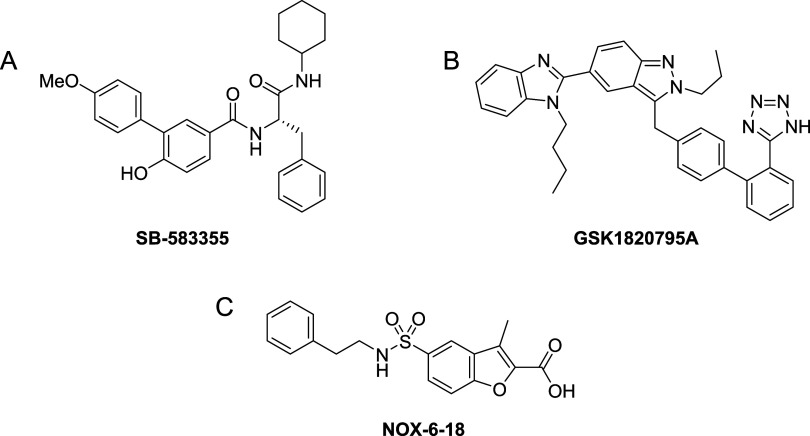
Structures of G2A antagonists SB-583355 (A) GSK1820795A (B) and NOX-6–18 (C).

Overall, inhibiting G2A provides promising new avenues for therapeutic development. In this study, we report the discovery and structure–activity relationships (SARs) of novel, potent, and selective G2A antagonists. Some of the newly prepared compounds presented submicromolar activity and good selectivity profile among other GPCRs. Compound **65** showed also a promising PK making it an interesting chemical tool for *in vivo* G2A studies.

## Results and Discussion

### High Throughput Screening for Identification of G2A Agonists

In the search for novel G2A antagonists, approximately 25,000 compounds from the Enamine library were screened in an unbiased high-throughput screening (HTS) campaign. The compounds were evaluated in a functional cell-based assay that measured inositol monophosphate (IP-1) accumulation as a readout of G2A activation. The utilized CHO-K1 cell line harbors expression cassettes for G2A and for GNA11 (G α subunit 11) both under the control of the constitutively active EF1α (elongation factor 1α) promoter. In order to test for antagonists, the receptor was activated with 12.5 μM of reference agonist (±) 9-HODE (EC_50_ = 7.5 μM) in the presence of 50 μM of test compound or DMSO as control. Notably, while reference antagonist SB-583355 (Figure 1) was confirmed as G2A antagonist in this assay, NOX-6–18 failed to inhibit the activation of G2A by (±) 9-HODE (Table 1 and Supporting Information, Figure S12). Compounds were selected evenly from all subsets of the library, with plates enriched in carboxylic acid-containing compounds prioritized. As a result, approximately 3,000 carboxylic acids were included among the 25,000 screened compounds. The most promising hit, compound **1** (Figure 2), was chosen for further optimization based on its potency (IC_50_ = 1.42 μM) and synthetic accessibility.

**Figure 2:**
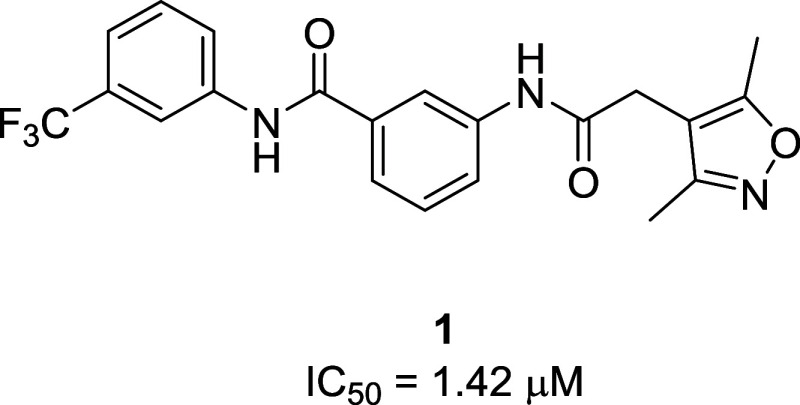
Compound **1**, the most promising candidate for the HTS campaign.

**Table 1:**
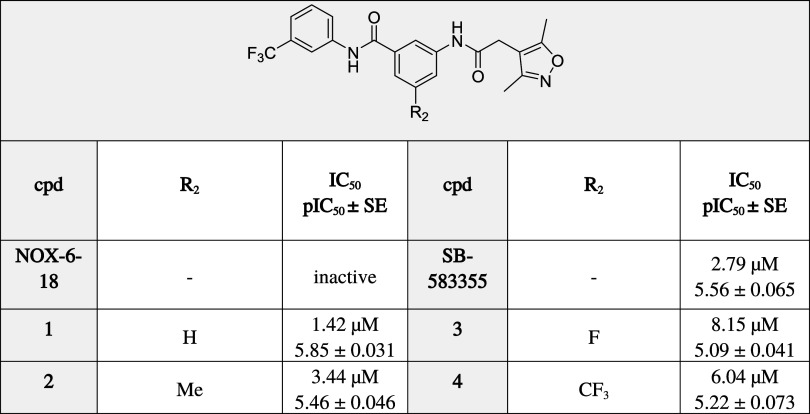
Introduction of Substitution in the Central Aromatic Ring (**2–4**)

### Chemistry

To investigate the SARs of the selected scaffold, the synthetic work was divided in two parts. First, a set of **27** compounds (**2–28**) was prepared to examine the role of substitution on the western aromatic ring, the replacement of the isoxazole for other heterocycles, and the introduction of substituents on the central aromatic ring. These compounds was synthesized in a single amide-coupling step from different carboxylic acids and amines as starting materials as shown in Scheme 1, and their final structures and activity results are presented in Tables 1–3.

**Scheme 1:**
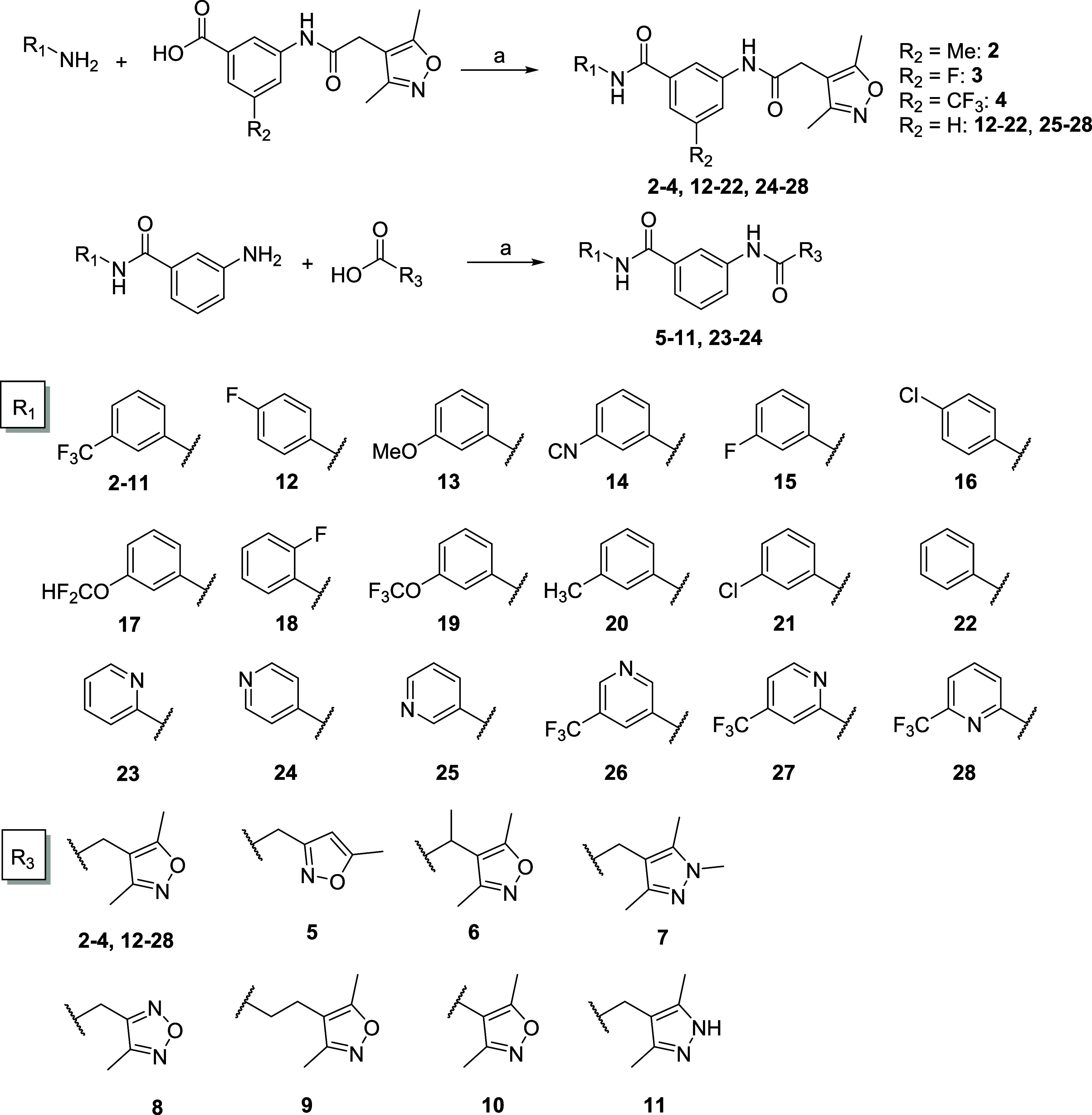
Synthetic Procedure Used for the Synthesis of Compounds **1–28** ^a^

a Conditions. (a) CDI, DMF, rt, 12-78%.

Following these initial set of derivatives, a more comprehensive SAR study was planned to further evaluate the most effective modifications for each part of the scaffold. We started by focusing on the western aromatic ring to generate disubstituted compounds. Most of the new compounds were prepared following the synthetic procedure depicted in Scheme 2. First, compound **29** was prepared from commercially available methyl 3-aminobenzoate and 2-(3,5-dimethylisoxazol-4-yl)­acetic acid under amide coupling conditions with HBTU and EDCl as amide coupling reagents and 4-methylmorpholine as base. Next, hydrolysis of the ester group under basic conditions provided carboxylic acid **30**. Then, the final products (**31–36**) were prepared from **30** and the corresponding aniline in the same conditions as used in the synthesis of intermediate compound **29**. Due to the electron-withdrawing nature of some of the aniline substituents used, low reactivity was observed leading to low yields or no reaction as in the case of compounds **32** and **33**. In these cases alternative amide coupling conditions (Scheme 2, procedures d and e) were used to obtain the final compounds in moderate yield.

**Scheme 2:**
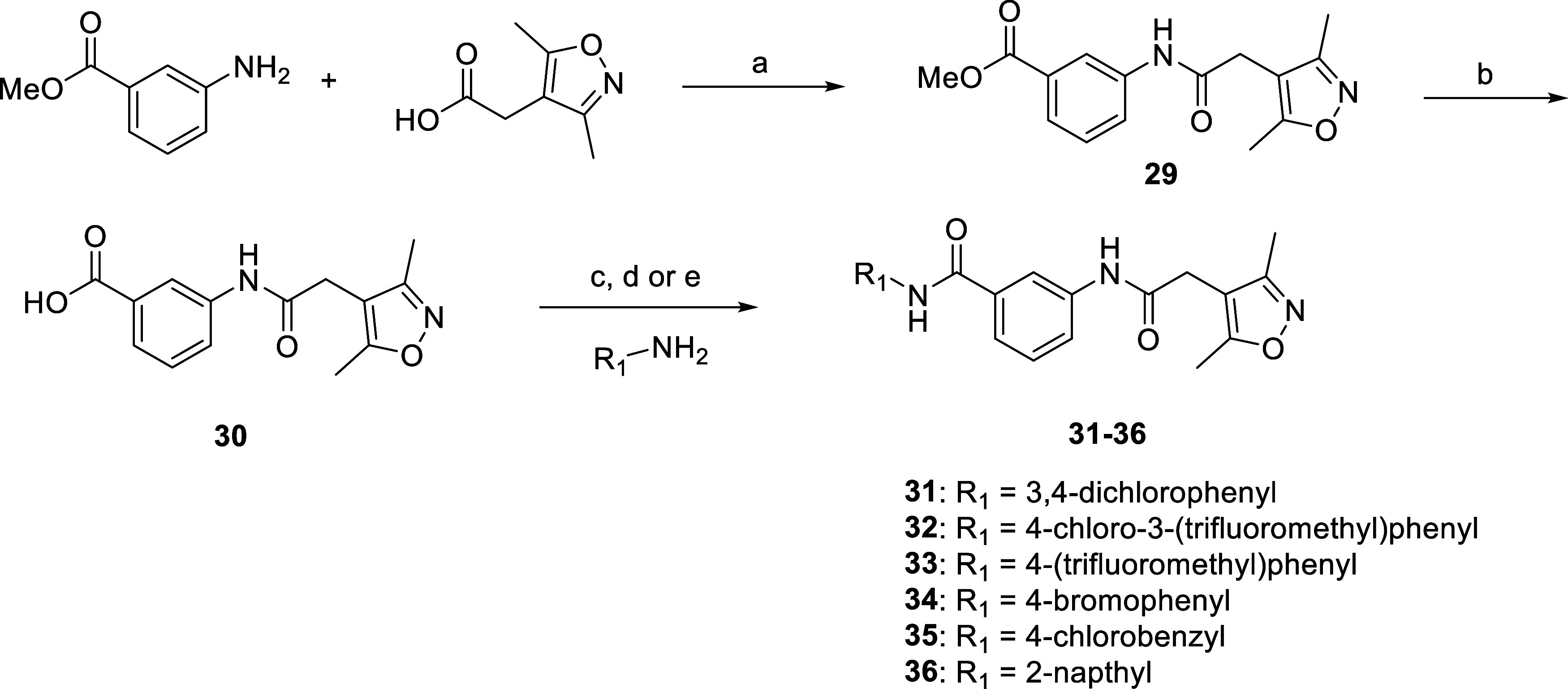
Synthetic Procedure Used for the Synthesis of Compounds **31**–3**6** ^a^

a Conditions. (a) HBTU, EDCl, 4-methylmorpholine, DMF, 91% (b) LiOH/H_2_O, THF/MeOH, 60 °C, 82% (c) HBTU, EDCl, 4-methylmorpholine, DMF for **31**, **34-36**, 11-81% (d) 1. SOCl_2_, DCM 2. DIPEA, DCM for **32**, 32% (e) DCC, DCM for **33**, 32%.

In order to overcome the challenging amide formation with other less reactive anilines, alternative synthetic routes were developed (Schemes 3 and 4). In the first approach (Scheme 3), the unreactive anilines were coupled in the first step of the synthesis route to the highly reactive 3-nitrobenzoyl chloride to obtain intermediates **37–39** in moderate to good yields. Subsequently, tin­(II) chloride was used to reduce the nitro group under acidic conditions and the final compounds **43–45** were obtained by reaction of anilines **40–42** with 2-(3,5-dimethylisoxazol-4-yl)­acetic acid.

**Scheme 3:**
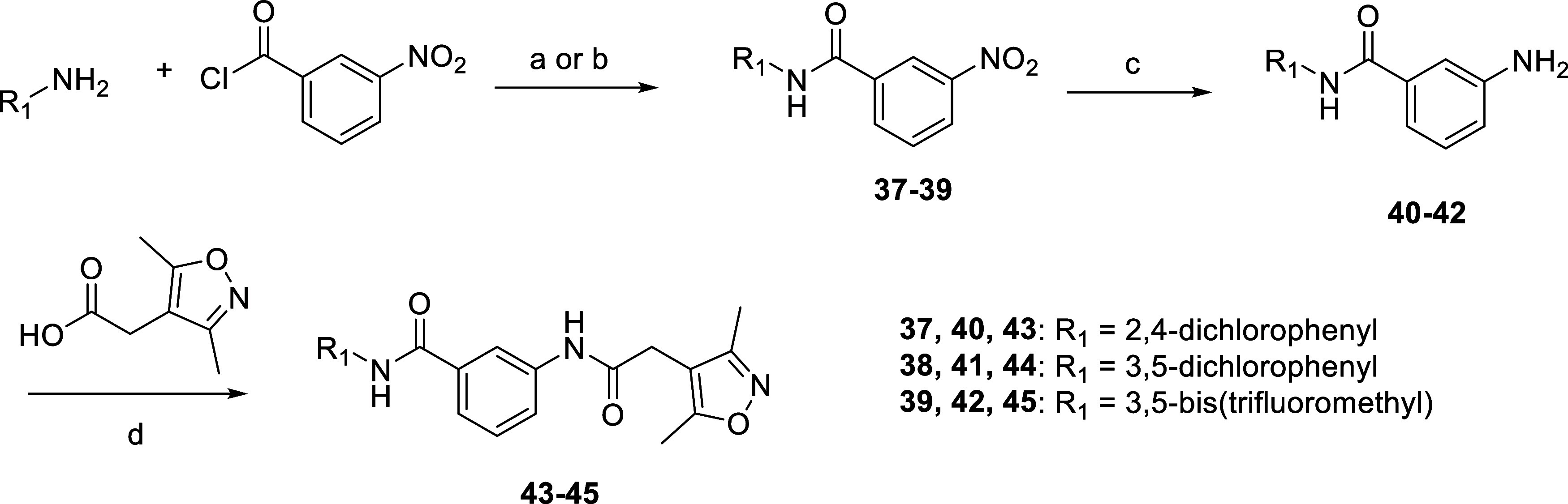
Synthetic Procedure Used for the Synthesis of Compounds **43–45** ^a^

a Conditions. (a) Et_3_N, DCM, rt for **37-38** 48-54% (b) pyridine, rt for **39** 87% (c) SnCl_2_, EtOH/THF, HCl 45-70% (d) HBTU, EDCl, 4-methylmorpholine, DMF 37-67%.

**Scheme 4:**
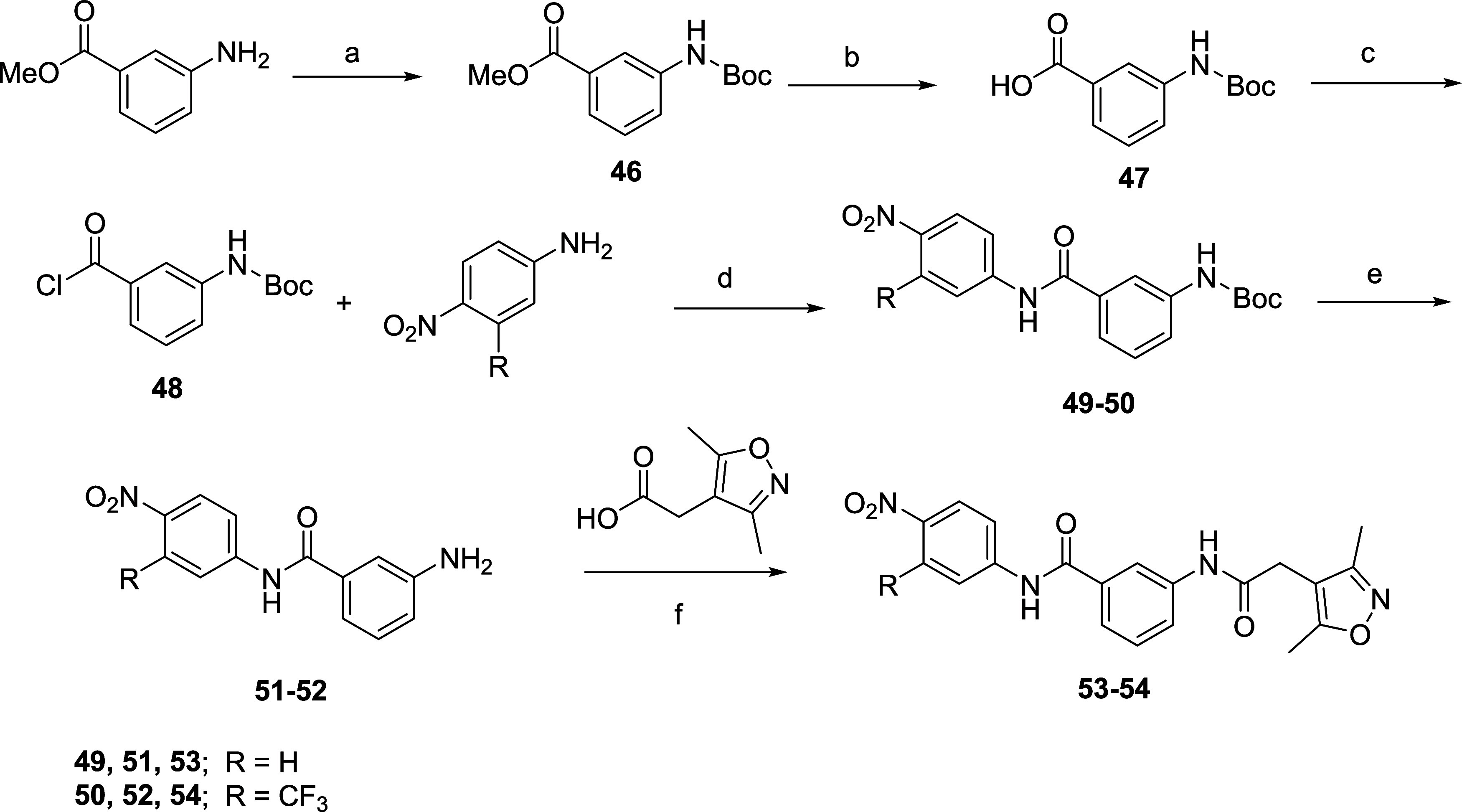
Synthetic Procedure Used for the Synthesis of Compounds **53–54** ^a^

a Conditions. (a) Boc_2_O, THF, 52% (b) LiOH/H_2_O, THF/MeOH, 60 °C, 79% (c) SOCl_2_. DCM, quantitative (d) pyridine, reflux, 31-39% (e) TFA, DCM, 69–100% (f) HBTU, EDCl, 4-methylmorpholine, DMF, 20–34%.

The approach in Scheme 3 cannot be used with nitro-containing anilines because the nitro from the aniline would also get reduced in the second step. Therefore, another synthesis route was developed for final compounds **53** and **54**, where the 3-nitrobenzoyl chloride was substituted by *N*-Boc protected 3-aminobenzoyl chloride (**48**), which was prepared in 3 steps from methyl 3-aminobenzoate (Scheme 4). Compound **48** reacted with the corresponding anilines giving intermediates **49–50**. Then, after Boc deprotection with trifluoroacetic acid, final compounds **53** and **54** were obtained in the usual amide coupling conditions.

Further synthetic efforts were directed to study modifications on the central aromatic ring. Introduction of different pyridine and pyrimidine rings was studied (Schemes 5 and 6) as well as the exchange of the central phenyl ring with furan and imidazole (Scheme 7).

**Scheme 5:**
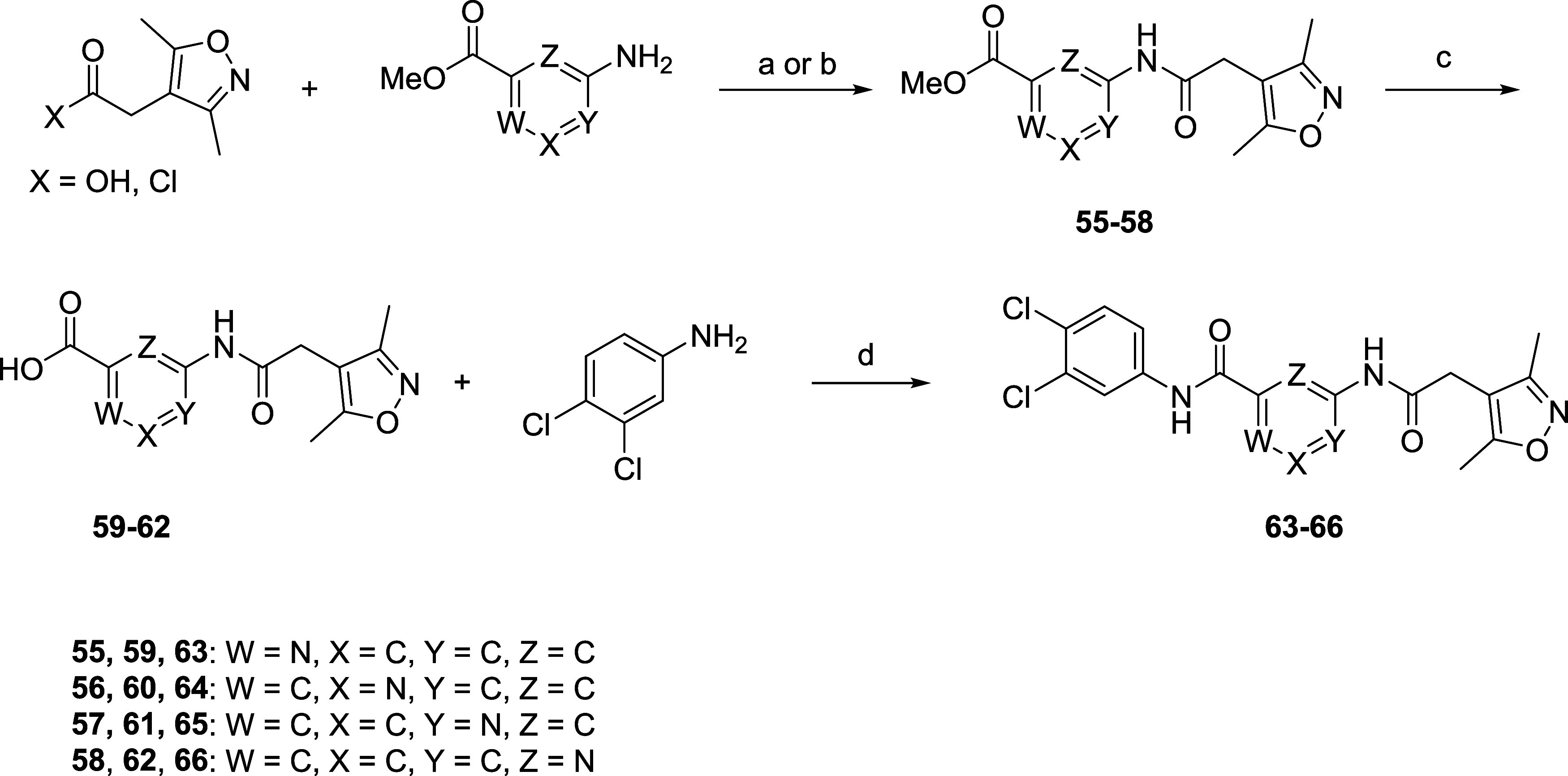
Synthetic Procedure Used for the Synthesis of Compounds **63–66** ^a^

a Conditions. (a) PyBOP, HOBt·H_2_O, DIPEA, THF, MW, 60°C, 33% for **57** (b) DIPEA, DCM, 33–74% for **55**, **56** and **58** (c) LiOH/H_2_O, THF/MeOH, 60 °C, 35-85% (d) HBTU, EDCl, 4-methylmorpholine, DMF, 16-28%.

**Scheme 6:**
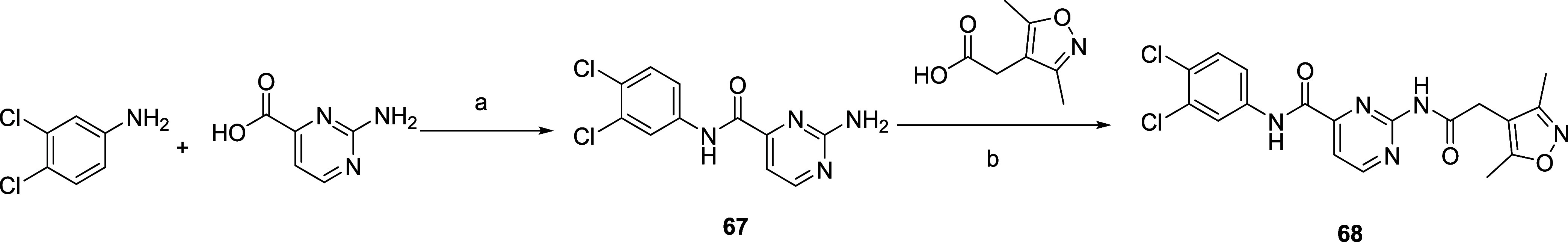
Synthetic Procedure Used for the Synthesis of Compound **68** ^a^

a Conditions. (a) 1. SOCl_2_, DMF 2. DIPEA, DCM, 37% (b) BTFFH, DIPEA, DCM, sealed vial, 80 °C, 16%.

**Scheme 7:**
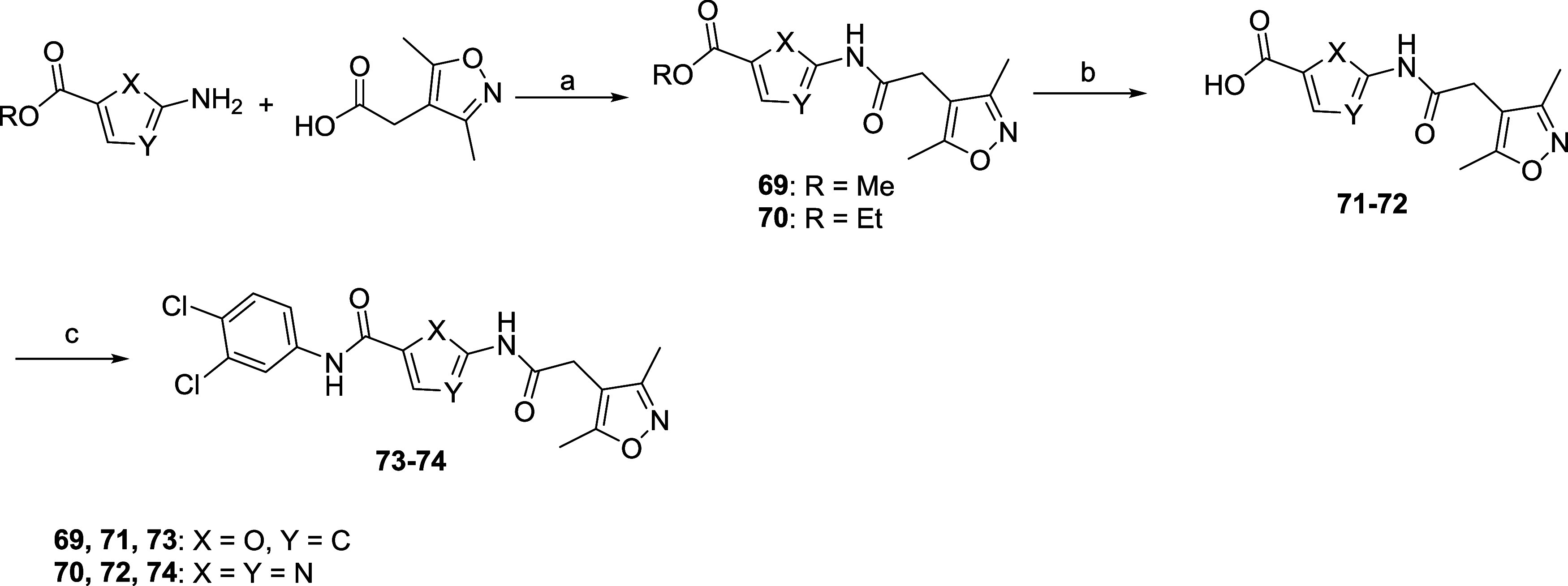
Synthetic Procedure Used for the Synthesis of Compounds **73–74** ^a^

a Conditions. (a) 1. SOCl_2_, DMF 2. DIPEA, DCM, 36% for **69**; BTFFH, DIPEA, DCM, sealed vial, 80 °C, 50% for **70** (b) LiOH/H_2_O, THF/MeOH, 60 °C, 66-87% (c) HBTU, EDCl, 4-methylmorpholine, DMF, 16%.

For the synthesis of derivatives **63–66** a 3-step synthesis route was designed. First, 2-(3,5-dimethylisoxazol-4-yl)­acetic acid, either directly or after it was converted to the corresponding acid chloride, reacted with several commercially available aminopyridines to give intermediates **55–58**. Hydrolysis of the ester group to obtain compounds **59–62** followed by amide coupling reaction with 3,4-dichloroaniline provided final compounds **63–66**.

In case of final compound **68**, with a central pyrimidine ring, the synthesis could be performed in just 2 steps from 2-aminopyrimidine-4-carboxylic acid and 3,4-dichloroaniline by 2 successive amide coupling reactions as depicted in Scheme 6. In the first amide coupling the 2-aminopyridine-4-carboxylic acid was first converted to the acid chloride and then trated with with 3,4-dichloroaniline to obtain intermediate **67** in 37% yield. For the second amide coupling, **67** reacted with 2-(3,5-dimethylisoxazol-4-yl)­acetic acid using fluoro-*N*,*N*,*N*′,*N*′-bis­(tetramethylene)­formamidinium hexafluorophosphate (BTFFH) as a coupling reagent.^14^

The synthesis of compounds bearing a furan or pyrazole central ring was accomplished in a similar manner as described in Scheme 5 but starting with methyl 5-aminofuran-2-carboxylate or ethyl 2-amino-1H-imidazole-5-carboxylate respectively (Scheme 7).

Variations on the linkers connecting the central aromatic ring with the isoxazole ring were also studied. Modification of the original amide linker included inverse amide (Scheme 8), sulfonamide (Scheme 9), amine and sulfamoyl amino moiety (Scheme 10) and ether (Scheme 11).

**Scheme 8:**
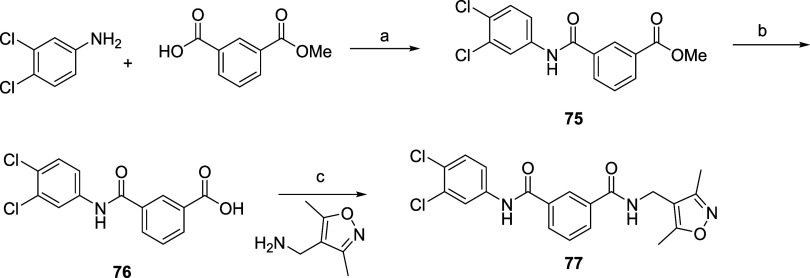
Synthetic Procedure Used for the Synthesis of Compound **77** ^a^

a Conditions. (a) PyBOP, HOBt·H_2_O, DIPEA, THF, MW, 60 °C, 48% (b) LiOH/H_2_O, THF/MeOH, 60 °C, 94% (c) HBTU, EDCl, 4-methylmorpholine, DMF, 69%.

**Scheme 9:**
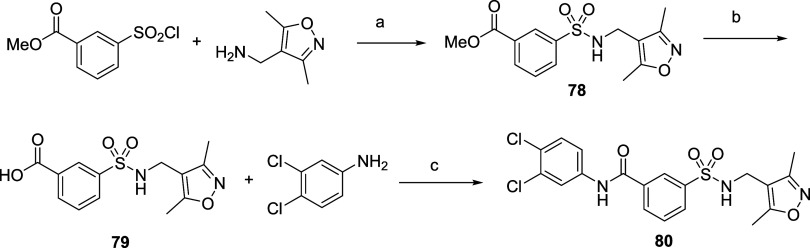
Synthetic Procedure Used for the Synthesis of Compound **80** ^a^

a Conditions. (a) Et_3_N, toluene, 93% (b) 1M LiOH, dioxane, 40 °C, 94% (c) HBTU, EDCl, 4-methylmorpholine, DMF, 10%.

**Scheme 10:**
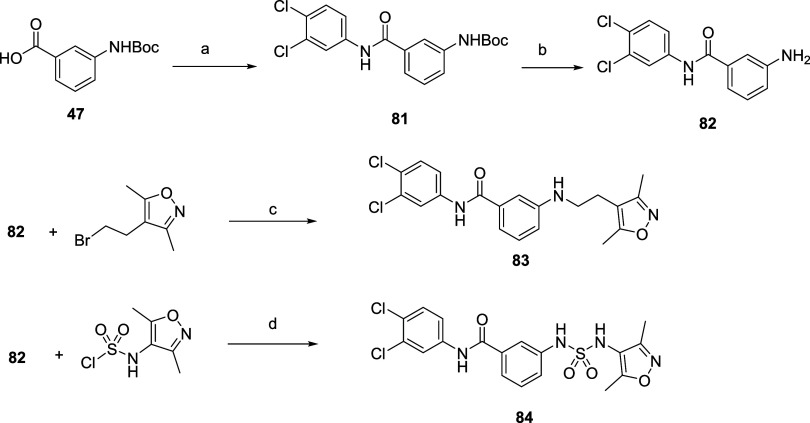
Synthetic Procedure Used for the Synthesis of Compounds **83–84** ^a^

a Conditions. (a) 1. SOCl_2_, DMF 2. 3,4-dichloronaniline, DIPEA, DCM, 44% (b) TFA/DCM, rt, 99% (c) 4-(2-bromoethyl)-3,5-dimethylisoxazole, NaH, DMF, 7% (d) (3,5-dimethylisoxazol-4-yl)­sulfamoyl chloride, Et_3_N, DCM, 12%.

**Scheme 11:**
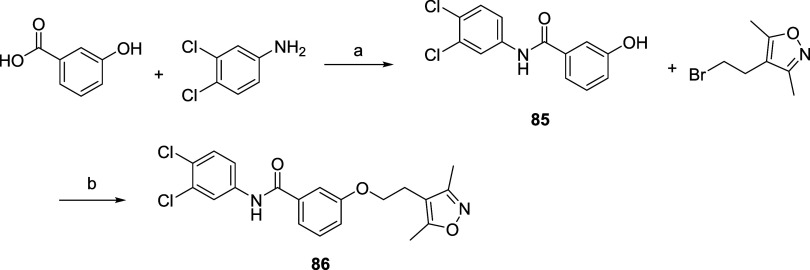
Synthetic Procedure Used for the Synthesis of Compound **86** ^a^

a Conditions. (a) Triphenyl phosphite, toluene, 15% (b) K_2_CO_3_, TBAI, acetone, reflux, 19%.

The inverse amide derivative **77** was easily synthesized in 3 steps from 3-(methoxycarbonyl)­benzoic acid. After the first 2 steps of amide coupling and ester hydrolysis, the second amide coupling took place between **76** and (3,5-dimethylisoxazol-4-yl)­methanamine to obtain **77** in good yield (Scheme 8).

For the synthesis of the sulfonamide derivative **80** methyl 3-(chlorosulfonyl)­benzoate was used as a starting material which reacted with (3,5-dimethylisoxazol-4-yl)­methanamine to provide sulfonamide intermediate **78**. Then, the usual, ester hydrolysis followed by amide coupling with 3,4-dichloroaniline provided the desired product **80** as shown in Scheme 9.

Compounds **83**, with an amine linker, and compound **84**, with a sulfamoyl amino moiety, were prepared from the common intermediate **82** as described in Scheme 10 which was respectively obtained from compound **47** after amide coupling with 3,4-dichloroaniline and Boc deprotection reactions. Then, compound **83** was obtained by a nucleophilic substitution reaction between **82** and 4-(2-bromoethyl)-3,5-dimethylisoxazole using sodium hydride as base. On the other hand, final compound **84** resulted from the reaction of **82** with (3,5-dimethylisoxazol-4-yl)­sulfamoyl chloride.

The derivative with an ether linker **86** was synthesized in 2 steps. First 3-hydroxybenzoic acid reacted with 3,4-dichloroaniline to give compound **85** in a triphenyl phosphite mediated amide coupling reaction.^15^ In the second step, the final compound was obtained by a nucleophilic substitution reaction with potassium carbonate and TBAI in acetone.

One additional final compound (**88**) was prepared with a rigidification of the western amide functionality achieved by benzimidazole formation as depicted in Scheme 12.

**Scheme 12:**
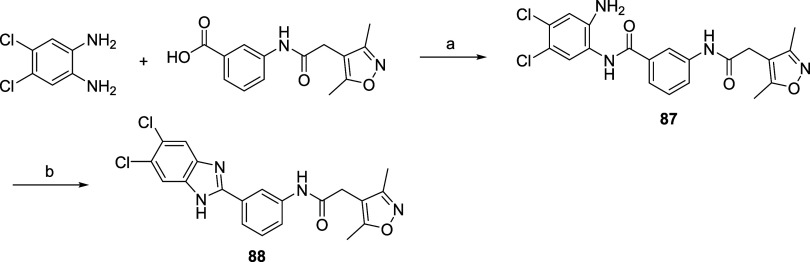
Synthetic Procedure Used for the Synthesis of Compound **88** ^a^

a Conditions. (a) HATU, DIPEA, DMF, 55% (b) Acetic acid, reflux, 65%.

### SAR

For IC_50_ determination, the compounds were tested in the presence of 12.5 μM of G2A agonist (±) 9-HODE at different concentrations (antagonist mode). Antagonists with high potency were also evaluated in absence of 9-HODE in order to check for partial agonism, and in addition on cells that do not harbor the G2A expression cassette as control for G2A independent effects on the cells (CHO-K1 cells with GNA14 only). We observed that compared to GNA14 alone, coexpression of G2A and GNA14 resulted in a slight increase in IP-1 in the absence of any external ligand, indicating a moderate constitutive activity of G2A. Consequently, strong G2A antagonists resulted in a reduction of IP-1 below the baseline of untreated cells. For the first set of compounds the IC_50_ values are presented in Tables 1–3. As observed in Table 1, introduction of substituents on R_2_ (**2–4**) resulted in all cases in slightly inferior G2A inhibition. Therefore, the next derivatives were prepared in absence of substituents on this position.

Likewise, replacing the isoxazole ring in compound **1** with other heterocycles (Table 2) did not improve the inhibitory activity. Only compound **8** (IC_50_ = 3.44 μM) with a 1,2,5-oxadiazole ring showed activity comparable to **1**.

**Table 2:**
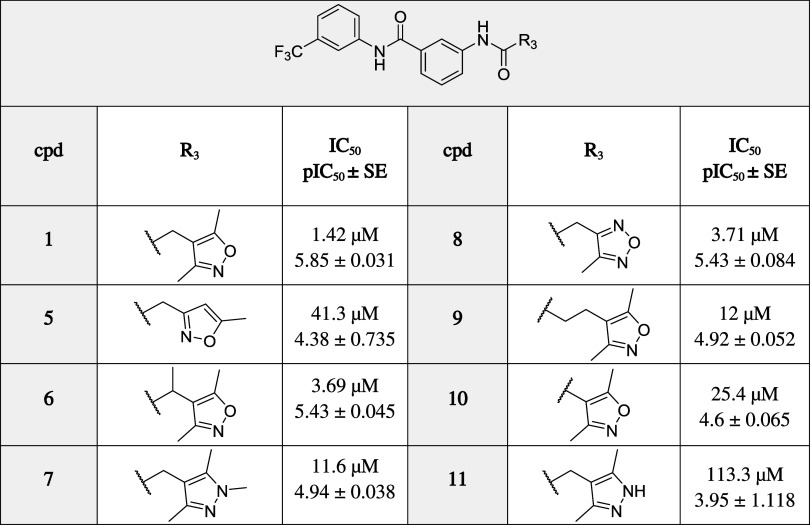
Modification of the Isoxazole Ring (**5–11**)

To finalize this first round of SAR study, 18 compounds with different substituents on the western aromatic ring were analyzed (Table 3). Introduction of substituents of different electronic nature in *para* and *meta* position (compounds **12–17**, **19–21**) were in general tolerated and produced compounds with moderate to good activities. Notably, compound **16**, with a *p*-chloro substituent appeared to be superior to compound **1** with an IC_50_ = 1.00 μM. On the other hand, compound **18** with an ortho fluorine substituent and compound **22** with no substituents showed poor G2A inhibition. Pyridine rings were also studied showing low levels on inhibition for unsubstituted pyridines (compounds **23–25**) and moderate for pyridines bearing the trifluoromethyl group (**26–28**).

**Table 3:**
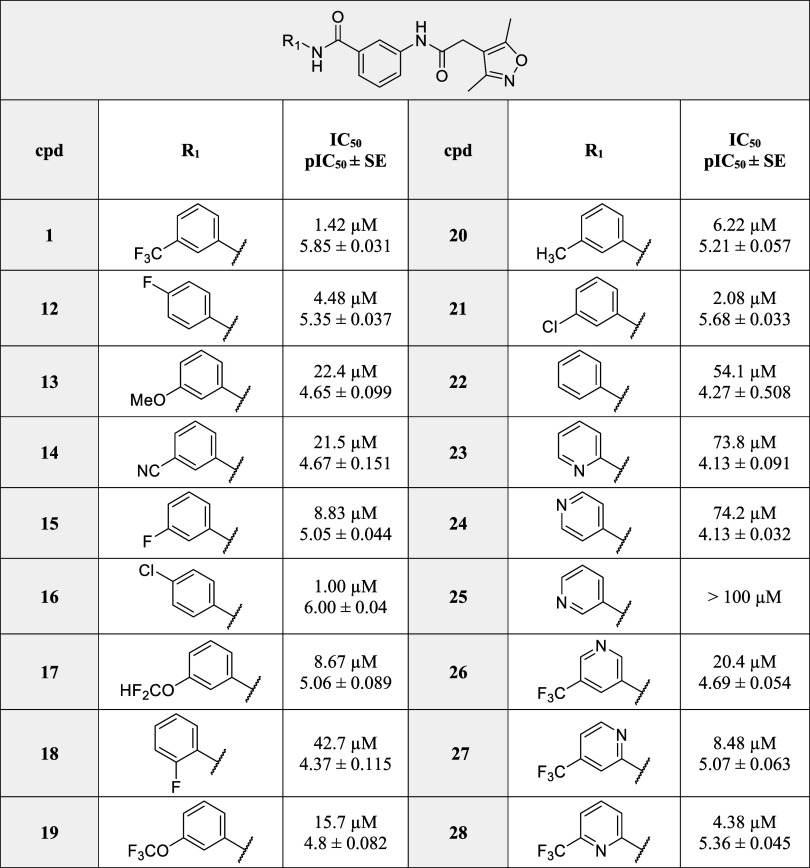
Modifications in the West Aromatic Ring (**12–28**)

The activity results for the new set of modifications on the western aromatic ring are shown in Table 4. Moderate activity was observed with strong electron withdrawing groups in *para* position like in compounds **33** and **53** while 2-naphthyl (**36**) and 2,4-dichlorophenyl (**43**) derivatives were only weak inhibitors. Compound **35** with a 4-chlorobenzyl substituent and also, rather surprisingly, compound **44** with a 3,5-dichlorophenyl moiety were very weak or inactive, indicating a possible space restriction in the protein pocket. As expected, the introduction of substituents in *meta* and *para* position or combination of favorable substituents from the first study resulted in some cases in potent inhibitors like in the case of compounds **32**, **34** or **54** with IC_50_ values around 1–2 μM. Especially compound **31**, with a 3,4-dichlorophenyl substitution, showed strong G2A inhibition with an IC_50_ = 0.33 μM. Therefore, the 3,4-dichlorophenyl ring was incorporated in all next planned derivatives.

**Table 4:**
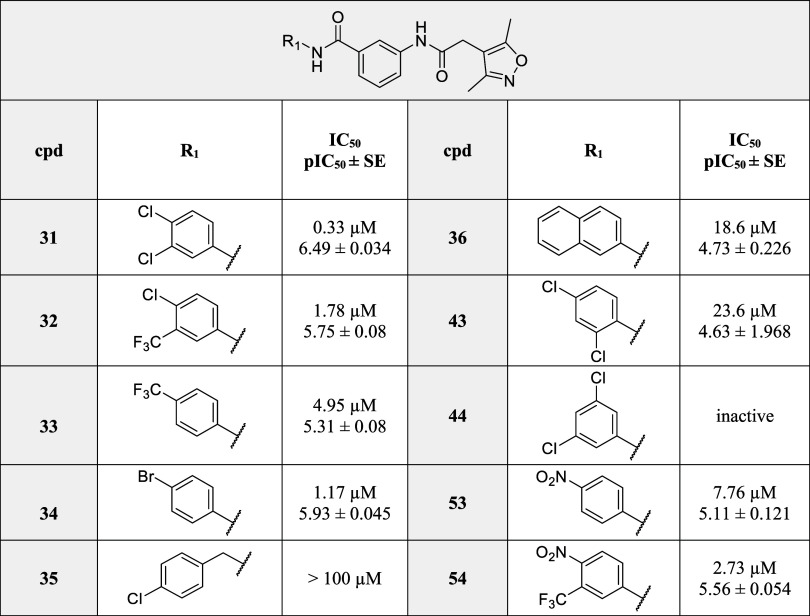
Introduction of Substitution in the Western Aromatic Ring (**31–36, 43–44 and 53–54**)

The activity results for the modifications on the central aromatic ring are shown in Table 5. The position of the nitrogen atom of the central aromatic ring was found to have a high influence on the activity of the compounds. Pyridine derivative **63** and **66** or pyrimidine derivative **68** showed weak or no inhibition activity while pyridine derivative **64** with an IC_50_ = 4.1 μM and in particular **65** with an IC_50_ = 0.69 μM appear to be potent compounds.

**Table 5:**
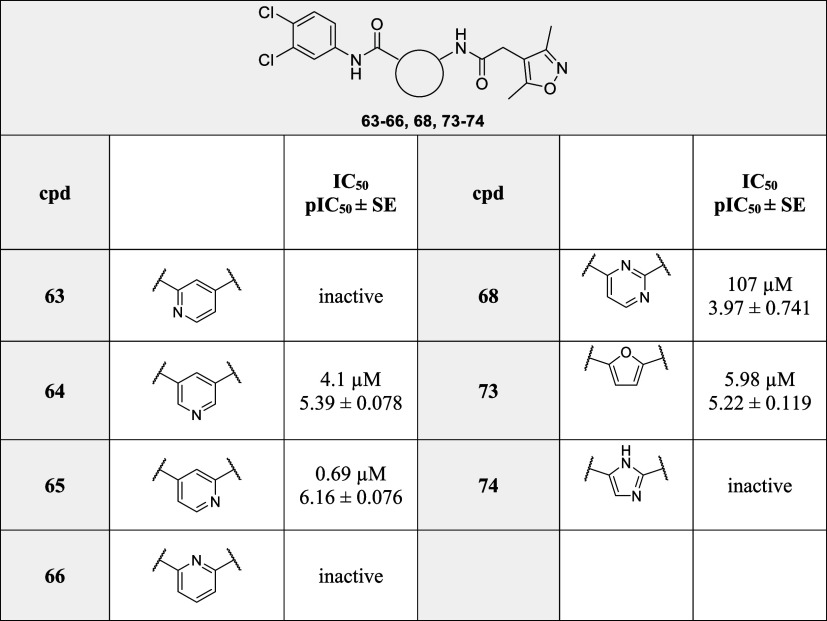
Modifications of the Central Aromatic Ring (**63–66, 68, 73–74**)

The activity results for the modifications on the linkers are shown in Table 6. From the variations on the linkers connecting the central aromatic ring with the isoxazole ring, all tested compounds resulted in weak inhibitors with the exception of compound **83**, missing the carbonyl group of the original amide in **31**, which showed good activity with IC_50_ = 3.27 μM although no improvement. Compound **88**, with a benzimidazole rigidification of the west amide bond also showed weak inhibition.

**Table 6:** Modifications of the Linkers (**77**, **80**, **83**, **84**, **86** and **88**)

cpd	IC_50_ pIC_50_ ± SE	cpd	IC_50_ pIC_50_ ± SE
**77**	56.8 μM 4.25 ± 0.291	**84**	26.9 μM 4.57 ± 0.07
**80**	16.8 μM 4.78 ± 0.04	**86**	inactive
**83**	3.27 μM 5.49 ± 0.058	**88**	14 μM 4.86 ± 0.145

The SARs results are summarized in Figure 3.

**Figure 3:**
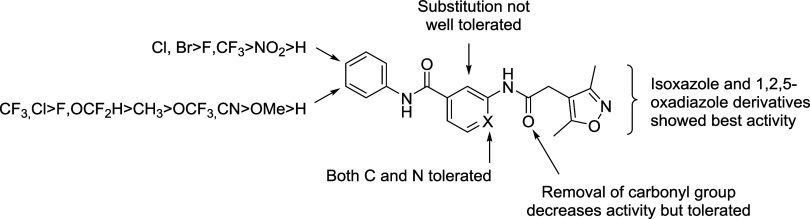
SAR summary.

### Aqueous Solubility and Metabolic Stability

Aqueous solubility and metabolic stability are key determinants of sufficient exposure after administration, as they support good absorption and low clearance. Accordingly, the compounds were evaluated for solubility in PBS buffer and for *in vitro* metabolic half-life in rat liver microsomes, and additionally compound **65** was also assessed in mouse liver microsomes, with the results summarized in Table 7. Compounds **31** and **65** showed high metabolic stability, while introducing the central pyridine ring in **65** compared with **31** did not lead to a notable improvement in solubility.

**Table 7:** Aqueous Solubility and Metabolic Stability of Compounds **31** and **65**

Cmpd.	Aq. solubility^a^ (μM)	Rat liver microsomes^b^ (remaining after 60 min)	Mouse liver microsomes^c^ (remaining after 60 min)
**31**	12–17	98%^d^	
**65**	12–17	86%^d^	76%^d^

a Solubility of compound in PBS buffer, pH 7.4, containing 1% DMSO.

b Microsome mix from the liver of Sprague–Dawley rats.

c Microsome mix from the liver of Balb C mice.

d Percentage of remaining compound after 60 min.

### Evaluation of Selectivity and Cellular Toxicity

Both most promising compounds **31** and **65** were subsequently evaluated in cellular systems. First, cytotoxic potential for long-term cellular treatments has been estimated in Hep-G2 cells using CellTiter-Glo assay (Figure 4). After incubation time of 72 h, neither of the compounds impaired the viability of Hep-G2 cells in concentrations up to 10 μM. However, concentrations >10 μM of all compounds tested led to reduced cell viability.

**Figure 4:**
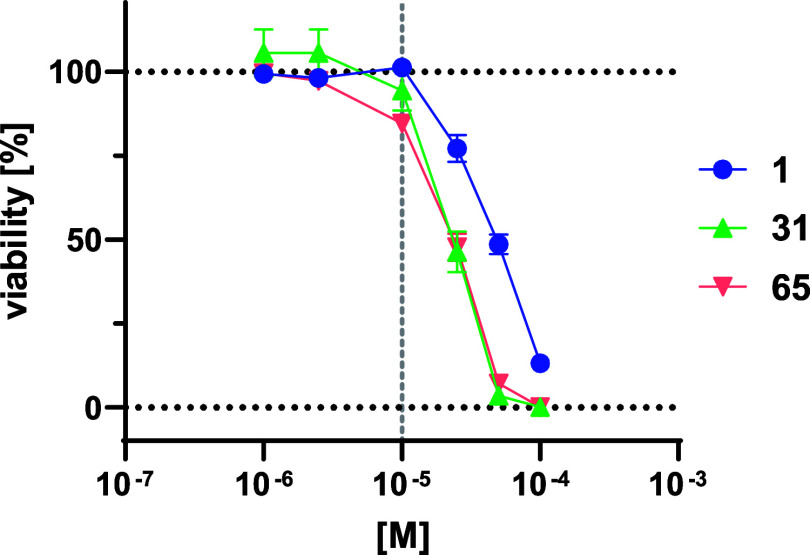
Assessment of the overall toxicity of compounds **1**, **31**, and **65** on Hep-G2 cells was conducted after 72 h of treatment utilizing CellTiter-Glo. The results are presented as mean ± SEM (*n* = 2, with technical replicates *N* = 3 for each).

Dimethyl isoxazoles have been demonstrated to exhibit BET bromodomain activity (*e.g.*, I-BET151 (GSK1210151A)^16^). Therefore, we evaluated the binding of **31** and **65** toward BET bromodomains using differential scanning fluorimetry (DSF) assay, using pan-bromodomain inhibitor JQ1 as reference.^17,18^ **31** did not exhibit any binding toward the BET bromodomains, while **65** showed low stabilization of BRD2 (Table 8).

**Table 8:** Binding of Compounds **31** and **65** towards BET Bromodomain Evaluated Using Differential Scanning Fluorimetry (DSF) Assay^a^

	*T*_m_ [°C]			
	BRDT	BRD2	BRD3	BRD4
**JQ1**	3.78 ± 0.10	6.13 ± 0.02	6.84 ± 0.35	5.88 ± 0.02
**31**	–0.10 ± 0.09	0.09 ± 0.15	–0.44 ± 0.16	0.18 ± 0.14
**65**	1.21 ± 0.14	1.99 ± 0.01	0.74 ± 0.18	1.66 ± 0.08

a Compounds were used at a concentration of 20 μM, recombinant BET bromodomain proteins at 5 μM

To assess their potential as chemical tools, selectivity among GPCRs of compounds **31** and **65** was examined using the PRESTO-Tango assay, which measures β-arrestin recruitment upon activation of a given GPCR. The panel covers over 300 nonolfactory GPCRs. As expected for antagonists, neither compound **31** nor **65** activated G2A. However, both compounds modulated approximately 15 other GPCRs from the panel, with activation defined as ’fold RLU’ (relative light unit) and/or “delta RLU” *versus* vehicle control being greater than three times the standard deviation (SD) (Figure 5A,C). It is important to note the limitations of such a GPCR screen. Antagonist activity is not observed because β-arrestin recruitment generally occurs only when a compound acts as a receptor agonist. In addition, the PRESTO-Tango panel contains many orphan receptors, and for some characterized receptors, activation by reference agonists has not yet been established in this assay. Therefore, only GPCRs that are known to exhibit agonist-mediated response in the PRESTO-Tango assay were selected for retesting (Figure 5B,D). FPR3 (formyl peptide receptor 3) was identified as an off-target for both **31** and **65**. We could demonstrate dose-dependent activation of FPR3 by **31** and **65** (Supporting Information, Figure SI 11), however, cytotoxic effects at concentrations >10 μM did not allow the determination of EC_50_ values.

**Figure 5:**
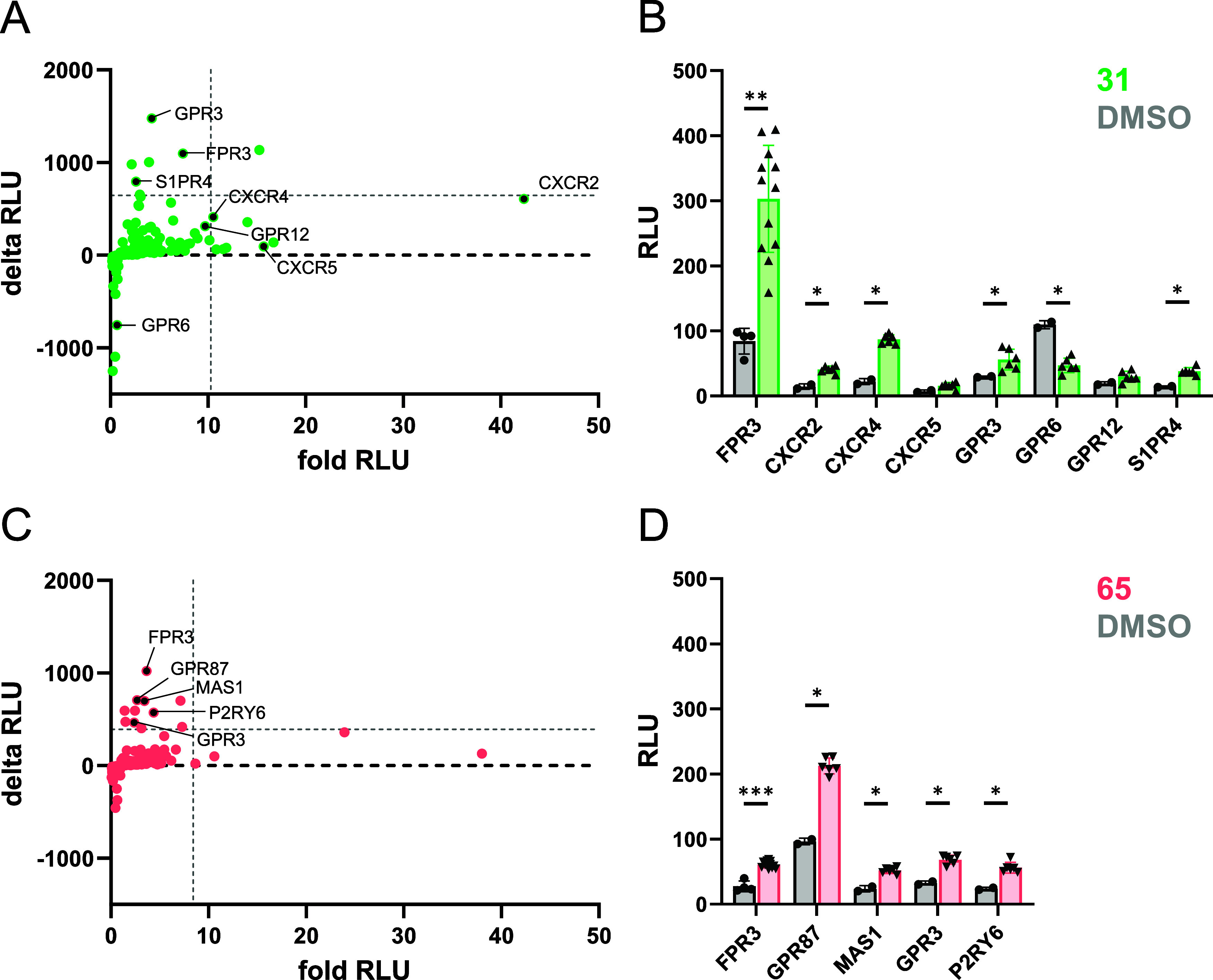
PRESTO-Tango assay as GPCR selectivity screen conducted with 10 μM **31** (A) and 10 μM **65** (C). Selection threshold of 3× SD is shown as light gray dashed line. (B, D) Retest of selected GPCRs with 10 μM **31** (B) or 10 μM **65** (D) *vs* DMSO as vehicle control. One-tailed Mann–Whitney *t* test; *P ≤ 0.1; **P ≤ 0.01; ***P ≤ 0.001.

Overall, selectivity profiling in PRESTO-Tango assay suggested that compound **65** might be a better candidate for *ex vivo* and *in vivo* evaluation due to less off-target activity toward GPCRs.

### *In Vitro* Data in Neurons

Next, the effect of compound **65** and NOX-6–18 on TRPV1 sensitization in primary mouse DRG neurons (Figure 6) was assessed. While a preincubation with 200 nM (±) 9-HODE resulted in a TRPV1 sensitization of 170%, the same treatment together with 100 nM compound **65** was able to significantly revert this effect and lower the sensitization to 118% (Figure 6B). The same sensitization assay was performed with 200 nM (±) 9-HODE and 1 μM NOX-6–18. Here, the (±) 9-HODE-mediated sensitization of the TRPV1 channel could not be modulated, but remained at 200% (Figure 6D). This observation is in line with the lack of activity of NOX-6–18 in the IP-One assay (Table 1 and Supporting Information, Figure S12).

**Figure 6:**
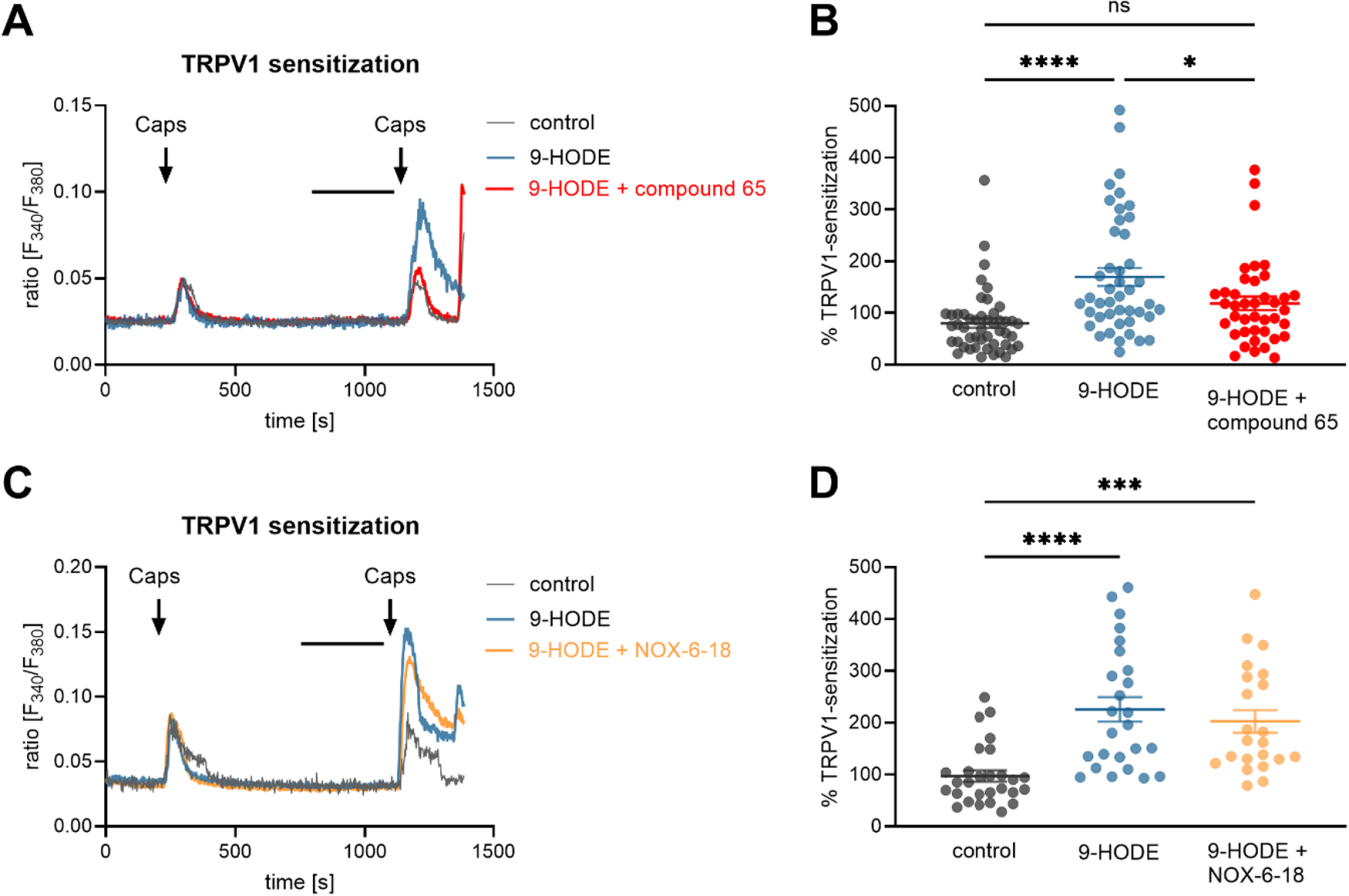
Compound **65** prevents the 9-HODE-mediated sensitization of capsaicin-induced TRPV1 responses in primary sensory neurons, whereas NOX-6–18 has no observable effect. (A, C) Representative traces of capsaicin-induced calcium influxes in primary sensory neurons. In (A), neurons were incubated with control (DMSO, black), ±9-HODE (200 nM, 4 min, blue), or ±9-HODE (200 nM) together with compound **65** (100 nM) for 4 min (red). In (C), neurons were incubated with control (DMSO, black), ±9-HODE (400 nM, 4 min, blue), or ±9-HODE (400 nM) together with NOX-6–18 (1 μM) for 4 min (yellow). Stimulation with capsaicin (50 nM, 30 s) was performed at the indicated time points. (B, D) Statistical analysis of the amplitudes of the capsaicin responses under different treatment conditions. The response of the second capsaicin stimulus was normalized to the response of the first stimulus. Data are shown as mean ± SEM from *n* = 22 to 48 neurons per condition, *p < 0.05, ***p < 0.001, ****p < 0.0001, ns = not significant, one-way ANOVA with Tukeýs post hoc test.

### PK Study

Compound **65**, with its high *in vitro* potency and favorable physicochemical properties, was selected for a pharmacokinetic (PK) study in male CD-1 mice following intraperitoneal (i.p.) administration at 3 mg/kg. Plasma concentrations were measured at 5 min, 15 min, 30 min, 1, 2, 4, and 8 h, with the resulting profiles and calculated PK parameters shown in Figure 7. The study revealed a maximum plasma concentration of 1.88 μM at 30 min, followed by a progressive decline, yet remaining above the IC_50_ for over 3 h (Figure 7, dotted line), positioning **65** as a promising candidate for *in vivo* evaluation of G2A antagonism.

**Figure 7:**
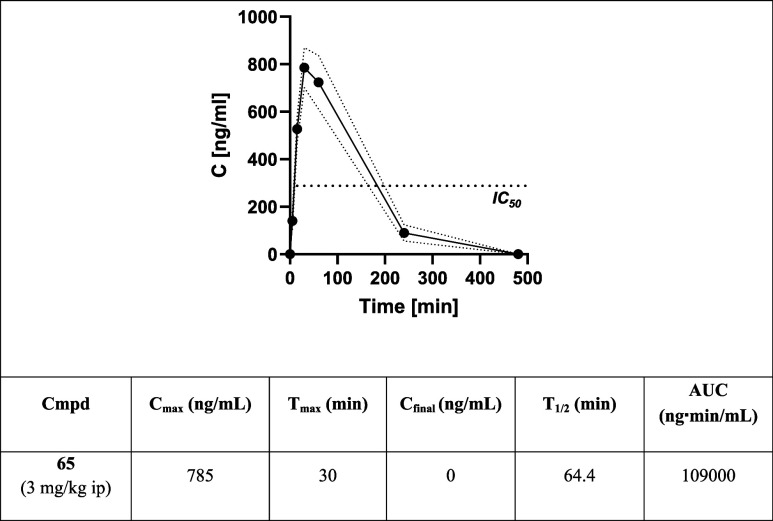
*In vivo* availability of compound **65** ip at 3 mg/kg showed high plasma concentrations and promising *in vivo* pharmacokinetic properties. (Mean ± SD, *n* = 3).

## Conclusions

The discovery of novel scaffolds that inhibit G2A holds substantial promise for diverse therapeutic applications. In this study, we identified selective G2A inhibitors and conducted a comprehensive structure–activity relationship (SAR) analysis of these scaffolds. In total, a series of 51 new compounds was investigated. Among them, compounds **31** and **65** demonstrated excellent activity and high selectivity across GPCRs. Notably, compound **65** also exhibited promising *in vivo* pharmacokinetic (PK) parameters, making it a strong candidate as a tool compound for both *in vitro* and *in vivo* G2A inhibition studies.

## Experimental Section

### General

Chemicals for the synthesis of **2–88** were purchased from Acros Organics (Geel, Belgium), Alfa Aesar GmbH & Co KG (Karlsruhe, Germany), BLD Pharmatech GmbH (Kaiserslautern, Germany), Sigma-Aldrich Chemie GmbH (Steinheim, Germany) and TCI Deutschland (Eschborn, Germany).

Reactions were monitored *via* thin layer chromatography (TLC) using ALUGRAM from Merck (Darmstadt, Germany). To record NMR-spectra, compounds were dissolved in DMSO-*d*_6_ or CDCl_3_ and measured on DPX250, Avance 300 and Avance 400 from Bruker Corporation (Massachusetts, USA) using tetramethylsilane as an internal standard. All chemical shift values are reported in ppm, the multiplicity of the signals assigned as follows: s (singlet), d (duplet), t (triplet) and m (multiplet). Mass spectrometry analysis was performed in positive ion mode by electrospray-ionization (ESI) on a LCMS-2020 single quadrupole MS from Shimadzu (Duisburg, Deutschland). Precision mass was measured using MALDI Orbitrap XL from Life Technologies GmbH (Darmstadt, Germany). For purity estimation of the synthesized compounds, a reverse phase high-performance liquid chromatography (RP-HPLC) was performed using the Luna 10 μm C18(2) 100 Å, LC Column 250 × 4.6 mm^2^ from Phenomenex LTD (Aschaffenburg, Germany) and the analysis was conducted using the Shimadzu prominence module from Shimadzu. Acetonitrile and aqueous formic acid 0.1% were used as eluents. The flow rate was adjusted to 1.0 mL/min and the UV–vis detection occurred at 254 and 280 nm, respectively. Purity of all tested compounds was determined higher than 95%.

### Synthetic Methods

Altogether, 51 new final products were synthesized, analyzed, and tested. Pure compounds (≥95% purity) were obtained after purification by reverse phase HPLC (see Experimental Section). The structures of all final products were confirmed by ^1^H, ^13^C NMR spectroscopy and HPLC analysis coupled to electrospray ionization mass spectrometry (HPLC/ESI-MS) which was also used to determine the purity. Additionally, HRMS was also determined.

### General Procedures

#### GP1: Amide Coupling

GP1a: The corresponding carboxylic acid (1.0 equiv) and HBTU (1.5 equiv) were dissolved in DMF under argon and stirred at rt for 10 min. Then the corresponding amine (1.3 equiv) and 4-methylmorpholine (2.0 equiv) were added at 0 °C and the reaction mixture was stirred at this temperature for 30 min. EDCI·HCl (1.5 equiv) was then added at 0 °C and the reaction mixture was allowed to stir at rt overnight. Solvents were evaporated and the residue was dissolved in water and ethyl acetate. The aqueous phase was extracted with ethyl acetate (3×) and the combined organic layers were dried over MgSO_4_, filtered and evaporated under reduced pressure. The crude product was purified by flash chromatography.

GP1b: The corresponding carboxylic acid (1 equiv) was dissolved in anhydrous dichloromethane and thionyl chloride (15 equiv) were added. The resulting reaction mixture was refluxed for 3 h. Solvents were evaporated and the crude acid chloride was used directly in the next step without further purification. The acid chloride from the previous step (1 equiv) and the corresponding amine (1.3 equiv) were dissolved in anhydrous dichloromethane. The DIPEA (2.0 equiv) was added and the resulting reaction mixture was stirred overnight. After this time, the reaction mixture was diluted with dichloromehane and washed with water and brine. The organic layer was dried over magnesium sulfate, filtered and evaporated. The residue was purified by flash chromatography or preparative HPLC.

GP1c: The corresponding carboxylic acid (1.0 equiv) and amine (1.1 equiv) were dissolved in anhydrous dichloromethane and cooled to 0 °C. Then, a solution of DCC (2.0 equiv) in dichloromethane was slowly added. The resulting mixture was stirred at rt for 5 h. After this time, the reaction mixture was filtered and the solid washed with dichloromethane. The filtrate was then successively washed with a saturated sodium bicarbonate solution, a 1 M hydrochloric acid solution and water. The organic phase was dried over magnesium sulfate, filtered and evaporated. The crude product was purified by preparative HPLC.

GP 1d: To a stirred solution of the corresponding benzoyl chloride (1 equiv) and triethylamine (1 equiv) in anhydrous DCM was added the aniline derivative (1 equiv) at 0 °C. The reaction mixture was allowed to stir at rt overnight. The next day the mixture was diluted with ethyl acetate and washed consecutively with saturated NaHCO_3_, brine and water. The organic layer was dried over MgSO_4_, filtered and concentrated under reduced pressure. The crude product was purified by flash chromatography.

GP1e: (3,5-Dimethylisoxazol-4-yl)­acetic acid (1.3 equiv) and BTFFH (1.5 equiv) were added to a microwave reaction vial (Biotage, 2–5 mL) and diluted in 1 mL anhydrous DCM under argon atmosphere. Subsequently DIPEA (4.5 equiv) was added and the reaction was stirred for 1 h at rt. After addition of the corresponding amine (1.0 equiv) the vial has been sealed and heated to 80 °C for 2–4 days. The reaction was cooled to rt (the pressure was released carefully), diluted with DCM and washed with saturated, aqueous NaHCO_3_ solution (2×) and brine (1×). The organic phase was dried over MgSO_4_, filtered and concentrated in vacuo.

GP 1f: An amine (100 mg), a carboxylic acid (1 mol. equiv to the amine), and DMF (0.5 mL) were placed into a vial. The mixture was then stirred for 30 min at room temperature, and carbonyldiimidazole (CDI, 1.1 mol. equiv to the amine) was added to the vial. The mixture was then stirred in a sealed vial for 24 h at rt. Chloroform (3 mL) was added to the reaction mixture, and it was then washed with water (2 × 1 mL). The chloroform was evaporated under reduced pressure, and 0.6 mL of trifluoroacetic acid was added to the residue. The vial was left shaking for 12 h at room temperature. Then, 3 mL of chloroform was added to the vial, and all the solvent and volatile components were evaporated under reduced pressure to give the crude product. The product was further purified by HPLC.

#### GP2: Ester Hydrolysis

GP2. The corresponding methyl ester was dissolved in THF and 10 equiv LiOH (dissolved in a few uL of water) were added. Methanol was added until a monophasic solution was obtained and the reaction was heated at 60 °C overnight. The solvents were evaporated under reduced pressure and crude product either used in the next step without further purification or purified by preparative HPLC in case of final compounds.

#### GP3: Nitro Reduction

To a solution of the nitro derivative in EtOH and conc. HCl. was added a solution of SnCl_2_ in 3 mL conc. HCl. The reaction mixture was stirred at rt overnight. The reaction mixture was basified with 1 M NaOH and sat. NaHCO_3_ until a pH > 7 was adjusted. The aqueous layer was extracted with ethyl acetate (3 × 20 mL). The combined organic layers were dried over MgSO_4_ and evaporated under reduced pressure.

##### 3-(2-(3,5-Dimethylisoxazol-4-yl)­acetamido)-5-methyl-*N*-(3-(trifluoromethyl)­phenyl)­benzamide (**2**)

The synthesis was done according to GP 1f, starting from 3-[2-(3,5-dimethyl-1,2-oxazol-4-yl)­acetamido]-5-methylbenzoic acid and 3-(trifluoromethyl)­aniline. Yield: 64%; purity, >95% (assessed by LC/MS). ^1^H NMR (500 MHz, DMSO-*d*_6_) δ 10.53 (s, 1H), 10.30 (s, 1H), 8.22 (s, 1H), 8.05 (d, *J* = 8.2 Hz, 1H), 7.91 (s, 1H), 7.69 (s, 1H), 7.60 (t, *J* = 8.0 Hz, 1H), 7.50 (s, 1H), 7.45 (d, *J* = 7.7 Hz, 1H), 3.46 (s, 2H), 2.38 (s, 3H), 2.35 (s, 3H), 2.18 (s, 3H); LC/MS (APSI) *m*/*z* [M + H^+^] calculated for C_22_H_21_F_3_N_3_O_3_: 432.4; found: 432.0.

##### 3-(2-(3,5-Dimethylisoxazol-4-yl)­acetamido)-5-fluoro-*N*-(3-(trifluoromethyl)­phenyl)­benzamide (**3**)

The synthesis was done according to GP 1f, starting from 5-[2-(3,5-dimethyl-1,2-oxazol-4-yl)­acetamido]-3-fluorobenzoic acid and 3-(trifluoromethyl)­aniline. Yield: 78%; purity, >95% (assessed by LC/MS). ^1^H NMR (500 MHz, DMSO-*d*_6_) δ 10.60 (d, *J* = 3.5 Hz, 2H), 8.21 (s, 1H), 8.04 (d, *J* = 8.3 Hz, 1H), 7.89–7.80 (m, 2H), 7.61 (t, *J* = 8.0 Hz, 1H), 7.53 (d, *J* = 9.1 Hz, 1H), 7.48 (d, *J* = 7.9 Hz, 1H), 3.50 (s, 2H), 2.34 (s, 3H), 2.17 (s, 3H); LC/MS (APSI) *m*/*z* [M + H^+^] calculated for C_21_H_18_F_4_N_3_O_3_: 436.4; found: 436.0.

##### 3-(2-(3,5-Dimethylisoxazol-4-yl)­acetamido)-5-(trifluoromethyl)-*N*-(3-(trifluoromethyl)­phenyl)­benzamide (**4**)

The synthesis was done according to GP 1f, starting from 3-[2-(3,5-dimethyl-1,2-oxazol-4-yl)­acetamido]-5-(trifluoromethyl)­benzoic acid and 3-(trifluoromethyl)­aniline. Yield: 55%; purity, >95% (assessed by LC/MS). ^1^H NMR (500 MHz, DMSO-*d*_6_) δ 10.76 (s, 1H), 10.71 (s, 1H), 8.33 (d, *J* = 8.3 Hz, 2H), 8.20 (s, 1H), 8.06 (d, *J* = 8.8 Hz, 2H), 7.63 (t, *J* = 8.0 Hz, 1H), 7.49 (d, *J* = 7.8 Hz, 1H), 3.52 (s, 2H), 2.35 (s, 3H), 2.18 (s, 3H); LC/MS (APSI) *m*/*z* [M + H^+^] calculated for C_22_H_18_F_6_N_3_O_3_: 486.4; found: 486.2.

##### 3-(2-(5-Methylisoxazol-3-yl)­acetamido)-*N*-(3-(trifluoromethyl)­phenyl)­benzamide (**5**)

The synthesis was done according to GP 1f, starting from 2-(5-methyl-1,2-oxazol-3-yl)­acetic acid and 3-amino-*N*-[3-(trifluoromethyl)­phenyl]­benzamide. Yield: 17%; purity, 95% (assessed by LC/MS). ^1^H NMR (500 MHz, DMSO-*d*_6_) δ 10.56 (s, 1H), 10.48 (s, 1H), 8.21 (s, 1H), 8.11 (t, *J* = 1.9 Hz, 1H), 8.05–8.00 (m, 1H), 7.84–7.78 (m, 1H), 7.65 (dd, *J* = 7.6, 1.5 Hz, 1H), 7.58 (t, *J* = 8.0 Hz, 1H), 7.48 (t, *J* = 7.9 Hz, 1H), 7.44 (d, *J* = 7.9 Hz, 1H), 6.23 (d, *J* = 1.2 Hz, 1H), 3.73 (s, 2H), 2.37 (s, 3H); LC/MS (APSI) *m*/*z* [M + H^+^] calculated for C_20_H_17_F_3_N_3_O_3_: 404.4; found: 404.0.

##### 3-(2-(3,5-Dimethylisoxazol-4-yl)­propanamido)-*N*-(3-(trifluoromethyl)­phenyl)­benzamide (**6**)

The synthesis was done according to GP 1f, starting from 2-(3,5-dimethyl-1,2-oxazol-4-yl)­propanoic acid and 3-amino-*N*-[3-(trifluoromethyl)­phenyl]­benzamide. Yield: 70%; purity, >95% (assessed by LC/MS). ^1^H NMR (500 MHz, DMSO-*d*_6_) δ 10.55 (s, 1H), 10.12 (s, 1H), 8.21 (d, *J* = 2.2 Hz, 1H), 8.07 (t, *J* = 1.9 Hz, 1H), 8.02 (d, *J* = 8.5 Hz, 1H), 7.86 (dd, *J* = 8.1, 2.2 Hz, 1H), 7.65 (d, *J* = 7.7 Hz, 1H), 7.58 (t, *J* = 8.0 Hz, 1H), 7.50–7.41 (m, 2H), 3.69 (q, *J* = 7.1 Hz, 1H), 2.37 (s, 3H), 2.23 (s, 3H), 1.37 (d, *J* = 7.1 Hz, 3H); LC/MS (APSI) *m*/*z* [M + H^+^] calculated for C_22_H_21_F_3_N_3_O_3_: 432.2; found: 432.0.

##### *N*-(3-(Trifluoromethyl)­phenyl)-3-(2-(1,3,5-trimethyl-1*H*-pyrazol-4-yl)­acetamido)­benzamide (**7**)

The synthesis was done according to GP 1f, starting from 2-(1,3,5-trimethyl-1*H*-pyrazol-4-yl)­acetic acid and 3-amino-*N*-[3-(trifluoromethyl)­phenyl]­benzamide. Yield: 53%; purity, >95% (assessed by LC/MS). ^1^H NMR (500 MHz, DMSO-*d*_6_) δ 10.54 (s, 1H), 10.18 (s, 1H), 8.21 (d, *J* = 2.1 Hz, 1H), 8.10 (t, *J* = 2.0 Hz, 1H), 8.02 (d, *J* = 8.5 Hz, 1H), 7.82 (dd, *J* = 7.9, 2.1 Hz, 1H), 7.65–7.59 (m, 1H), 7.58 (t, *J* = 8.0 Hz, 1H), 7.48–7.41 (m, 2H), 3.60 (s, 3H), 3.37 (s, 2H), 2.16 (s, 3H), 2.06 (s, 3H); LC/MS (APSI) *m*/*z* [M + H^+^] calculated for C_22_H_22_F_3_N_4_O_2_: 431.2; found: 431.2.

##### 3-(2-(4-Methyl-1,2,5-oxadiazol-3-yl)­acetamido)-*N*-(3-(trifluoromethyl)­phenyl)­benzamide (**8**)

The synthesis was done according to GP 1f, starting from 2-(4-methyl-1,2,5-oxadiazol-3-yl)­acetic acid and 3-amino-*N*-[3-(trifluoromethyl)­phenyl]­benzamide. Yield: 54%; purity, >95% (assessed by LC/MS). ^1^H NMR (500 MHz, DMSO-*d*_6_) δ 10.63 (s, 1H), 10.56 (s, 1H), 8.21 (d, *J* = 2.2 Hz, 1H), 8.11 (t, *J* = 2.0 Hz, 1H), 8.02 (dd, *J* = 8.2, 2.1 Hz, 1H), 7.80 (dd, *J* = 8.1, 2.2 Hz, 1H), 7.70–7.65 (m, 1H), 7.58 (t, *J* = 8.0 Hz, 1H), 7.49 (t, *J* = 7.9 Hz, 1H), 7.44 (d, *J* = 7.9 Hz, 1H), 4.06 (s, 2H), 2.36 (s, 3H); LC/MS (APSI) *m*/*z* [M + H^+^] calculated for C_19_H_16_F_3_N_4_O_3_: 405.3; found: 405.2.

##### 3-(3-(3,5-Dimethylisoxazol-4-yl)­propanamido)-*N*-(3-(trifluoromethyl)­phenyl)­benzamide (**9**)

The synthesis was done according to GP 1f, starting from 3-(3,5-dimethyl-1,2-oxazol-4-yl)­propanoic acid and 3-amino-*N*-[3-(trifluoromethyl)­phenyl]­benzamide. Yield: 33%; purity, >95% (assessed by LC/MS). ^1^H NMR (500 MHz, DMSO-*d*_6_) δ 10.38 (s, 1H), 9.96 (s, 1H), 8.22 (s, 1H), 8.04 (d, *J* = 8.2 Hz, 1H), 7.97 (d, *J* = 8.2 Hz, 1H), 7.91 (s, 1H), 7.61 (d, *J* = 7.2 Hz, 1H), 7.48 (s, 1H), 7.40 (d, *J* = 8.0 Hz, 1H), 7.32 (d, *J* = 7.7 Hz, 1H), 2.65 (s, 4H), 2.33 (s, 3H), 2.21 (s, 3H); LC/MS (APSI) *m*/*z* [M – H^+^] calculated for C_22_H_19_F_3_N_3_O_3_: 430.2; found: 429.6.

##### 3,5-Dimethyl-*N*-(3-((3-(trifluoromethyl)­phenyl)­carbamoyl)­phenyl)­isoxazole-4-carboxamide (**10**)

The synthesis was done according to GP 1f, starting from dimethyl-1,2-oxazole-4-carboxylic acid and 3-amino-*N*-[3-(trifluoromethyl)­phenyl]­benzamide. Yield: 42%; purity, 93% (assessed by LC/MS). ^1^H NMR (500 MHz, DMSO-*d*_6_) δ 10.58 (s, 1H), 10.23 (s, 1H), 8.26–8.18 (m, 2H), 8.04 (d, *J* = 8.0 Hz, 1H), 7.87 (dd, *J* = 8.1, 2.2 Hz, 1H), 7.71 (d, *J* = 7.7 Hz, 1H), 7.63–7.51 (m, 2H), 7.51–7.35 (m, 2H), 2.55 (s, 3H), 2.34 (s, 3H); LC/MS (APSI) *m*/*z* [M – H^+^] calculated for C_20_H_15_F_3_N_3_O_3_: 402.1; found: 402.0.

##### 3-(2-(3,5-Dimethyl-1*H*-pyrazol-4-yl)­acetamido)-*N*-(3-(trifluoromethyl)­phenyl)­benzamide (**11**)

The synthesis was done according to GP 1f, starting from 2-(3,5-dimethyl-1*H*-pyrazol-4-yl)­acetic acid and 3-amino-*N*-[3-(trifluoromethyl)­phenyl]­benzamide. Yield: 28%; purity, >95% (assessed by LC/MS). ^1^H NMR (500 MHz, DMSO-*d*_6_) δ 11.98 (s, 1H), 10.54 (s, 1H), 10.18 (s, 1H), 8.21 (d, *J* = 2.0 Hz, 1H), 8.11 (d, *J* = 2.0 Hz, 1H), 8.02 (d, *J* = 8.6 Hz, 1H), 7.82 (dd, *J* = 8.1, 2.1 Hz, 1H), 7.64–7.59 (m, 1H), 7.57 (d, *J* = 8.1 Hz, 1H), 7.48–7.41 (m, 2H), 3.37 (s, 2H), 2.14 (s, 3H), 2.09 (s, 3H); LC/MS (APSI) *m*/*z* [M + H^+^] calculated for C_21_H_20_F_3_N_4_O_2_: 417.2; found: 417.0.

##### 3-(2-(3,5-Dimethylisoxazol-4-yl)­acetamido)-*N*-(4-fluorophenyl)­benzamide (**12**)

The synthesis was done according to GP 1f, starting from 3-[2-(3,5-dimethyl-1,2-oxazol-4-yl)­acetamido]­benzoic acid and 4-fluoroaniline. Yield: 42%; purity, >95% (assessed by LC/MS). ^1^H NMR (500 MHz, DMSO-*d*_6_) δ 10.37 (s, 1H), 10.32 (s, 1H), 8.09 (s, 1H), 7.86–7.73 (m, 3H), 7.63 (t, *J* = 6.0 Hz, 1H), 7.46 (q, *J* = 7.8, 6.1 Hz, 1H), 7.19 (td, *J* = 8.7, 3.4 Hz, 2H), 3.47 (d, *J* = 3.6 Hz, 2H), 2.34 (d, *J* = 3.7 Hz, 3H), 2.17 (d, *J* = 4.1 Hz, 3H); LC/MS (APSI) *m*/*z* [M + H^+^] calculated for C_20_H_19_FN_3_O_3_: 368.4; found: 368.0.

##### 3-(2-(3,5-Dimethylisoxazol-4-yl)­acetamido)-*N*-(3-methoxyphenyl)­benzamide (**13**)

The synthesis was done according to GP 1f, starting from 3-[2-(3,5-dimethyl-1,2-oxazol-4-yl)­acetamido]­benzoic acid and 3-methoxyaniline. Yield: 59%; purity, >95% (assessed by LC/MS). ^1^H NMR (500 MHz, DMSO-*d*_6_) δ 10.35 (s, 1H), 10.23 (s, 1H), 8.07 (t, *J* = 2.0 Hz, 1H), 7.83 (d, *J* = 8.1 Hz, 1H), 7.62 (d, *J* = 7.5 Hz, 1H), 7.50–7.41 (m, 2H), 7.36 (d, *J* = 8.1 Hz, 1H), 7.24 (td, *J* = 8.1, 1.9 Hz, 1H), 6.68 (dd, *J* = 8.2, 2.4 Hz, 1H), 3.75 (s, 3H), 3.47 (s, 2H), 2.35 (s, 3H), 2.17 (s, 3H); LC/MS (APSI) *m*/*z* [M + H^+^] calculated for C_21_H_22_N_3_O_4_: 380.4; found: 380.0.

##### *N*-(3-Cyanophenyl)-3-(2-(3,5-dimethylisoxazol-4-yl)­acetamido)­benzamide (**14**)

The synthesis was done according to GP 1f, starting from 2-(3,5-dimethyl-1,2-oxazol-4-yl)­acetic acid and 3-amino-*N*-(3-cyanophenyl)­benzamide. Yield: 47%; purity, >95% (assessed by LC/MS). ^1^H NMR (500 MHz, DMSO-*d*_6_) δ 10.56 (s, 1H), 10.36 (s, 1H), 8.22 (d, *J* = 2.2 Hz, 1H), 8.10 (d, *J* = 2.1 Hz, 1H), 8.03–8.00 (m, 1H), 7.86–7.80 (m, 1H), 7.64 (d, *J* = 7.7 Hz, 1H), 7.60–7.53 (m, 2H), 7.47 (t, *J* = 7.9 Hz, 1H), 3.46 (s, 2H), 2.33 (s, 3H), 2.16 (s, 3H); LC/MS (APSI) *m*/*z* [M + H^+^] calculated for C_21_H_19_N_4_O_3_: 375.1; found: 375.2.

##### 3-(2-(3,5-Dimethylisoxazol-4-yl)­acetamido)-*N*-(3-fluorophenyl)­benzamide (**15**)

The synthesis was done according to GP 1f, starting from 2-(3,5-dimethyl-1,2-oxazol-4-yl)­acetic acid and 3-amino-*N*-(3-fluorophenyl)­benzamide. Yield: 46%; purity, >95% (assessed by LC/MS). ^1^H NMR (500 MHz, DMSO-*d*_6_) δ 10.43 (s, 1H), 10.34 (s, 1H), 8.07 (t, *J* = 1.9 Hz, 1H), 7.82 (dd, *J* = 8.2, 2.2 Hz, 1H), 7.71 (dt, *J* = 11.8, 2.2 Hz, 1H), 7.64–7.59 (m, 1H), 7.53 (dd, *J* = 8.4, 2.0 Hz, 1H), 7.46 (t, *J* = 7.9 Hz, 1H), 7.37 (td, *J* = 8.3, 6.8 Hz, 1H), 6.91 (td, *J* = 8.5, 2.6 Hz, 1H), 3.46 (s, 2H), 2.33 (s, 3H), 2.16 (s, 3H); LC/MS (APSI) *m*/*z* [M + H^+^] calculated for C_20_H_19_FN_3_O_3_: 368.1; found: 368.0.

##### *N*-(4-Chlorophenyl)-3-(2-(3,5-dimethylisoxazol-4-yl)­acetamido)­benzamide (**16**)

The synthesis was done according to GP 1f, starting from 3-[2-(3,5-dimethyl-1,2-oxazol-4-yl)­acetamido]­benzoic acid and 4-chloroaniline. Yield: 46%; purity, >95% (assessed by LC/MS). ^1^H NMR (500 MHz, DMSO-*d*_6_) δ 10.39 (s, 1H), 10.36 (s, 1H), 8.09 (t, *J* = 2.0 Hz, 1H), 7.87–7.76 (m, 3H), 7.63 (d, *J* = 7.8 Hz, 1H), 7.47 (t, *J* = 7.9 Hz, 1H), 7.41 (d, *J* = 8.7 Hz, 2H), 3.47 (s, 2H), 2.34 (s, 3H), 2.17 (s, 3H); LC/MS (APSI) *m*/*z* [M + H^+^] calculated for C_20_H_19_ClN_3_O_3_: 384.8; found: 384.0.

##### *N*-(3-(Difluoromethoxy)­phenyl)-3-(2-(3,5-dimethylisoxazol-4-yl)­acetamido)­benzamide (**17**)

The synthesis was done according to GP 1f, starting from 3-[2-(3,5-dimethyl-1,2-oxazol-4-yl)­acetamido]­benzoic acid and 3-(difluoromethoxy)­aniline. Yield: 49%; purity, >95% (assessed by LC/MS). ^1^H NMR (500 MHz, DMSO-*d*_6_) δ 10.43 (s, 1H), 10.36 (s, 1H), 8.10 (s, 1H), 7.83 (d, *J* = 8.2 Hz, 1H), 7.71 (q, *J* = 2.5 Hz, 1H), 7.63 (d, *J* = 7.6 Hz, 2H), 7.47 (td, *J* = 7.9, 2.6 Hz, 1H), 7.39 (td, *J* = 7.9, 2.9 Hz, 1H), 7.22 (d, *J* = 2.7 Hz, 1H), 6.91 (dd, *J* = 8.1, 2.6 Hz, 1H), 3.47 (s, 2H), 2.34 (s, 3H), 2.17 (s, 3H); LC/MS (APSI) *m*/*z* [M + H^+^] calculated for C_21_H_20_F_2_N_3_O_4_: 416.4; found: 416.2.

##### 3-(2-(3,5-Dimethylisoxazol-4-yl)­acetamido)-*N*-(2-fluorophenyl)­benzamide (**18**)

The synthesis was done according to GP 1f, starting from 3-[2-(3,5-dimethyl-1,2-oxazol-4-yl)­acetamido]­benzoic acid and 2-fluoroaniline. Yield: 33%; purity, >95% (assessed by LC/MS). ^1^H NMR (500 MHz, DMSO-*d*_6_) δ 10.33 (s, 1H), 10.09 (s, 1H), 8.10 (s, 1H), 7.82 (d, *J* = 8.1 Hz, 1H), 7.65 (d, *J* = 7.7 Hz, 1H), 7.56 (t, *J* = 7.8 Hz, 1H), 7.44 (t, *J* = 7.9 Hz, 1H), 7.32–7.15 (m, 3H), 3.45 (s, 2H), 2.32 (s, 3H), 2.15 (s, 3H); LC/MS (APSI) *m*/*z* [M + H^+^] calculated for C_20_H_19_FN_3_O_3_: 368.4; found: 368.2.

##### 3-(2-(3,5-Dimethylisoxazol-4-yl)­acetamido)-*N*-(3-(trifluoromethoxy)­phenyl)­benzamide (**19**)

The synthesis was done according to GP 1f, starting from 3-[2-(3,5-dimethyl-1,2-oxazol-4-yl)­acetamido]­benzoic acid and 3-(trifluoromethoxy)­aniline. Yield: 57%; purity, >95% (assessed by LC/MS). ^1^H NMR (500 MHz, DMSO-*d*_6_) δ 10.50 (s, 1H), 10.35 (s, 1H), 8.09 (s, 1H), 7.89 (s, 1H), 7.82 (d, *J* = 8.2 Hz, 1H), 7.78–7.71 (m, 1H), 7.62 (d, *J* = 7.7 Hz, 1H), 7.46 (td, *J* = 8.1, 3.1 Hz, 2H), 7.07 (d, *J* = 8.3 Hz, 1H), 3.46 (s, 2H), 2.33 (s, 3H), 2.15 (s, 3H); LC/MS (APSI) *m*/*z* [M + H^+^] calculated for C_21_H_19_F_3_N_3_O_4_: 434.4; found: 434.2.

##### 3-(2-(3,5-Dimethylisoxazol-4-yl)­acetamido)-*N*-(m-tolyl)­benzamide (**20**)

The synthesis was done according to GP 1f, starting from 3-[2-(3,5-dimethyl-1,2-oxazol-4-yl)­acetamido]­benzoic acid and 3-methylaniline. Yield: 69%; purity, >95% (assessed by LC/MS). ^1^H NMR (500 MHz, DMSO-*d*_6_) δ 10.35 (s, 1H), 10.17 (s, 1H), 8.07 (d, *J* = 2.2 Hz, 1H), 7.83 (d, *J* = 8.2 Hz, 1H), 7.66–7.57 (m, 2H), 7.54 (d, *J* = 8.2 Hz, 1H), 7.45 (td, *J* = 7.9, 2.2 Hz, 1H), 7.22 (td, *J* = 7.6, 2.1 Hz, 1H), 6.91 (d, *J* = 7.6 Hz, 1H), 3.47 (s, 2H), 2.37 (s, 3H), 2.35 (s, 3H), 2.17 (s, 3H); LC/MS (APSI) *m*/*z* [M + H^+^] calculated for C_21_H_22_N_3_O_3_: 364.4; found: 364.2.

##### *N*-(3-Chlorophenyl)-3-(2-(3,5-dimethylisoxazol-4-yl)­acetamido)­benzamide (**21**)

The synthesis was done according to GP 1f, starting from 3-[2-(3,5-dimethyl-1,2-oxazol-4-yl)­acetamido]­benzoic acid and 3-chloroaniline. Yield: 48%; purity, >95% (assessed by LC/MS). ^1^H NMR (500 MHz, DMSO-*d*_6_) δ 10.40 (s, 1H), 10.34 (s, 1H), 8.07 (s, 1H), 7.93 (t, *J* = 2.1 Hz, 1H), 7.82 (d, *J* = 8.2 Hz, 1H), 7.67 (d, *J* = 8.3 Hz, 1H), 7.62 (d, *J* = 7.7 Hz, 1H), 7.46 (t, *J* = 7.9 Hz, 1H), 7.36 (t, *J* = 8.1 Hz, 1H), 7.17–7.11 (m, 1H), 3.45 (s, 2H), 2.33 (s, 3H), 2.15 (s, 3H); LC/MS (APSI) *m*/*z* [M + H^+^] calculated for C_20_H_19_ClN_3_O_3_: 384.8; found: 384.0.

##### 3-(2-(3,5-Dimethylisoxazol-4-yl)­acetamido)-*N*-phenylbenzamide (**22**)

The synthesis was done according to GP 1f, starting from 3-[2-(3,5-dimethyl-1,2-oxazol-4-yl)­acetamido]­benzoic acid and aniline. Yield: 30%; purity, >95% (assessed by LC/MS). ^1^H NMR (500 MHz, DMSO-*d*_6_) δ 10.34 (s, 1H), 10.25 (s, 1H), 8.10 (s, 1H), 7.84 (d, *J* = 8.1 Hz, 1H), 7.76 (d, *J* = 8.0 Hz, 2H), 7.65 (d, *J* = 7.7 Hz, 1H), 7.46 (t, *J* = 7.9 Hz, 1H), 7.34 (t, *J* = 7.7 Hz, 2H), 7.09 (t, *J* = 7.4 Hz, 1H), 3.48 (s, 2H), 2.35 (s, 3H), 2.18 (s, 3H); LC/MS (APSI) *m*/*z* [M + H^+^] calculated for C_20_H_20_N_3_O_3_: 350.4; found: 350.2.

##### 3-(2-(3,5-Dimethylisoxazol-4-yl)­acetamido)-*N*-(pyridin-2-yl)­benzamide (**23**)

The synthesis was done according to GP 1f, starting from 2-(3,5-dimethyl-1,2-oxazol-4-yl)­acetic acid and 3-amino-*N*-(pyridin-2-yl)­benzamide. Yield: 59%; purity, >95% (assessed by LC/MS). ^1^H NMR (500 MHz, DMSO-*d*_6_) δ 10.71 (s, 1H), 10.31 (s, 1H), 8.37 (dd, *J* = 5.1, 1.9 Hz, 1H), 8.18–8.11 (m, 2H), 7.82 (dtd, *J* = 8.9, 7.3, 2.1 Hz, 2H), 7.71 (dd, *J* = 7.9, 1.5 Hz, 1H), 7.42 (t, *J* = 7.9 Hz, 1H), 7.18–7.12 (m, 1H), 3.45 (s, 2H), 2.33 (s, 3H), 2.16 (s, 3H); LC/MS (APSI) *m*/*z* [M + H^+^] calculated for C_19_H_19_N_4_O_3_: 351.4; found: 351.1.

##### 3-(2-(3,5-Dimethylisoxazol-4-yl)­acetamido)-*N*-(pyridin-4-yl)­benzamide (**24**)

The synthesis was done according to GP 1f, starting from 2-(3,5-dimethyl-1,2-oxazol-4-yl)­acetic acid and 3-amino-*N*-(pyridin-4-yl)­benzamide. Yield: 49%; purity, >95% (assessed by LC/MS). ^1^H NMR (500 MHz, DMSO-*d*_6_) δ 10.60 (s, 1H), 10.36 (s, 1H), 8.48–8.43 (m, 2H), 8.10 (d, *J* = 2.0 Hz, 1H), 7.83 (dd, *J* = 8.2, 2.2 Hz, 1H), 7.78–7.73 (m, 2H), 7.63 (d, *J* = 7.7 Hz, 1H), 7.47 (t, *J* = 7.9 Hz, 1H), 3.46 (s, 2H), 2.33 (s, 3H), 2.16 (s, 3H); LC/MS (APSI) *m*/*z* [M + H^+^] calculated for C_19_H_19_N_4_O_3_: 351.4; found: 351.0.

##### 3-(2-(3,5-Dimethylisoxazol-4-yl)­acetamido)-*N*-(pyridin-3-yl)­benzamide (**25**)

The synthesis was done according to GP 1f, starting from 3-[2-(3,5-dimethyl-1,2-oxazol-4-yl)­acetamido]­benzoic acid and pyridin-3-amine. Yield: 60%; purity, >95% (assessed by LC/MS). ^1^H NMR (500 MHz, DMSO-*d*_6_) δ 10.47 (s, 1H), 10.37 (s, 1H), 8.91 (d, *J* = 2.5 Hz, 1H), 8.31 (dd, *J* = 4.7, 1.5 Hz, 1H), 8.21–8.15 (m, 1H), 8.13 (d, *J* = 1.9 Hz, 1H), 7.84 (d, *J* = 8.2 Hz, 1H), 7.66 (d, *J* = 7.7 Hz, 1H), 7.48 (t, *J* = 7.9 Hz, 1H), 7.39 (dd, *J* = 8.3, 4.7 Hz, 1H), 3.47 (s, 2H), 2.35 (s, 3H), 2.17 (s, 3H); LC/MS (APSI) *m*/*z* [M + H^+^] calculated for C_19_H_19_N_4_O_3_: 351.4; found: 351.0.

##### 3-(2-(3,5-Dimethylisoxazol-4-yl)­acetamido)-*N*-(5-(trifluoromethyl)­pyridin-3-yl)­benzamide (**26**)

The synthesis was done according to GP 1f, starting from 3-[2-(3,5-dimethyl-1,2-oxazol-4-yl)­acetamido]­benzoic acid and 5-(trifluoromethyl)­pyridin-3-amine. Yield: 19%; purity, >95% (assessed by LC/MS). ^1^H NMR (500 MHz, DMSO-*d*_6_) δ 10.78 (s, 1H), 10.38 (s, 1H), 9.17 (s, 1H), 8.69 (s, 1H), 8.60 (s, 1H), 8.15 (s, 1H), 7.83 (d, *J* = 8.1 Hz, 1H), 7.67 (d, *J* = 7.7 Hz, 1H), 7.49 (t, *J* = 7.9 Hz, 1H), 3.46 (s, 2H), 2.33 (s, 3H), 2.16 (s, 3H); LC/MS (APSI) *m*/*z* [M + H^+^] calculated for C_20_H_18_F_3_N_4_O_3_: 419.4; found: 419.2.

##### 3-(2-(3,5-Dimethylisoxazol-4-yl)­acetamido)-*N*-(4-(trifluoromethyl)­pyridin-2-yl)­benzamide (**27**)

The synthesis was done according to GP 1f, starting from 3-[2-(3,5-dimethyl-1,2-oxazol-4-yl)­acetamido]­benzoic acid and 4-(trifluoromethyl)­pyridin-2-amine. Yield: 36%; purity, >95% (assessed by LC/MS). ^1^H NMR (500 MHz, DMSO-*d*_6_) δ 11.25 (s, 1H), 10.35 (s, 1H), 8.67 (d, *J* = 5.4 Hz, 1H), 8.52 (d, *J* = 8.9 Hz, 1H), 8.18 (s, 1H), 7.83 (d, *J* = 8.5 Hz, 1H), 7.74 (d, *J* = 8.0 Hz, 1H), 7.53 (d, *J* = 5.4 Hz, 1H), 7.45 (t, *J* = 8.0 Hz, 1H), 3.47 (s, 2H), 2.34 (s, 3H), 2.17 (s, 3H); LC/MS (APSI) *m*/*z* [M + H^+^] calculated for C_20_H_18_F_3_N_4_O_3_: 419.4; found: 419.0.

##### 3-(2-(3,5-Dimethylisoxazol-4-yl)­acetamido)-*N*-(6-(trifluoromethyl)­pyridin-2-yl)­benzamide (**28**)

The synthesis was done according to GP 1f, starting from 3-[2-(3,5-dimethyl-1,2-oxazol-4-yl)­acetamido]­benzoic acid and 6-(trifluoromethyl)­pyridin-2-amine. Yield: 12%; purity, >95% (assessed by LC/MS). ^1^H NMR (500 MHz, DMSO-*d*_6_) δ 11.16 (s, 1H), 10.32 (s, 1H), 8.44 (d, *J* = 8.5 Hz, 1H), 8.20–8.16 (m, 1H), 8.13 (t, *J* = 8.1 Hz, 1H), 7.83 (d, *J* = 8.1 Hz, 1H), 7.74 (d, *J* = 7.7 Hz, 1H), 7.65 (d, *J* = 7.5 Hz, 1H), 7.44 (t, *J* = 7.9 Hz, 1H), 3.47 (s, 2H), 2.35 (s, 3H), 2.18 (s, 3H); LC/MS (APSI) *m*/*z* [M + H^+^] calculated for C_20_H_18_F_3_N_4_O_3_: 419.4; found: 419.1.

##### Methyl 3-(2-(3,5-Dimethylisoxazol-4-yl)­acetamido)­benzoate (**29**)

Synthesized according to GP1a from (3,5-dimethylisoxazol-4-yl)­acetic acid (700 mg, 4.51 mmol), HBTU (2.59 g, 6.77 mmol), methyl 3-aminobenzoate (887 mg, 5.87 mmol), 4-methylmorpholine (992 μL, 9.02 mmol) and EDCI·HCl (1.30 g, 6.77 mmol) in DMF (12.0 mL). Purification by flash chromatography (hexane:EtOAc 7:3) yielded (1.19 g, 91%) of **29**. ^1^H NMR (250 MHz, DMSO-*d*_6_) δ 10.35 (s, 1H), 8.27 (s, 1H), 7.84 (dt, *J* = 8.1, 1.1 Hz, 1H), 7.65 (dt, *J* = 7.7, 1.1 Hz, 1H), 7.46 (t, *J* = 7.9 Hz, 1H), 3.85 (s, 3H), 3.46 (s, 2H), 2.34 (s, 3H), 2.17 (s, 3H).

##### 3-(2-(3,5-Dimethylisoxazol-4-yl)­acetamido)­benzoic Acid (**30**)

Synthesized according to GP2 from **29** (1.19 g, 4.12 mmol) and LiOH (1.01 g, 41.2 mmol) in THF (13.0 mL) to yield 922 mg (82%) of **30**. ^1^H NMR (250 MHz, DMSO-*d*_6_) δ 10.31 (s, 1H), 8.21 (s, 1H), 7.82 (d, *J* = 8.0 Hz, 1H), 7.63 (d, *J* = 7.7 Hz, 1H), 7.43 (t, *J* = 7.8 Hz, 1H), 3.46 (s, 2H), 2.34 (s, 3H), 2.17 (s, 3H).

##### *N*-(3,4-Dichlorophenyl)-3-(2-(3,5-dimethylisoxazol-4-yl)­acetamido)­benzamide (**31**)

Synthesized according to GP1a from **30** (150 mg, 0.547 mmol), HBTU (314 mg, 0.82 mmol), 3,4-dichloroaniline (115 mg, 0.711 mmol), 4-methylmorpholine (120 μL, 1.09 mmol) and EDCI·HCl (157 mg, 0.82 mmol) in DMF (6.0 mL). Purification by preparative HPLC yielded 25.0 mg (11%) of **31**. ^1^H NMR (300 MHz, DMSO-*d*_6_) δ 10.51 (s, 1H), 10.36 (s, 1H), 8.14 (d, *J* = 2.4 Hz, 1H), 8.11 (t, *J* = 1.8 Hz, 1H), 7.86–7.82 (m, 1H), 7.75 (dd, *J* = 8.8, 2.4 Hz, 1H), 7.66–7.59 (m, 2H), 7.48 (t, *J* = 7.8 Hz, 1H), 3.48 (s, 2H), 2.34 (s, 3H), 2.18 (s, 3H); ^13^C NMR (75 MHz, DMSO-*d*_6_) δ 168.8, 166.4, 166.3, 160.2, 139.8, 139.7, 135.6, 131.3, 131.1, 129.4, 125.6, 123.0, 122.8, 121.9, 120.7, 119.2, 109.1, 30.2, 11.2, 10.4; ^*t*^R HPLC: 13.3 min (13 min from 10 to 95% MeCN in water (0.1% formic acid), then 7 min 95% MeCN). 100.0% purity; HRMS (MALDI): *m*/*z* found. 418.0719 [M + H]^+^ (cal. C_20_H_18_Cl_2_N_3_O_3_^+^ 418.0720).

##### *N*-(4-Chloro-3-(trifluoromethyl)­phenyl)-3-(2-(3,5-dimethylisoxazol-4-yl)­acetamido)­benzamide (**32**)

Synthesized according to GP1b from **30** (80 mg, 0.292 mmol) and thionyl chloride (323 μL, 4.38 mmol) in dichloromethane (4.0 mL) for the first step and 4-chloro-3-(trifluoromethyl)­aniline (73.3 mg, 0.380 mmol) and DIPEA (63.7 μL, 0.584 mmol) in DCM (5.0 mL) for the second step. Purification by preparative HPLC yielded 32.0 mg (32%) of **32**. ^1^H NMR (300 MHz, DMSO-*d*_6_) δ 10.64 (s, 1H), 10.37 (s, 1H), 8.33 (d, *J* = 2.5 Hz, 1H), 8.13 (t, *J* = 1.8 Hz, 1H), 8.11 (dd, *J* = 8.8, 2.5 Hz, 1H), 7.84–7.80 (m, 1H), 7.70 (d, *J* = 8.8 Hz, 1H), 7.65 (d, *J* = 8.1 Hz, 1H), 7.48 (t, *J* = 7.9 Hz, 1H), 3.47 (s, 2H), 2.34 (s, 3H), 2.17 (s, 3H); ^13^C NMR (75 MHz, DMSO-*d*_6_) δ 168.8, 166.41, 166.37, 160.2, 139.8, 139.1, 135.4, 132.5, 129.4, 127.1 (q, *J* = 30.5 Hz), 125.4, 125.1, 124.8 (q, *J* = 1.9 Hz), 123.2 (q, *J* = 271 Hz), 123.1, 122.8, 119.4 (q, 5.7 Hz), 119.2, 109.1, 30.2, 11.2, 10.4; ^*t*^R HPLC: 13.5 min (13 min from 10 to 95% MeCN in water (0.1% formic acid), then 7 min 95% MeCN). 100.0% purity; HRMS (MALDI): *m*/*z* found. 452.0979 [M + H]^+^ (cal. C_21_H_18_ClF_3_N_3_O_3_^+^ 452.0983).

##### 3-(2-(3,5-Dimethylisoxazol-4-yl)­acetamido)-*N*-(4-(trifluoromethyl)­phenyl)­benzamide (**33**)

Synthesized according to GP1c from **30** (100 mg, 0.365 mmol), 4-(trifluoromethyl)­aniline (49.9 μL, 0.401 mmol) and DCC (150 mg, 0.729 mmol) in dichloromethane (5.0 mL). Purification by preparative HPLC yielded 49.0 mg (32%) of **33**. ^1^H NMR (300 MHz, DMSO-*d*_6_) δ 10.59 (s, 1H), 10.36 (s, 1H), 8.12 (t, *J* = 1.8 Hz, 1H), 8.01 (d, *J* = 8.4 Hz, 2H), 7.85 (ddd, *J* = 8.1, 2.1, 0.8 Hz, 1H), 7.72 (d, *J* = 8.6 Hz, 2H), 7.66 (dt, *J* = 7.6, 1.3 Hz, 1H), 7.48 (t, *J* = 7.9 Hz, 1H), 3.47 (s, 2H), 2.35 (s, 3H), 2.17 (s, 3H); ^13^C NMR (75 MHz, DMSO-*d*_6_) δ 168.8, 166.5, 166.4, 160.2, 143.3, 139.7, 135.7, 129.4, 126.4 (q, *J* = 3.8 Hz), 124.9 (q, *J* = 269.8 Hz), 124.1 (q, *J* = 31.7 Hz), 122.9, 120.6, 119.3, 109.1, 30.2, 11.2, 10.4; ^*t*^R HPLC: 12.7 min (13 min from 10 to 95% MeCN in water (0.1% formic acid), then 7 min 95% MeCN). 100.0% purity; HRMS (MALDI): *m*/*z* found. 418.1371 [M + H]^+^ (cal. C_21_H_19_F_3_N_3_O_3_^+^ 418.1370).

##### *N*-(4-Bromophenyl)-3-(2-(3,5-dimethylisoxazol-4-yl)­acetamido)­benzamide (**34**)

Synthesized according to GP1a from **30** (80 mg, 0.292 mmol), HBTU (168 mg, 0.438 mmol), 4-bromoaniline (65.2 mg, 0.379 mmol), 4-methylmorpholine (64.1 μL, 0.583 mmol) and EDCI·HCl (83.9 mg, 0.438 mmol) in DMF (5.0 mL). Purification by preparative HPLC yielded 28.0 mg (23%) of **33**. ^1^H NMR (300 MHz, DMSO-*d*_6_) δ 10.37 (s, 1H), 10.34 (s, 1H), 8.09 (t, *J* = 1.7 Hz, 1H), 7.83 (dd, *J* = 8.0, 1.3 Hz, 1H), 7.77–7.72 (m, 2H), 7.63 (d, *J* = 8.0 Hz 1H), 7.55–7.50 (m, 2H), 7.46 (t, *J* = 7.9 Hz, 1H), 3.47 (s, 2H), 2.34 (s, 3H), 2.17 (s, 3H).; ^13^C NMR (75 MHz, DMSO-*d*_6_) δ 168.7, 166.4, 166.0, 160.1, 139.6, 139.0, 135.9, 131.9, 129.3, 129.3, 122.7, 122.6, 119.1, 115.84, 109.1, 30.1, 11.1, 10.4; ^*t*^R HPLC: 12.4 min (13 min from 10 to 95% MeCN in water (0.1% formic acid), then 7 min 95% MeCN). 100.0% purity; HRMS (MALDI): *m*/*z* found. 428.0602 [M + H]^+^ (cal. C_19_H_19_BrN_3_O_3_^+^ 428.0604).

##### *N*-(4-Chlorobenzyl)-3-(2-(3,5-dimethylisoxazol-4-yl)­acetamido)­benzamide (**35**)

Synthesized according to GP1a from **30** (80 mg, 0.292 mmol), HBTU (168 mg, 0.438 mmol), 4-chlorobenzylamine (47.1 μL, 0.379 mmol), 4-methylmorpholine (64.1 μL, 0.583 mmol) and EDCI·HCl (83.9 mg, 0.438 mmol) in DMF (5.0 mL). Purification by preparative HPLC yielded 28.1 mg (33%) of **35**. ^1^H NMR (300 MHz, DMSO-*d*_6_) δ 10.59 (s, 1H), 10.36 (s, 1H), 8.33 (d, *J* = 2.5 Hz, 1H), 8.12 (t, *J* = 1.8 Hz, 1H), 8.00 (d, *J* = 8.6 Hz, 2H), 7.85 (ddd, *J* = 8.1, 2.0, 0.9 Hz, 1H), 7.72 (d, *J* = 8.7 Hz, 2H), 7.66 (dt, *J* = 7.6, 1.3 Hz, 1H), 7.48 (t, *J* = 7.8 Hz, 1H), 3.47 (s, 2H), 2.35 (s, 3H), 2.17 (s, 3H); ^13^C NMR (75 MHz, DMSO-*d*_6_) δ 168.6, 166.6, 166.3, 160.1, 139.5, 139.2, 135.4, 131.7, 129.5, 129.2, 128.6, 122.4, 122.2, 119.0, 109.1, 42.5, 30.1, 11.1, 10.3; ^*t*^R HPLC: 8.8 min (13 min from 10 to 95% MeCN in water (0.1% formic acid), then 7 min 95% MeCN). 96.3% purity; HRMS (MALDI): *m*/*z* found. 398.1265 [M + H]^+^ (cal. C_21_H_21_ClN_3_O_3_^+^ 398.1266).

##### 3-(2-(3,5-Dimethylisoxazol-4-yl)­acetamido)-*N*-(naphthalen-2-yl)­benzamide (**36**)

Synthesized according to GP1a from **30** (54 mg, 0.197 mmol), HBTU (113 mg, 0.295 mmol), 2-naphthylamine (36.6 mg, 0.256 mmol), 4-methylmorpholine (43.3 μL, 0.394 mmol) and EDCI·HCl (56.6 mg, 0.295 mmol) in DMF (4.0 mL). Purification by preparative HPLC yielded 64.2 mg (81%) of **36**. ^1^H NMR (300 MHz, DMSO-*d*_6_) δ 10.46 (s, 1H), 10.36 (s, 1H), 8.43 (d, *J* = 1.4 Hz, 1H), 8.15 (s,1H), 7.91–7.80 (m, 5H), 7.70 (d, *J* = 7.8 Hz, 1H), 7.51–7.40 (m, 3H), 3.49 (s, 2H), 2.35 (s, 3H), 2.19 (s, 3H); ^13^C NMR (75 MHz, DMSO-*d*_6_) δ 168.7, 166.4, 166.2, 160.1, 139.6, 137.2, 136.2, 133.7, 130.4, 129.3, 128.6, 127.9, 127.8, 126.8, 125.2, 122.8, 122.6, 121.4, 119.2, 117.0, 109.2, 30.1, 11.1, 10.4; ^*t*^R HPLC: 12.4 min (13 min from 10 to 95% MeCN in water (0.1% formic acid), then 7 min 95% MeCN). 96.0% purity; HRMS (MALDI): *m*/*z* found. 400.1652 [M + H]^+^ (cal. C_24_H_22_N_3_O_3_^+^ 400.1655).

##### *N*-(2,4-Dichlorophenyl)-3-nitrobenzamide (**37**)

Synthesized according to GP 1d from 3-nitro-benzoyl chloride (200 mg, 1.08 mmol), triethylamine (150 μL, 1.08 mmol), 2,4-dichloroaniline (175 mg, 1.08 mmol) and DCM (10.0 mL). The crude was purified by flash chromatography to yield 160.0 mg (48%) of product as a white solid. ^1^H NMR (250 MHz, DMSO-*d*_6_) δ 10.56 (bs, 1H), 8.80 (t, *J* = 1.8 Hz, 1H), 8.49–8.44 (m, 1 H), 8.43–8.39 (dq, *J* = 7.7, 1.0 Hz, 1H), 7.86 (t, *J* = 8.0 Hz, 1H), 7.75 (d, *J* = 2.3 Hz, 1H), 7.62 (d, *J* = 8.6 Hz, 1H), 7.52–7.48 (dd, *J* = 8.6, 2.3 Hz, 1H).

##### *N*-(3,5-Dichlorophenyl)-3-nitrobenzamide (**38**)

Synthesized according to GP 1d from 3-nitro-benzoyl chloride (200 mg, 1.08 mmol), triethylamine (150 μL, 1.08 mmol), 3,5-dichloroaniline (175 mg, 1.08 mmol) and DCM (10.0 mL). The crude was purified by flash chromatography to yield 181.0 mg (54%) of product as a white solid. ^1^H NMR (250 MHz, DMSO-*d*_6_) δ 10.83 (bs, 1H), 8.80 (t, *J* = 1.9 Hz, 1H), 8.49–8.45 (m, 1H), 8.42–8.39 (dt, *J* = 7.8, 1.3 Hz, 1H), 7.91–7.84 (m, 3H), 7.38 (t, *J* = 1.8 Hz, 1H).

##### *N*-(3,5-Bis­(trifluoromethyl)­phenyl)-3-nitrobenzamide (**39**)

3,5-Bis­(trifluoromethyl)­aniline (200 mg, 0.85 mmol) was dissolved in 5 mL dry pyridine and then 3-nitro-benzoyl chloride (190 mg, 1.03 mmol) was added. After that, the reaction mixture was heated at reflux for 18 h and then poured into ice/H_2_O. The precipitated product was filtered and washed with cold H_2_O to yield 280 mg (87%) of crude product as a white solid which was used in the next step without further purification. ^1^H NMR (250 MHz, DMSO-*d*_6_) δ 11.10 (bs, 1H), 8.85 (t, *J* = 1.9 Hz, 1H), 8.50–8.46 (m, 3H), 8.45–8.41 (m, 1H), 7.91–7.85 (m, 2H).

##### 3-Amino-*N*-(2,4-dichlorophenyl)­benzamide (**40**)

Synthesized according to GP3 from **37** (160 mg, 0.514 mmol), and SnCl_2_ (474 mg, 2.06 mmol) in ethanol/conc. hydrochloric acid (6.0 mL, 1:1). An additional 2.0 mL of THF were added to the reaction mixture because of the low solubility of the starting material in the initial solvents. The product was purified by flash chromatography to yield 65.1 mg (45%) of product as a yellow solid. ^1^H NMR (250 MHz, DMSO-*d*_6_) δ 9.81 (s, 1H), 7.70 (d, *J* = 2.3 Hz, 1H), 7.63 (d, *J* = 8.6 Hz, 1H), 7.45 (dd, *J* = 8.6, 2.3 Hz, 1H), 7.18–7.07 (m, 3H), 6.78–6.74 (m, 1H), 5.32 (br s, 2H).

##### 3-Amino-*N*-(3,5-dichlorophenyl)­benzamide (**41**)

Synthesized according to GP3 from **38** (181 mg, 0.582 mmol), and SnCl_2_ (536 mg, 2.33 mmol) in ethanol/conc. hydrochloric acid (6.0 mL, 1:1). An additional 2.0 mL of THF were added to the reaction mixture because of the low solubility of the starting material in the initial solvents. The product was purified by flash chromatography to yield 82.3 mg (50%) of product as a yellow oil. ^1^H NMR (250 MHz, DMSO-*d*_6_) δ 10.35 (s, 1H), 7.88 (d, *J* = 2.0 Hz, 1H), 7.28 (t, *J* = 1.9 Hz, 1H), 7.17 (t, *J* = 7.8 Hz, 1H), 7.09–7.04 (m, 2H), 6.81–6.76 (m, 1H).

##### 3-Amino-*N*-(3,5-bis­(trifluoromethyl)­phenyl)­benzamide (**42**)

Synthesized according to GP3 from **39** (280 mg, 0.740 mmol), and SnCl_2_ (682 mg, 2.96 mmol) in ethanol/conc. hydrochloric acid (6.0 mL, 1:1). An additional 2.0 mL of THF were added to the reaction mixture because of the low solubility of the starting material in the initial solvents. The product was purified by flash chromatography to yield 180 mg (70%) of product as a yellow solid. ^1^H NMR (250 MHz, DMSO-*d*_6_) δ 10.66 (s, 1H), 8.51 (s, 2H), 7.77 (s, 1H), 7.22–7.09 (m, 3H), 8.81–6.77 (m, 1H), 5.37 (s, 2H).

##### *N*-(2,4-Dichlorophenyl)-3-(2-(3,5-dimethylisoxazol-4-yl)­acetamido)­benzamide (**43**)

Synthesized according to GP1a from (3,5-dimethylisoxazol-4-yl)­acetic acid (27.6 mg, 0.170 mmol), HBTU (102 mg, 0.267 mmol), **40** (65.0 mg, 0.231 mmol), 4-methylmorpholine (39.1 μL, 0.356 mmol) and EDCI·HCl (51.1 mg, 0.267 mmol) in DMF (4.0 mL). Purification by preparative HPLC yielded 50.0 mg (67%) of **43**. ^1^H NMR (300 MHz, DMSO-*d*_6_) δ 10.54 (s, 1H), 10.12 (s, 1H), 8.18 (s,1H), 7.86 (dd, *J* = 7.9, 1.3 Hz, 1H), 7.72–7.59 (m, 3H), 7.48–7.43 (m, 2H), 3.49 (s, 2H), 2.34 (s, 3H), 2.17 (s, 3H); ^13^C NMR (75 MHz, DMSO-*d*_6_) δ 168.8, 166.3, 165.8, 160.1, 139.8, 134.9, 134.8, 131.2, 130.9, 129.9, 129.5, 129.3, 128.1, 123.0, 122.7, 119.3, 109.1, 30.1, 11.1, 10.4; ^*t*^R HPLC: 13.0 min (13 min from 10 to 95% MeCN in water (0.1% formic acid), then 7 min 95% MeCN). 99.3% purity; HRMS (MALDI): *m*/*z* found. 418.0720 [M + H]^+^ (cal. C_20_H_18_Cl_2_N_3_O_3_^+^ 418.0720).

##### *N*-(3,5-Dichlorophenyl)-3-(2-(3,5-dimethylisoxazol-4-yl)­acetamido)­benzamide (**44**)

Synthesized according to GP1a from (3,5-dimethylisoxazol-4-yl)­acetic acid (37.7 mg, 0.243 mmol), HBTU (140 mg, 0.365 mmol), **41** (82.0 mg, 0.292 mmol), 4-methylmorpholine (53.4 μL, 0.486 mmol) and EDCI·HCl (69.9 mg, 0.365 mmol) in DMF (5.0 mL). Purification by preparative HPLC yielded 38.3 mg (37%) of **44**. ^1^H NMR (300 MHz, DMSO-*d*_6_) δ 10.53 (s, 1H), 10.36 (s, 1H), 8.11 (t, *J* = 1.8 Hz, 1H), 7.88–7.83 (m, 3H), 7.63 (d, *J* = 7.8 Hz, 1H), 7.48 (t, *J* = 7.8 Hz, 1H), 7.31 (t, *J* = 1.8 Hz, 1H), 3.47 (s, 2H), 2.34 (s, 3H), 2.17 (s, 3H); ^13^C NMR (75 MHz, DMSO-*d*_6_) δ 168.8, 166.4, 160.3, 141.9, 139.7, 135.3, 134.4, 129.4, 123.3, 123.1, 122.8, 119.1, 118.7, 109.0, 30.1, 11.1, 10.4; ^*t*^R HPLC: 13.8 min (13 min from 10 to 95% MeCN in water (0.1% formic acid), then 7 min 95% MeCN). 99.4% purity; HRMS (MALDI): *m*/*z* found. 418.0720 [M + H]^+^ (cal. C_20_H_18_Cl_2_N_3_O_3_^+^ 418.0720).

##### *N*-(3,5-Bis­(trifluoromethyl)­phenyl)-3-(2-(3,5-dimethylisoxazol-4-yl)­acetamido)­benzamide (**45**)

Synthesized according to GP1a from (3,5-dimethylisoxazol-4-yl)­acetic acid (66.8 mg, 0.431 mmol), HBTU (248 mg, 0.646 mmol), **42** (180 mg, 0.517 mmol), 4-methylmorpholine (94.7 μL, 0.861 mmol) and EDCI·HCl (124 mg, 0.646 mmol) in DMF (6.0 mL). Purification by preparative HPLC yielded 128 mg (61%) of **45**. ^1^H NMR (300 MHz, DMSO-*d*_6_) δ 10.84 (s, 1H), 10.38 (s, 1H), 8.50 (s, 2H), 8.18 (s, 1H), 7.84 (dd, *J* = 7.8, 1.5 Hz, 1H), 7.79 (s, 1H), 7.69 (dt, *J* = 7.9 Hz, 1H), 7.51 (t, *J* = 7.8 Hz, 1H), 3.48 (s, 2H), 2.35 (s, 3H), 2.18 (s, 3H); ^13^C NMR (75 MHz, DMSO-*d*_6_) δ 168.8, 166.6, 166.4, 160.2, 141.6, 139.8, 135.1, 131.2 (q, *J* = 32.6 Hz), 129.5, 123.8, (q, *J* = 271.1 Hz) 123.3, 122.9, 120.4, 119.2, 116.9 (q, *J* = 3.5 Hz), 109.1, 30.2, 11.1, 10.4; ^*t*^R HPLC: 14.3 min (13 min from 10 to 95% MeCN in water (0.1% formic acid), then 7 min 95% MeCN). 100.0% purity; HRMS (MALDI): *m*/*z* found. 486.1240 [M + H]^+^ (cal. C_22_H_18_F_6_N_3_O_3_^+^ 486.1246).

##### Methyl 3-((*tert*-Butoxycarbonyl)­amino)­benzoate (**46**)

To a solution of methyl-3-aminobezoate (800 mg, 5.29 mmol) in THF (10.0 mL) were added Boc_2_O (1.39 g, 6.35 mmol) and trimethylamine (1.04 mL, 7.41 mmol) at 0 °C. The reaction mixture was allowed to stir at rt overnight. The solvent was evaporated under reduced pressure. The residue was dissolved in ethyl acetate and the organic layer was washed with NaHCO_3_, water and brine. The organic layer was dried over MgSO_4_, filtered and evaporated. The residue was purified by column chromatography to yield 687 mg (52%) of **46**. ^1^H NMR (250 MHz, DMSO-*d*_6_) δ 9.57 (s, 1H), 8.19 (t, *J* = 1.8 Hz, 1H), 7.63 (dq, *J* = 8.1, 1.1 Hz, 1H), 7.55 (dt, *J* = 7.8, 1.3 Hz, 1H), 7.38 (t, *J* = 7.8 Hz, 1H), 3.84 (s, 3H), 1.48 (s, 9H).

##### 3-((*tert*-Butoxycarbonyl)­amino)­benzoic Acid (**47**)

Synthesized according to GP2 from **46** (654 mg, 2.60 mmol) and LiOH (636 mg, 26.0 mmol) in THF (8.0 mL) to yield 490 mg (79%) of **47**. ^1^H NMR (250 MHz, DMSO-*d*_6_) δ 12.87 (bs, 1H), 9.53 (s, 1H), 8.14 (t, *J* = 1.8 Hz, 1H), 7.62 (dq, *J* = 8.1, 1.1 Hz, 1H), 7.53 (dt, *J* = 7.8, 1.3 Hz, 1H), 7.35 (t, *J* = 7.8 Hz, 1H), 1.48 (s, 9H).

##### *tert*-Butyl (3-(Chlorocarbonyl)­phenyl)­carbamate (**48**)

**47** (150 mg, 0.632 mmol) was dissolved in dichloromethane (4.0 mL) and thionyl chloride (466 μL, 6.32 mmol) was added. The reaction mixture was refluxed for 2 h. The solvent was evaporated and the crude product was used in the next step without further purification.

##### *tert*-Butyl (3-((4-Nitrophenyl)­carbamoyl)­phenyl)­carbamate (**49**)

A solution of **48** (122 mg, 0.48 mmol) in anhydrous pyridine (2.0 mL) was added dropwise to a solution of 4-nitroaniline (101 mg, 0.48 mmol) in 5.0 mL anhydrous pyridine and the mixture was stirred overnight at reflux. The reaction mixture was diluted with water and the aqueous layer was extracted with ethyl acetate (3×). The organic layer was dried over MgSO_4_, filtered and evaporated under reduced pressure. The crude was purified by flash chromatography to yield 67.0 mg (39%) of product as a yellow solid. ^1^H NMR (250 MHz, DMSO-*d*_6_) δ 10.79 (bs, 1H), 9.58 (s, 1H), 8.29–8.23 (m, 2H), 8.03–8.01 (m, 3H), 7.65–7.61 (m, 1H), 7.56 (dt, *J* = 7.8, 1.2 Hz, 1H), 7.43 (t, *J* = 7.8 Hz, 1H), 1.49 (s, 9H).

##### *tert*-Butyl (3-((4-Nitro-3-(trifluoromethyl)­phenyl)­carbamoyl)­phenyl)­carbamate (**50**)

A solution of **48** (250 mg, 0.978 mmol) in anhydrous pyridine (5.0 mL) was added dropwise to a solution of 4-nitro-3-(trifluoromethyl)­aniline (168 mg, 0.815 mmol) 5.0 mL anhydrous pyridine and the mixture was stirred overnight at reflux. The reaction mixture was diluted with water and the aqueous layer was extracted with ethyl acetate (3×). The organic layer was dried over MgSO_4_, filtered and evaporated under reduced pressure. The crude was purified by flash chromatography to yield 126 mg (31%) of product as a yellow solid. ^1^H NMR (250 MHz, DMSO-*d*_6_) δ 12.85 (bs, 1H), 10.96 (s, 1H), 9.60 (s,1H), 9.52 (s,1H), 8.46 (d, *J* = 2.0 Hz, 1H), 8.35–8.23 (m, 2H), 8.12 (dt, *J* = 9.6, 1.8 Hz, 2H), 1.48 (s, 9H).

##### 3-Amino-*N*-(4-nitrophenyl)­benzamide (**51**)

Trifluoroacetic acid (0.7 mL) was added to a cooled solution of **49** (67 mg, 0.187 mmol) in dichloromethane (3.0 mL) at 0 °C. The ice bath was removed and the reaction mixture was allowed to stir at rt overnight. The solvent was removed under reduced pressure and the residue was dissolved in saturated aqueous sodium bicarbonate and extracted with ethyl acetate (3×). The combined organic layers were dried over magnesium sulfate, filtered and evaporated to yield 33.0 mg (69%) of crude product that was used in the next step without further purification. ^1^H NMR (250 MHz, DMSO-*d*_6_) δ 10.65 (s, 1H), 8.26 (m, 2H), 8.05 (m, 2H), 7.18 (t, *J* = 7.5 Hz, 1H), 7.09 (m, 2H), 6.79 (m, 1H), 5.37 (s, 2H).

##### 3-Amino-*N*-(4-nitro-3-(trifluoromethyl)­phenyl)­benzamide (**52**)

Trifluoroacetic acid (1.0 mL) was added to a cooled solution of **50** (126 mg, 0.296 mmol) in dichloromethane (5.0 mL) at 0 °C. The ice bath was removed and the reaction mixture was allowed to stir at rt overnight. The solvent was removed under reduced pressure and the residue was dissolved in saturated aqueous sodium bicarbonate and extracted with ethyl acetate (3×). The combined organic layers were dried over magnesium sulfate, filtered and evaporated to yield 96.1 mg (100%) of crude product that was used in the next step without further purification. ^1^H NMR (250 MHz, DMSO-*d*_6_) δ 10.82 (s, 1H), 8.45 (d, *J* = 1.8 Hz, 1H), 8.33 (dd, *J* = 9.0, 2.0 Hz, 1H), 8.23 (d, *J* = 9.0 Hz, 1H), 7.22–7.09 (m, 3H), 6.80 (dt, *J* = 7.6, 1.5, Hz, 1H), 5.39 (s, 2H).

##### 3-(2-(3,5-Dimethylisoxazol-4-yl)­acetamido)-*N*-(4-nitrophenyl)­benzamide (**53**)

Synthesized according to GP1a from (3,5-dimethylisoxazol-4-yl)­acetic acid (19.9 mg, 0.128 mmol), HBTU (73.7 mg, 0.192 mmol), **51** (33.0 mg, 0.128 mmol), 4-methylmorpholine (28.2 μL, 0.257 mmol) and EDCI·HCl (36.9 mg, 0.192 mmol) in DMF (4.0 mL). Purification by preparative HPLC yielded 10.1 mg (20%) of **53**. ^1^H NMR (300 MHz, DMSO-*d*_6_) δ 10.81 (s, 1H), 10.37 (s, 1H), 8.14 (m, 5H), 7.83 (s, 1H), 7.66 (s, 1H), 7.49 (s, 1H), 3.47 (s, 2H), 2.34 (s, 3H), 2.17 (s, 3H); ^13^C NMR (75 MHz, DMSO-*d*_6_) δ 168.8, 166.6, 166.4, 160.1, 141.5, 139.8, 135.0, 131.7, 131.3, 130.9, 130.4, 129.4, 125.5, 123.3, 122.8, 121.9, 120.3, 119.4, 116.8, 109.0, 30.1, 11.1, 10.3; ^*t*^R HPLC: 11.8 min (13 min from 10 to 95% MeCN in water (0.1% formic acid), then 7 min 95% MeCN). 95.7% purity; HRMS (MALDI): *m*/*z* found. 395.1344 [M + H]^+^ (cal. C_20_H_19_N_4_O_5_^+^ 395.1350).

##### 3-(2-(3,5-Dimethylisoxazol-4-yl)­acetamido)-*N*-(4-nitro-3-(trifluoromethyl)­phenyl)­benzamide (**54**)

Synthesized according to GP1a from (3,5-dimethylisoxazol-4-yl)­acetic acid (37.8 mg, 0.243 mmol), HBTU (140 mg, 0.365 mmol), **52** (95.0 mg, 0.292 mmol), 4-methylmorpholine (53.5 μL, 0.487 mmol) and EDCI·HCl (70.0 mg, 0.365 mmol) in DMF (5.0 mL). Purification by preparative HPLC yielded 38.0 mg (34%) of **54**. ^1^H NMR (300 MHz, DMSO-*d*_6_) δ 10.98 (s, 1H), 10.38 (s, 1H), 8.45 (d, *J* = 1.9 Hz, 2H), 8.34 (dd, *J* = 9.0, 2.1 Hz, 1H), 8.24 (d, *J* = 9.0 Hz, 1H), 8.17 (t, *J* = 1.7 Hz, 1H), 7.85 (dd, *J* = 8.0, 1.5 Hz, 1H), 7.69 (d, *J* = 7.8 Hz, 1H), 7.51 (t, *J* = 8.0 Hz, 1H), 3.48 (s, 2H), 2.34 (s, 3H), 2.17 (s, 3H); ^13^C NMR (75 MHz, DMSO-*d*_6_) δ 168.9, 166.8, 166.4, 160.2, 144.4, 142.1, 139.8, 135.0, 129.5, 128.0, 123.7, 123.4, 123.2 (q, *J* = 33.3 Hz), 122.1 (q, *J* = 271.3 Hz), 119.3, 118.9 (q, *J* = 5.8 Hz), 109.1, 30.2, 11.2, 10.4; ^*t*^R HPLC: 13.0 min (13 min from 10 to 95% MeCN in water (0.1% formic acid), then 7 min 95% MeCN). 100.0% purity; HRMS (MALDI): *m*/*z* found. 463.1219 [M + H]^+^ (cal. C_21_H_18_F_3_N_4_O_5_^+^ 463.1223).

##### Methyl 4-(2-(3,5-Dimethylisoxazol-4-yl)­acetamido)­picolinate (**55**)

2-(3,5-Dimethylisoxazol-4-yl)­acetyl chloride (178 mg, 1.03 mmol) was dissolved in dichloromethane (2.0 mL) and added dropwise to a solution of methyl-4-aminopyridine-2-carboxylate (207 mg, 1.33 mmol) and DIPEA (269 μL, 1.54 mmol) in dichloromethane (3.0 mL). The reaction was allowed to stir overnight at rt under inert atmosphere. The reaction mixture was diluted with dichloromethane and washed with water and brine consecutively. The organic extracts were dried over MgSO_4_ and concentrated *in vacuo*. The crude was purified by flash column chromatography (dichloromethane/methanol, 95:5) followed by another purification step by preparative HPLC in order to separate the side product result of hydrolysis of the material. The product was obtained as a white solid (189 mg, 63%). ^1^H NMR (250 MHz, DMSO-*d*_6_) δ 10.74 (s, 1H), 8.55 (d, *J* = 5.5 Hz, 1H), 8.28 (d, *J* = 2.1 Hz, 1H), 8.14 (s, 1H), 7.77 (dd, *J* = 5.5, 2.1 Hz, 1H), 3.86 (s, 3H), 3.52 (s, 2H), 2.32 (s, 3H), 2.14 (s, 3H).

##### Methyl 5-(2-(3,5-Dimethylisoxazol-4-yl)­acetamido)­nicotinate (**56**)

2-(3,5-Dimethylisoxazol-4-yl)­acetyl chloride (120 mg, 0.691 mmol) was dissolved in dichloromethane (2.0 mL) and added dropwise to a solution of methyl 5-aminonicotinate (126 mg, 0.829 mmol) and DIPEA (182 μL, 1.04 mmol) in dichloromethane (3.0 mL). The reaction was allowed to stir overnight at rt under inert atmosphere. The reaction mixture was diluted with dichloromethane and washed with water and brine consecutively. The organic extracts were dried over MgSO_4_ and concentrated *in vacuo*. The crude was purified by preparative HPLC to obtain (65.0 mg, 33%). ^1^H NMR (250 MHz, DMSO-*d*_6_) δ 10.57 (s, 1H), 8.92 (d, *J* = 2.5 Hz, 1H), 8.77 (d, *J* = 2.0 Hz, 1H), 8.61 (t, *J* = 2.1 Hz, 1H), 3.88 (s, 3H), 3.51 (s, 2H), 2.33 (s, 3H), 2.16 (s, 3H).

##### Methyl 2-(2-(3,5-Dimethylisoxazol-4-yl)­acetamido)­isonicotinate (**57**)

(3,5-Dimethylisoxazol-4-yl)­acetic acid (150 mg, 0.97 mmol, 1.0 equiv) was dissolved in 4 mL anhydrous THF in a microwave reaction vial and methyl 2-aminoisonicotinate (152 mg, 0.97 mmol, 1.0 equiv), PyBOP (553 mg, 1.06 mmol, 1.1 equiv), HOBt·H_2_O (75 mg, 0.48 mmol, 0.5 equiv) and DIPEA (0.7 mL, 2.90 mmol, 3.0 equiv) were added sequentially. The vial was closed and heated at 60 °C for 3 h under microwave irradiation. After cooling to rt, the solvent was evaporated. The crude was taken up in DCM and washed with saturated, aqueous NaHCO_3_ solution (2×) and brine (1×). The organic phase was dried over MgSO_4_, filtered and concentrated in vacuo. The crude product was purified by column chromatography (DCM/MeOH; from 95:5 to 9:1). The product was obtained as a white solid; yield: 93 mg (0.32 mmol, 33%); ^1^H NMR (300 MHz, DMSO-*d*_6_) δ 11.01 (s, 1H), 8.56–8.55 (m, 1H), 8.52 (dd, *J* = 5.1, 0.8 Hz, 1H), 7.55 (dd, *J* = 5.1, 1.5 Hz, 1H), 3.88 (s, 3H), 3.56 (s, 2H), 2.33 (s, 3H), 2.16 (s, 3H).

##### Methyl 6-(2-(3,5-Dimethylisoxazol-4-yl)­acetamido)­picolinate (**58**)

2-(3,5-Dimethylisoxazol-4-yl)­acetyl chloride (168 mg, 0.966 mmol) was dissolved in dichloromethane (2.0 mL) and added dropwise to a solution of methyl 6-aminonicotinate (191 mg, 1.26 mmol) and DIPEA (250 μL, 1.45 mmol) in dichloromethane (3.0 mL). The reaction was allowed to stir overnight at rt under inert atmosphere. The reaction mixture was diluted with dichloromethane and washed with water and brine consecutively. The organic extracts were dried over MgSO_4_ and concentrated *in vacuo*. The crude was purified by flash chromatography (hexane/EtOAC, 1:4) to obtain (208 mg, 74%); ^1^H NMR (300 MHz, DMSO-*d*_6_) δ 11.10 (s, 1H), 8.29 (dd, *J* = 8.4, 0.9 Hz, 1H), 8.00–7.94 (m, 1H), 7.78 (dd, *J* = 7.5, 0.9 Hz, 1H), 3.88 (s, 3H), 3.57 (s, 2H), 2.33 (s, 3H), 2.16 (s, 3H)

##### 4-(2-(3,5-Dimethylisoxazol-4-yl)­acetamido)­picolinic Acid (**59**)

**55** (175 mg, 0.605 mmol) was dissolved in THF (5.0 mL) and LiOH (148 mg, 6.05 mmol) dissolved in a few μL of water was added at rt. A few μL of MeOH were added until the resulting solution was monophasic. The reaction mixture was then stirred at rt for 30 min. After this time, solvents were evaporatore under reduced pressure and the residue was purified by preparative HPLC to yield 142 mg (85%) of **59**. ^1^H NMR (250 MHz, DMSO-*d*_6_) δ 10.76 (s, 1H), 8.54 (d, *J* = 5.4 Hz, 1H), 8.27 (d, *J* = 2.0 Hz, 1H), 7.79 (dd, *J* = 5.5, 2.0 Hz, 1H), 3.58 (s, 2H), 2.33 (s, 3H), 2.15 (s, 3H).

##### 5-(2-(3,5-Dimethylisoxazol-4-yl)­acetamido)­nicotinic Acid (**60**)

**56** (62.0 mg, 0.214 mmol) was dissolved in THF (4.0 mL) and LiOH (52.4 mg, 2.14 mmol) dissolved in a few μL of water was added at rt. A few μL of MeOH were added until the resulting solution was monophasic. The reaction mixture was then stirred at rt for 30 min. After this time, solvents were evaporatore under reduced pressure and the residue was purified by preparative HPLC to yield 48.1 mg (83%) of **60**. ^1^H NMR (250 MHz, DMSO-*d*_6_) δ 13.39 (bs, 1H), 10.53 (s, 1H), 8.90 (d, *J* = 2.5 Hz, 1H), 8.75 (d, *J* = 1.8 Hz, 1H), 8.56 (t, *J* = 2.1 Hz, 1H), 3.50 (s, 2H), 2.33 (s, 3H), 2.16 (s, 3H).

##### 2-(2-(3,5-Dimethylisoxazol-4-yl)­acetamido)­isonicotinic Acid (**61**)

Synthesized according to GP2 from **57** (171 mg, 0.620 mmol) and LiOH (149 g, 6.20 mmol) in THF (5.0 mL) to yield 60.0 mg (35%) of **61**. ^1^H NMR (250 MHz, DMSO-*d*_6_) δ 10.90 (s, 1H), 8.51 (s, 1H), 8.46 (d, *J* = 5.1 Hz, 1H), 7.51 (d, *J* = 5.1 Hz, 1H), 3.56 (s, 2H), 2.33 (s, 3H), 2.16 (s, 3H).

##### 6-(2-(3,5-Dimethylisoxazol-4-yl)­acetamido)­picolinic Acid (**62**)

Synthesized according to GP2 from **58** (208 mg, 0.719 mmol) and LiOH (149 mg, 7.19 mmol) in THF (5.0 mL) to yield 93.0 mg (47%) of **62**. ^1^H NMR (300 MHz, DMSO-*d*_6_) δ 11.03 (s, 1H), 8.24 (dd, *J* = 8.3, 0.9 Hz, 1H), 7.97–7.91 (m, 1H), 7.75 (dd, *J* = 7.5, 1.0 Hz, 1H), 3.57 (s, 2H), 2.33 (s, 3H), 2.16 (s, 3H).

##### *N*-(3,4-Dichlorophenyl)-4-(2-(3,5-dimethylisoxazol-4-yl)­acetamido)­picolinamide (**63**)

Synthesized according to GP1a from **59** (142 mg, 0.516 mmol), HBTU (296 mg, 0.774 mmol), 3,4-dichloroaniline (111 mg, 0.671 mmol), 4-methylmorpholine (113 μL, 1.03 mmol) and EDCI·HCl (148 mg, 0.774 mmol) in DMF (5.0 mL). Purification by preparative HPLC yielded 39.9 mg (19%) of **63**. ^1^H NMR (300 MHz, DMSO-*d*_6_) δ 10.92 (s, 1H), 10.81 (s, 1H), 8.59 (d, *J* = 5.4 Hz, 1H), 8.31–8.29 (m, 2H), 7.94–7.90 (m, 1H), 7.60 (d, *J* = 8.7 Hz, 1H), 3.54 (s, 2H), 2.34 (s, 3H), 2.16 (s, 3H); ^13^C NMR (75 MHz, DMSO-*d*_6_) δ 170.2, 166.6, 163.3, 160.1, 150.9, 149.9, 147.7, 139.0, 131.3, 130.9, 125.8, 122.0, 120.9, 116.2, 112.4, 108.5, 30.3, 11.1,10.3. ^*t*^R HPLC: 14.1 min (13 min from 10 to 95% MeCN in water (0.1% formic acid), then 7 min 95% MeCN). 98.1% purity; HRMS (MALDI): *m*/*z* found. 419.0675 [M + H]^+^ (cal. C_19_H_17_Cl_2_N_4_O_3_^+^ 419.0672).

##### *N*-(3,4-Dichlorophenyl)-5-(2-(3,5-dimethylisoxazol-4-yl)­acetamido)­nicotinamide (**64**)

Synthesized according to GP1a from **60** (48.0 mg, 0.174 mmol), HBTU (100 mg, 0.262 mmol), 3,4-dichloroaniline (37.5 mg, 0.227 mmol), 4-methylmorpholine (38.3 μL, 0.349 mmol) and EDCI·HCl (50.1 mg, 0.262 mmol) in DMF (4.0 mL). Purification by preparative HPLC yielded 12.0 mg (16%) of **64**. ^1^H NMR (300 MHz, DMSO-*d*_6_) δ 10.69 (s, 1H), 10.58 (s, 1H), 8.93 (d, *J* = 2.5 Hz, 1H), 8.82 (d, *J* = 1.8 Hz, 1H), 8.51 (t, *J* = 2.1 Hz, 1H), 8.12 (d, *J* = 2.3 Hz, 1H), 7.73 (dd, *J* = 8.8, 2.4 Hz, 1H), 7.62 (d, *J* = 8.8 Hz, 1H), 3.52 (s, 2H), 2.35 (s, 3H), 2.17 (s, 3H); ^13^C NMR (75 MHz, DMSO-*d*_6_) δ 169.4, 166.5, 163.3, 160.1, 143.7, 143.4, 139.3, 136.0, 131.4, 131.1, 130.7, 125.9, 122.0, 120.8, 108.8, 30.0, 11.1,10.4. ^*t*^R HPLC: 12.0 min (13 min from 10 to 95% MeCN in water (0.1% formic acid), then 7 min 95% MeCN). 95.4% purity; HRMS (MALDI): *m*/*z* found. 419.0672 [M + H]^+^ (cal. C_19_H_17_Cl_2_N_4_O_3_^+^ 419.0672).

##### *N*-(3,4-Dichlorophenyl)-2-(2-(3,5-dimethylisoxazol-4-yl)­acetamido)­isonicotinamide (**65**)

Synthesized according to GP1a from **61** (57.0 mg, 0.207 mmol), HBTU (118 mg, 0.311 mmol), 3,4-dichloroaniline (41.1 mg, 0.248 mmol), 4-methylmorpholine (46.0 μL, 0.414 mmol) and EDCI·HCl (59.5 mg, 0.262 mmol) in DMF (4.0 mL). Purification by preparative HPLC yielded 24.0 mg (28%) of **65**. ^1^H NMR (400 MHz, DMSO-*d*_6_) δ 10.97 (s, 1H), 10.73 (s, 1H), 8.53 (dd, *J* = 5.1, 0.8 Hz, 1H), 8.48 (s, 1H), 8.11 (d, *J* = 2.4 Hz, 1H), 7.73 (dd, *J* = 8.9, 2.5 Hz, 1H), 7.63 (d, *J* = 8.8 Hz, 1H), 7.57 (dd, *J* = 5.1, 1.6 Hz, 1H), 3.58 (s, 2H), 2.34 (s, 3H), 2.18 (s, 3H); ^13^C NMR (101 MHz, DMSO-*d*_6_) δ 169.3, 166.0, 164.5, 159.7, 152.6, 148.7, 143.7, 138.7, 130.9, 130.7, 125.7, 121.7, 120.4, 117.3, 111.5, 108.4, 29.4, 10.6, 9.9. ^*t*^R HPLC: 15.2 min (2 min 5% MeCN in water (0.1% formic acid), then 12 min from 5 to 90% MeCN, then 7 min 90% MeCN, then 3 min from 90 to 5% MeCN, then 1 min 5% MeCN). 99.9% purity; HRMS (MALDI): *m*/*z* found. 419.0674 [M + H]^+^ (cal. C_19_H_17_Cl_2_N_4_O_3_^+^ 419.0672).

##### *N*-(3,4-Dichlorophenyl)-6-(2-(3,5-dimethylisoxazol-4-yl)­acetamido)­picolinamide (**66**)

Synthesized according to GP1a from **62** (57.0 mg, 0.207 mmol), HBTU (118 mg, 0.311 mmol), 3,4-dichloroaniline (41.1 mg, 0.248 mmol), 4-methylmorpholine (46.0 μL, 0.414 mmol) and EDCI·HCl (59.5 mg, 0.262 mmol) in DMF (4.0 mL). Purification by preparative HPLC (10 min from 50 to 90% MeCN in water (0.1% formic acid), then 5 min 90% MeCN, then 1 min from 90 to 50% MeCN, then 4 min 50% MeCN) yielded 37.0 mg (26%) of **66**. ^1^H NMR (400 MHz, DMSO-*d*_6_) δ 10.80 (s, 1H), 10.53 (s, 1H), 8.27 (d, *J* = 8.3 Hz, 1H), 8.20 (d, *J* = 2.4 Hz, 1H), 8.03 (t, *J* = 7.9 Hz, 1H), 7.80–7.76 (m, 2H), 7.66 (d, *J* = 8.8 Hz, 1H), 3.62 (s, 2H), 2.35 (s, 3H), 2.18 (s, 3H); ^13^C NMR (101 MHz, DMSO-*d*_6_) δ 169.2, 166.1, 162.8, 159.7, 150.7, 148.2, 140.2, 138.3, 131.1, 130.8, 125.5, 121.0, 119.8, 117.9, 116.8, 108.3, 29.5, 10.7, 9.9. ^*t*^R HPLC: 9.9 min (10 min from 50 to 90% MeCN in water (0.1% formic acid), then 5 min 90% MeCN, then 1 min from 90 to 50% MeCN, then 4 min 50% MeCN). 99% purity; HRMS (MALDI): *m*/*z* found. 419.0671 [M + H]^+^ (cal. C_19_H_17_Cl_2_N_4_O_3_^+^ 419.0672).

##### 2-Amino-*N*-(3,4-dichlorophenyl)­pyrimidine-4-carboxamide (**67**)

2-Aminopyrimidine-4-carboxylic acid (104 mg, 0.73 mmol, 1.0 equiv) was dissolved in thionyl chloride (1.5 mL, 20.5 mmol, 28.3 equiv), a catalytical amount of DMF was added, and the final reaction mixture was heated to reflux for 3 h. Subsequently the solvent was removed under reduced pressure and the crude was used in the next step without further purification or analytical analysis. The acid chloride (1.0 equiv, assumed) was taken up in 5 mL anhydrous DCM under an argon-Atmosphere. To this suspension 3,4-dichloroaniline (144 mg, 0.87 mmol, 1.2 equiv) dissolved in 3 mL anhydrous DCM together with DIPEA (0.2 mL, 1.09 mmol, 1.5 equiv) was added dropwise *via* a septum. The syringe was rinsed with 2 mL of DCM and the final reaction mixture was stirred overnight at rt. The mixture was then diluted with DCM and saturated, aqueous NaHCO_3_ solution and the phases were separated. The aqueous layer was extracted with DCM (1×) and EtOAc (2×) and each washed with brine. The respective combined organic phases were dried over MgSO_4_, filtered, then combined and the solvents removed under reduced pressure. The crude product was purified by flash column chromatography (hexane/EtOAc; 1:4). The product was obtained as a white solid; yield: 76 mg (37%); ^1^H NMR (300 MHz, DMSO-*d*_6_) δ 10.59 (s, 1H), 8.53 (d, *J* = 4.8 Hz, 1H), 8.19 (d, *J* = 2.4 Hz, 1H), 7.78 (dd, *J* = 8.8, 2.5 Hz, 1H), 7.63 (d, *J* = 8.8 Hz, 1H), 7.13 (d, *J* = 4.8 Hz, 1H), 6.93 (s, 2H)

##### *N*-(3,4-Dichlorophenyl)-2-(2-(3,5-dimethylisoxazol-4-yl)­acetamido)­pyrimidine-4-carboxamide (**68**)

Procedure GP1e: starting material: 2-amino-*N*-(3,4-dichlorophenyl)­pyrimidine-4-carboxamide (**67**, 0.27 mmol), reaction time: 4 days. The crude product was purified twice by flash column chromatography (1. hexane/EtOAC, 1:4; 2. hexane/acetone, 1:1), followed by a further washing step with methanol and the desired product was obtained as a white, crystalline solid; yield: 18 mg (16%). ^1^H NMR (500 MHz, DMSO-*d*_6_) δ 11.04 (s, 1H), 10.65 (s, 1H), 8.97 (d, *J* = 4.9 Hz, 1H), 8.17 (d, *J* = 2.4 Hz, 1H), 7.74–7.71 (m, 2H), 7.66 (d, *J* = 8.8 Hz, 1H), 3.75 (s, 2H), 2.33 (s, 3H), 2.15 (s, 3H); ^13^C NMR (101 MHz, DMSO-*d*_6_) δ 168.8, 165.9, 161.9, 161.3, 159.8, 157.7, 1576.0, 137.8, 131.2, 130.9, 126.1, 121.3, 120.2, 113.6, 108.3, 29.9, 10.6, 9.9. ^*t*^R HPLC: 11.0 min (2 min 5% MeCN in water (0.1% formic acid), then 12 min from 5 to 90% MeCN, then 7 min 90% MeCN, then 3 min from 90 to 5% MeCN, then 1 min 5% MeCN). 97% purity; HRMS (MALDI): *m*/*z* found. 420.0623 [M + H]^+^ (cal. C_18_H_16_Cl_2_N_5_O_3_^+^ 420.0625).

##### Methyl 5-(2-(3,5-Dimethylisoxazol-4-yl)­acetamido)­furan-2-carboxylate (**69**)

Procedure GP1b: starting material: (3,5-dimethylisoxazol-4-yl)­acetic acid (1.04 mmol), methyl-5-amino-furoat (1.35 mmol, 1.3 equiv instead of 1.2). After final purification by column chromatography (95/5, DCM/MeOH), the product was obtained as a white solid; yield: 104 mg (36%); ^1^H NMR (300 MHz, DMSO-*d*_6_) δ 11.74 (s, 1H), 7.30 (d, *J* = 3.6 Hz, 1H), 6.35 (d, *J* = 3.6 Hz, 1H), 3.78 (s, 3H), 3.47 (s, 2H), 2.31 (s, 3H), 2.13 (s, 3H).

##### Ethyl 2-(2-(3,5-Dimethylisoxazol-4-yl)­acetamido)-1*H*-imidazole-5-carboxylate (**70**)

Procedure GP1e: starting material: Ethyl 2-amino-1*H*-imidazole-5-carboxylate (0.63 mmol). Reaction time: 2.5 days. After final purification by column chromatography (95/5, DCM/MeOH), the product was obtained as a white solid; yield: 91.3 mg (50%); ^1^H NMR (250 MHz, DMSO-*d*_6_) δ 12.09 (s, 1H), 11.59 (s, 1H), 7.42 (s, 1H), 4.18 (q, *J* = 7.1 Hz, 2H), 3.47 (s, 2H), 2.33 (s, 3H), 2.16 (s, 3H), 1.25 (t, *J* = 7.1 Hz, 3H).

##### 5-(2-(3,5-Dimethylisoxazol-4-yl)­acetamido)­furan-2-carboxylic Acid (**71**)

Procedure GP2: starting material: methyl 5-(2-(3,5-dimethylisoxazol-4-yl)­acetamido)­furan-2-carboxylate (**69**, 0.37 mmol). In contrast to procedure GP2, the aqueous phase was additionally extracted with EtOAc (3×).; C_12_H_12_N_2_O_5_; MW: 264.24 g/mol; yield: 84 mg (0.32 mmol, 87%); ^1^H NMR (300 MHz, DMSO-*d*_6_) δ 11.66 (s, 1H), 7.19 (d, *J* = 3.6 Hz, 1H), 6.32 (d, *J* = 3.6 Hz, 1H), 3.46 (s, 2H), 2.32 (s, 3H), 2.14 (s, 3H).

##### 2-(2-(3,5-Dimethylisoxazol-4-yl)­acetamido)-1*H*-imidazole-5-carboxylic Acid (**72**)

Procedure GP2: starting material: Ethyl 2-(2-(3,5-dimethylisoxazol-4-yl)­acetamido)-1*H*-imidazole-5-carboxylate (**70**, 0.29 mmol). In turn, according to the procedure described in GP2, the pH of the aqueous phase was raised stepwise to pH = 6 using 1 N NaOH and extracted with EtOAc in between: yield: 51 mg (66%); ^1^H NMR (400 MHz, DMSO-*d*_6_) δ 12.02 (bs, 1H), 11.54 (bs, 1H), 7.37 (s, 1H), 3.48 (s, 2H), 2.33 (s, 3H), 2.16 (s, 3H).

##### *N*-(3,4-Dichlorophenyl)-5-(2-(3,5-dimethylisoxazol-4-yl)­acetamido)­furan-2-carboxamide (**73**)

Procedure GP1a: starting material: 5-(2-(3,5-dimethylisoxazol-4-yl)­acetamido)­furan-2-carboxylic acid (**71**, 0.30 mmol). The crude product was further purified by HPLC (10 min from 70 to 90% MeCN in water (0.1% formic acid), then 5 min 90% MeCN, then 1 min from 90 to 70% MeCN, then 4 min 70% MeCN). The product was obtained as a white solid.;C_18_H_15_Cl_2_N_3_O_4_; MW: 408.24 g/mol; yield: 20 mg (0.05 mmol, 16%). ^1^H NMR (400 MHz, DMSO-*d*_6_) δ 11.70 (s, 1H), 10.19 (s, 1H), 8.06 (d, *J* = 2.4 Hz, 1H), 7.68–7.65 (m, 1H), 7.59 (d, *J* = 8.9 Hz, 1H), 7.47 (d, *J* = 3.6 Hz, 1H), 6.39 (d, *J* = 3.6 Hz, 1H) 3.48 (s, 2H), 2.33 (s, 3H), 2.15 (s, 3H); ^13^C NMR (101 MHz, DMSO-*d*_6_) δ 166.8, 166.1, 159.6, 156.2, 149. 7, 139.0, 138.5, 130.9, 130.6, 124.8, 120.9, 119.8, 117.6, 108.1, 95.4, 28.8, 10.6, 9.8. ^*t*^R HPLC: 14.7 min (2 min 5% MeCN in water (0.1% formic acid), then 12 min from 5 to 90% MeCN, then 7 min 90% MeCN, then 3 min from 90 to 5% MeCN, then 1 min 5% MeCN). 99% purity; HRMS (MALDI): *m*/*z* found. 408.0510 [M + H]^+^ (cal. C_18_H_16_Cl_2_N_3_O_4_^+^ 408.0512).

##### *N*-(3,4-Dichlorophenyl)-2-(2-(3,5-dimethylisoxazol-4-yl)­acetamido)-1*H*-imidazole-5-carboxamide (**74**)

Procedure GP1a: starting material: 2-(2-(3,5-Dimethylisoxazol-4-yl)­acetamido)-1*H*-imidazole-5-carboxylic acid (**72**, 0.35 mmol). The crude product was purified by HPLC (10 min from 50 to 90% MeCN in water (0.1% formic acid), then 5 min 90% MeCN, then 1 min from 90 to 50% MeCN, then 4 min 50% MeCN) and obtained afterward as white solid; yield: 12 mg (16%). ^1^H NMR (500 MHz, DMSO-*d*_6_) δ 12.21 (bs, 1H), 11.39 (s, 1H), 9.80 (s, 1H), 8.17 (d, *J* = 2.4 Hz, 1H), 7.75–7.72 (m, 1H), 7.57–7.54 (m, 2H), 3.51 (s, 2H), 2.35 (s, 3H), 2.18 (s, 3H); ^13^C NMR (101 MHz, DMSO-*d*_6_) δ 168.9, 166.1, 160.9, 159.7, 140.3, 139.2, 131.8, 130.8, 130.5, 124.5, 120.8, 119.8, 118.2, 108.1, 28.6, 10.7, 9.9. ^*t*^R HPLC: 14.0 min (2 min 5% MeCN in water (0.1% formic acid), then 12 min from 5 to 90% MeCN, then 7 min 90% MeCN, then 3 min from 90 to 5% MeCN, then 1 min 5% MeCN). 99% purity; HRMS (MALDI): *m*/*z* found. 408.0624 [M + H]^+^ (cal. C_17_H_16_Cl_2_N_5_O_3_^+^ 408.0625).

##### Methyl 3-((3,4-Dichlorophenyl)­carbamoyl)­benzoate (**75**)

3-(Methoxycarbonyl)­benzoic acid (200 mg, 1.08 mmol, 1.0 equiv) was dissolved in 4 mL anhydrous THF in a microwave reaction vial and 3,4-dichloroaniline (178 mg, 1.08 mmol, 1.0 equiv), PyBOP (616 mg, 1.18 mmol, 1.1 equiv), HOBt·H_2_O (83 mg, 0.54 mmol, 0.5 equiv) and DIPEA (0.6 mL, 3.23 mmol, 3.0 equiv) were added sequentially. The vial was closed and heated at 60 °C for 3 h under microwave irradiation. After cooling to rt, the solvent was evaporated. The crude was taken up in DCM and washed with saturated, aqueous NaHCO_3_ solution (2×) and brine (1×). The organic phase was dried over MgSO_4_, filtered and concentrated in vacuo. The crude product was purified by column chromatography (hexane/EtOAc, from 9:1 to 2:1) to yield 166 mg (48%); ^1^H NMR (250 MHz, DMSO-*d*_6_) δ 10.69 (s, 1H), 8.53 (t, *J* = 1.7 Hz, 1H), 8.25–8.15 (m, 3H), 7.77 (dd, *J* = 8.8, 2.4 Hz, 1H), 7.71 (t, *J* = 7.8 Hz, 1H), 7.63 (d, *J* = 8.8 Hz, 1H), 3.91 (s, 3H).

##### 3-((3,4-Dichlorophenyl)­carbamoyl)­benzoic Acid (**76**)

Procedure GP2: starting material: methyl 3-((3,4-dichlorophenyl)­carbamoyl)­benzoate (**75**), 0.46 mmol; yield: 134 mg (0.43 mmol, 94%). ^1^H NMR (250 MHz, DMSO-*d*_6_) δ 13.27 (s, 1H), 10.68 (s, 1H), 8.53 (t, *J* = 1.8 Hz, 1H), 8.21–8.14 (m, 3H), 7.78 (dd, *J* = 8.9, 2.4 Hz, 1H), 7.72–7.61 (m, 2H).

##### *N*1-(3,4-Dichlorophenyl)-*N*3-((3,5-dimethylisoxazol-4-yl)­methyl)­isophthalamide (**77**)

Synthesized according to GP1a from **76** (129 mg, 0.416 mmol), HBTU (237 mg, 0.624 mmol), (3,5-dimethylisoxazol-4-yl)­methanamine (64.9 mg, 0.499 mmol), 4-methylmorpholine (92.4 μL, 0.832 mmol) and EDCI·HCl (120 mg, 0.624 mmol) in DMF (5.0 mL). Purification by preparative HPLC yielded 120 mg (69%) of **77**. ^1^H NMR (400 MHz, DMSO-*d*_6_) δ 10.63 (s, 1H), 8.97 (t, *J* = 5.5 Hz, 1H), 8.40 (t, *J* = 1.8 Hz, 1H), 8.15 (d, *J* = 2.4 Hz, 1H), 8.09–8.04 (m, 2H), 7.76 (dd, *J* = 8.9, 2.5 Hz, 1H), 7.66–7.61 (m, 2H), 4.24 (d, *J* = 5.4 Hz, 2H), 2.42 (s, 3H), 2.24 (s, 3H); ^13^C NMR (101 MHz, DMSO-*d*_6_) δ 166.6, 165.6, 165.4, 159.4, 139.2, 134.6, 134.5, 130.9, 130.6, 130.5, 130.4, 128.6, 126.7, 125.2, 121.4, 120.2, 111.7, 31.4, 10.7, 9.7. ^*t*^R HPLC: 8.3 min (2 min 5% MeCN in water (0.1% formic acid), then 12 min from 5 to 90% MeCN, then 7 min 90% MeCN, then 3 min from 90 to 5% MeCN, then 1 min 5% MeCN). 99% purity; HRMS (MALDI): *m*/*z* found. 418.0718 [M + H]^+^ (cal. C_20_H_18_Cl_2_N_3_O^+^ 418.0720).

##### Methyl 3-(*N*-((3,5-Dimethylisoxazol-4-yl)­methyl)­sulfamoyl)­benzoate (**78**)

(3,5-Dimethylisoxazol-4-yl)­methanamine (100 mg, 077 mmol, 1.3 equiv) and triethylamine (134 μL, 0.95 mmol, 1.6 equiv) were combined in 5 mL anhydrous toluene and the solution was degassed with Argon for several minutes. Methyl 3-(chlorosulfonyl)­benzoate (145 mg, 0.59 mmol, 1.0 equiv) dissolved in 5 mL anhydrous toluene was added dropwise and the reaction mixture was stirred for 2 h at rt under an inert atmosphere. Subsequently the solvent was concentrated in vacuo and the crude was taken up in EtOAc and washed with brine (1×). The organic phase was dried over MgSO_4_, filtered, and concentrated under reduced pressure. The product was obtained was a white/orange viscous solid; yield: 199 mg (0.55 mmol, 93%); ^1^H NMR (250 MHz, DMSO-*d*_6_) δ 8.27 (td, *J* = 1.8, 0.5 Hz, 1H), 8.22–8.15 (m, 1H), 8.01 (ddd, *J* = 7.9, 1.9, 1.2 Hz, 1H), 7.73 (td, *J* = 7.8, 0.5 Hz, 1H), 3.91 (s, 3H), 3.81 (d, *J* = 4.5 Hz, 2H), 2.20 (s, 3H), 2.03 (s, 3H).

##### 3-(*N*-((3,5-Dimethylisoxazol-4-yl)­methyl)­sulfamoyl)­benzoic Acid (**79**)

**78** (191 mg, 0.59 mmol, 1.0 equiv) was dissolved in dioxane, mixed with aqueous 1 M LiOH (33 mg, 1.35 mmol, 2.3 equiv) and stirred for 24 h at 40 °C. After cooling to rt the reaction was concentrated in vacuo. The crude was taken up in EtOAc and 2 M HCl and the aqueous layer was extracted with EtOAc (3×). The combined organic phases were dried over MgSO_4_, filtered and the solvent was removed under reduced pressure. The product was obtained as pale yellow solid; yield: 172 mg (0.55 mmol, 94%). ^1^H NMR (250 MHz, DMSO-*d*_6_) δ 13.42 (s, 1H), 8.28 (t, *J* = 1.8 Hz, 1H), 8.19–8.11 (m, 2H), 7.97 (dt, *J* = 7.8, 1.6 Hz, 1H), 7.70 (t, *J* = 7.8 Hz, 1H), 3.80 (d, *J* = 5.7 Hz, 2H), 2.20 (s, 3H), 2.04 (s, 3H).

##### *N*-(3,4-Dichlorophenyl)-3-(*N*-((3,5-dimethylisoxazol-4-yl)­methyl)­sulfamoyl)­benzamide (**80**)

Synthesized according to GP1a from **79** (167 mg, 0.538 mmol), HBTU (306 mg, 0.807 mmol), 3,4-dichloroaniline (107 mg, 0.646 mmol), 4-methylmorpholine (120 μL, 1.08 mmol) and EDCI·HCl (155 mg, 0.807 mmol) in DMF (5.0 mL). Purification by preparative HPLC yielded 25 mg (10%) of **80**. ^1^H NMR (400 MHz, DMSO-*d*_6_) δ 10.72 (s, 1H), 8.32 (t, *J* = 1.8 Hz, 1H), 8.20 (dt, *J* = 7.8, 1.4 Hz, 1H), 8.15 (d, *J* = 2.4 Hz, 1H), 7.97 (dt, *J* = 7.8, 1.3 Hz, 1H), 7.78–7.74 (m, 2H), 7.64 (d, *J* = 8.8 Hz, 1H), 3.81 (s, 2H), 2.21 (s, 3H), 2.06 (s, 3H); ^13^C NMR (101 MHz, DMSO-*d*_6_) δ 166.7, 164.4, 159.1, 141.2, 139.0, 135.1, 131.4, 131.0, 130.7, 129.6, 129.5, 125.8, 125.5, 121.7, 120.5, 110.1, 34.6, 10.5, 9.4. ^*t*^R HPLC: 15.7 min (2 min 5% MeCN in water (0.1% formic acid), then 12 min from 5 to 90% MeCN, then 7 min 90% MeCN, then 3 min from 90 to 5% MeCN, then 1 min 5% MeCN). 97% purity; HRMS (MALDI): *m*/*z* found. 454.0386 [M + H]^+^ (cal. C_19_H_18_Cl_2_N_3_O_4_S^+^ 454.0390).

##### *tert*-Butyl (3-((3,4-Dichlorophenyl)­carbamoyl)­phenyl)­carbamate (**81**)

Procedure GP1b: starting material: 3-((*tert*-butoxycarbonyl)­amino)­benzoic acid (4.51 mmol), 3,4-dichloroaniline (4.96 mmol), 1.1 equiv instead of 1.2. After purification by column chromatography (hexane/EtOAc, from 2:1 to 1:1); yield: 751 mg (44%); ^1^H NMR (250 MHz, DMSO-*d*_6_) δ 10.48 (s, 1H), 9.57 (s, 1H), 8.14 (d, *J* = 2.4 Hz, 1H), 8.04 (t, *J* = 1.9 Hz, 1H), 7.75 (dd, *J* = 8.9, 2.4 Hz, 1H), 7.64–7.59 (m, 2H), 7.55–7.51 (m, 1H), 7.41 (t, *J* = 7.8 Hz, 1H), 1.49 (s, 9H).

##### 3-Amino-*N*-(3,4-dichlorophenyl)­benzamide (**82**)

Boc-protected amine **81** (750 mg, 1.97 mmol) was dissolved in anhydrous DCM (50 mL) followed by the addition of trifluoracetic acid (50 equiv) and the reaction was stirred for 3 h at rt. The solvent was evaporated and the slurry was taken up in EtOAc and saturated, aqueous sodium bicarbonate solution to adjust pH = 8. The aqueous phase was extracted with EtOAc (6×). The combined organic phases were dried over MgSO_4_, filtered and the solvent was removed under reduced pressure. The obtained solid was used without further purification.; yield: 555 mg (1.96 mmol, 99%); ^1^H NMR (250 MHz, DMSO-*d*_6_) δ 10.33 (s, 1H), 8.14 (d, *J* = 2.4 Hz, 1H), 7.74 (dd, *J* = 8.9, 2.4 Hz, 1H), 7.59 (d, *J* = 8.8 Hz, 1H), 7.16 (t, *J* = 7.7 Hz, 1H), 7.09–7.04 (m, 2H), 6.77 (ddd, *J* = 7.9, 2.3, 1.1 Hz, 1H) 5.40 (s, 2H).

##### *N*-(3,4-Dichlorophenyl)-3-((2-(3,5-dimethylisoxazol-4-yl)­ethyl)­amino)­benzamide (**83**)

To a solution of the **82** (70 mg, 0.25 mmol) in 5 mL anhydrous DMF at rt, NaH (60%, 30.4 mg, 0.75 mmol) was added and the solution was degassed with argon for a few minutes. Then 4-(2-bromoethyl)-3,5-dimethylisoxazole (64 mg, 0.30 mmol) was added dropwise and the reaction stirred overnight under argon atmosphere. The reaction was stopped by adding methanol and the solvents were removed under reduced pressure. The crude product was suspended in EtOAc and was washed with brine (3×). The organic phase was dried over MgSO_4_, filtered and the solvent was removed under reduced pressure. The crude product was purified two times by HPLC (1. Two minutes 5% MeCN in water (0.1% formic acid), then 12 min from 5 to 90% MeCN, then 7 min 90% MeCN, then 3 min from 90 to 5% MeCN, then 1 min 5% MeCN and 2. Ten minutes from 50 to 90% MeCN in water (0.1% formic acid), then 5 min 90% MeCN, then 1 min from 90 to 50% MeCN, then 4 min 50% MeCN) and the desired product was obtained as solid; yield: 7 mg (7%). ^1^H NMR (400 MHz, DMSO-*d*_6_) δ 10.34 (s, 1H), 8.14 (d, *J* = 2.4 Hz, 1H), 7.75 (dd, *J* = 8.9, 2.5 Hz, 1H), 7.60 (d, *J* = 8.8 Hz, 1H), 7.23 (t, *J* = 7.8 Hz, 1H), 7.11–7.08 (m, 2H), 6.80 (ddd, *J* = 8.1, 2.4, 1.0 Hz, 1H), 5.99 (t, *J* = 6.0 Hz, 1H), 3.20 (q, *J* = 6.8 Hz, 2H), 2.56 (t, *J* = 7.1 Hz, 2H), 2.25 (s, 3H), 2.16 (s, 3H); ^13^C NMR (101 MHz, DMSO-*d*_6_) δ 166.6, 165.3, 159.4, 148.6, 139.5, 135.3, 130.8, 130.5, 129.0, 124.9, 121.3, 120.2, 115.6, 114.8, 111.4, 110.7, 42.4, 21.2, 10.6, 9.8. ^*t*^R HPLC: 16.7 min (2 min 5% MeCN in water (0.1% formic acid), then 12 min from 5 to 90% MeCN, then 7 min 90% MeCN, then 3 min from 90 to 5% MeCN, then 1 min 5% MeCN). 99% purity; HRMS (MALDI): *m*/*z* found. 404.0926 [M + H]^+^ (cal. C_20_H_20_Cl_2_N_3_O_2_^+^ 404.0927).

##### *N*-(3,4-Dichlorophenyl)-3-((N-(3,5-dimethylisoxazol-4-yl)­sulfamoyl)­amino)­benzamide (**84**)

To a suspension of (3,5-dimethylisoxazol-4-yl)­sulfamoyl chloride (**82**, 246 mg, 1.17 mmol) in 5 mL anhydrous DCM was added dropwise a solution of (**84**, 164 mg, 0.59 mmol) in 15 mL DCM. Triethylamine (165 μL, 1.17 mmol) was subsequently added and the now clear reaction solution was stirred overnight at rt. The solvent was removed under reduced pressure and the crude product was purified two times by prepartative HPLC (10 min from 70 to 90% MeCN in water (0.1% formic acid), then 5 min 90% MeCN, then 1 min from 90 to 70% MeCN, then 4 min 70% MeCN) and the desired product was obtained as a colorless, crystalline solid; yield: 31 mg (0.07 mmol, 12%). ^1^H NMR (400 MHz, DMSO-*d*_6_) δ 10.52 (s, 1H), 10.28 (bs, 1H), 9.48 (bs, 1H), 8.14 (d, *J* = 2.4 Hz, 1H), 7.76–7.72 (m, 2H), 7.63–7.60 (m, 2H), 7.50 (t, *J* = 7.9 Hz, 1H), 7.42–7.39 (m, 1H), 2.04 (s, 3H), 1.97 (s, 3H); ^13^C NMR (101 MHz, DMSO-*d*_6_) δ 165.9, 165.7, 158.7, 139.2, 139.0, 135.5, 130.9, 130.6, 129.2, 125.2, 121.4, 121.3, 120.5, 120.3, 116.8, 112.9, 10.4, 9.0. ^*t*^R HPLC: 15.4 min (2 min 5% MeCN in water (0.1% formic acid), then 12 min from 5 to 90% MeCN, then 7 min 90% MeCN, then 3 min from 90 to 5% MeCN, then 1 min 5% MeCN). 99% purity; HRMS (MALDI): *m*/*z* found. 455.0337 [M + H]^+^ (cal. C_18_H_17_Cl_2_N_4_O_4_S^+^ 455.0342).

##### *N*-(3,4-Dichlorophenyl)-3-hydroxybenzamide (**85**)

3-Hydroxybenzoic acid (302 mg, 2.19 mmol) was dissolved in 6 mL toluene, followed by the addition of 3,4-dichloroaniline (470 mg, 2.84 mmol) and triphenyl phosphite (0.80 mL, 2.84 mmol). The resulting mixture was refluxed overnight under inert atmosphere. After cooling to rt the solid formed was separated by filtration and washed with cooled toluene. The filtrate was concentrated and placed in the fridge overnight and again filtrated afterward. Both fraction were combined and further purified by column chromatography (hexane/EtOAc, 3:1) yielding the desired product as white, solid; yield: 90 mg (15%); ^1^H NMR (300 MHz, DMSO-*d*_6_) δ 10.41 (s, 1H), 9.79 (s, 1H), 8.15 (d, *J* = 2.4 Hz, 1H), 7.75 (dd, *J* = 8.9, 2.5 Hz, 1H), 7.60 (d, *J* = 8.8 Hz, 1H), 7.40–7.30 (m, 3H), 7.02–6.97 (m, 1H).

##### *N*-(3,4-Dichlorophenyl)-3-(2-(3,5-dimethylisoxazol-4-yl)­ethoxy)­benzamide (**86**)

*N*-(3,4-dichlorophenyl)-3-hydroxybenzamide (**85**, 90 mg, 0.32 mmol) was placed in 15.0 mL acetone and admixed with 4-(2-bromoethyl)-3,5-dimethylisoxazole (72 mg, 0.36 mmol), tetrabutylammonium iodide (240 mg, 0.64 mmol) and potassium carbonate (132 mg, 0.96 mmol). The resulting mixture was refluxed for 4 h. After cooling to rt the solvent was removed under reduced pressure and the crude was taken up in EtOAc. The organic phase was washed with H_2_O (1×), brine (1×), dried over MgSO_4_, filtered and concentrated under reduced pressure. The crude product was further purified by HPLC (10 min from 70 to 90% MeCN in water (0.1% formic acid), then 5 min 90% MeCN, then 1 min from 90 to 70% MeCN, then 4 min 70% MeCN) and the desired product was obtained as solid; yield: 25 mg (19%). ^1^H NMR (400 MHz, DMSO-*d*_6_) δ 10.44 (s, 1H), 8.14 (d, *J* = 2.4 Hz, 1H), 7.75 (dd, *J* = 8.8, 2.5 Hz, 1H), 7.61 (d, *J* = 8.8 Hz, 1H), 7.52 (dt, *J* = 7.8, 1.2 Hz, 1H), 7.46–7.42 (m, 2H), 7.16 (dd, *J* = 8.1, 1.6 Hz, 1H), 4.12 (t, *J* = 6.5 Hz, 2H), 2.81 (d, *J* = 6.4 Hz, 2H), 2.36 (s, 3H), 2.23 (s, 3H); ^13^C NMR (101 MHz, DMSO-*d*_6_) δ 165.7, 165.5, 159.6, 158.2, 139.2, 135.7, 130.8, 130.5, 129.7, 125.1, 121.5, 120.3, 120.0, 118.0, 113.4, 110.4, 67.0, 21.6, 10.7, 9.8. ^*t*^R HPLC: 11.1 min (10 min from 50 to 90% MeCN in water (0.1% formic acid), then 5 min 90% MeCN, then 1 min from 90 to 50% MeCN, then 4 min 50% MeCN). 98% purity; HRMS (MALDI): *m*/*z* found. 405.0766 [M + H]^+^ (cal. C_20_H_19_Cl_2_N_2_O_3_^+^ 405.0767).

##### *N*-(2-Amino-4,5-dichlorophenyl)-3-(2-(3,5-dimethylisoxazol-4-yl)­acetamido)­benzamide (**87**)

4,5-Dichloro-1,2-phenylenediamine (78.3 mg, 0.438 mmol), **30** (120 mg, 0.438 mmol) and HATU (216 mg, 0.569 mmol) were dissolved in 5 mL anhydrous DMF. DIPEA (100 μL, 0.56 mmol) was added dropwise and the reaction mixture was stirred overnight at rt. Water was added to the reaction mixture, and the precipitate was filtered and dried *in vacuo*. The precipitate was purified by flash chromatography to obtain the product as a brown solid (103 mg, 55%). ^1^H NMR (300 MHz, DMSO-*d*_6_) δ 10.32 (s, 1H), 9.67 (s, 1H), 8.09 (s, 1H), 7.83 (d, *J* = 7.8 Hz, 1H), 7.67 (d, *J* = 7.5 Hz, 1H), 7.44 (t, *J* = 7.8 Hz, 2H), 6.97 (s, 1H), 5.38 (s, 2H), 3.46 (s, 2H), 2.34 (s, 3H), 2.17 (s. 3H).

##### *N*-(3-(5,6-Dichloro-1*H*-benzo­[*d*]­imidazol-2-yl)­phenyl)-2-(3,5-dimethylisoxazol-4-yl)­acetamide (**88**)

**87** (103 mg, 0.238 mmol) was dissolved in 3 mL acetic acid and refluxed at 130 °C for 3 h. After this time, the reaction was fully conversed and acetic acid was evaporated under reduced pressure. The residue was purified by preparative HPLC to yield 64.1 mg (65%) of **88**. ^1^H NMR (300 MHz, DMSO-*d*_6_) δ 13.23 (s, 1H), 10.35 (s, 1H), 8.5s (s, 1H), 7.82 (dd, *J* = 7.8 Hz, 1H), 7.67 (d, *J* = 8.0 Hz, 1H), 7.50 (t, *J* = 7.9 Hz, 1H), 3.48 (s, 2H), 2.35 (s, 3H), 2.19 (s, 3H); ^13^C NMR (75 MHz, DMSO-*d*_6_) δ 168.8, 166.4, 160.1, 154.1, 140.1, 130.2, 129.9, 124.9, 122.0, 121.7, 118.2, 109.1, 30.2, 11.1, 10.4; ^*t*^R HPLC: 12.3 min (13 min from 10 to 95% MeCN in water (0.1% formic acid), then 7 min 95% MeCN). 97.1% purity; HRMS (MALDI): *m*/*z* found. 415.0726 [M + H]^+^ (cal. C_19_H_17_Cl_2_N_4_O_2_^+^ 415.0723).

### Cell Culture

HepG2 cells were maintained at 5% CO_2_ and 37 °C in high glucose DMEM with phenol red and l-glutamine (Gibco, #41965) supplemented with 20% FBS, penicillin (100 U/mL), streptomycin (100 μg/mL), and 1 mM pyruvate.

Prior to using the cells in the CellTiter-Glo assay, the tissue culture (TC) flask was coated with collagen G. To do this, 10 mL of PBS containing 0.01 mg/mL collagen G (Merck, #L7213) was incubated for 30 min at 37 °C in a new 175 cm^2^ TC flask, which was then immediately replaced with fresh growth medium.

CHO-K1 cells (DSMZ, ACC 110) were maintained in Ham’s F-12 medium (ThermoFisher) supplemented with 10% fetal bovine serum (FBS) and 100 units/ml Penicillin and 100 μg/mL Streptomycin (Gibco) in the following referred to as full growth medium.

### Stable CHO-K1 Cell Lines for IP-One Experiments

The CHO-K1 derived cell lines used for IP-One experiments were previously generated using the sleeping beauty method.^2,19^ In brief, the coding sequences for G2A (uniport Q9UNW8–1) and GNA11 (uniport P29992) were cloned into plasmids conferring Blasticidin and Puromycin resistance, respectively. Stable integration was achieved *via* cotransfection with the transposase-expressing plasmid. Expression is driven by the constitutively active EF1a promoter. The single-cell clone coexpressing G2A and GNA11, previously characterized and utilized in our recent G2A agonist high-throughput screening campaign,^2^ was employed for all experiments presented in this study. A control cell line stably transfected with only GNA11 was included to assess potential target-independent cellular responses. No such responses were observed for any of the compounds investigated (data not shown).

### IP-One Assay for G2A

Activation of human G2A was assessed in a functional cell-based assay that measured the accumulation of IP-1 (inositol monophosphate) as a downstream effect of G2A signaling. IP-1 produced by the cells is detected in a tracer displacement system based on homogeneous time-resolved FRET (HTRF) between the FRET acceptor-coupled IP-1 and Terbium cryptate-coupled anti-IP-1 antibody (IP-One assay kit, cisbio, part of PerkinElmer). CHO-K1 cells stably coexpressing human G2A and GNA11, along with control cells expressing only GNA11, were seeded at 12,500 cells/well in white 384-well TC plates (Greiner Bio-One) and incubated overnight at 37 °C with 5% CO_2_. Following medium removal with a Tecan HydroSpeed plate washer, cells were washed four times with stimulation buffer [146 mM NaCl, 4.2 mM KCl, 1 mM CaC_l2_, 0.5 mM MgC_l2_, 50 mM LiC_l2_, 5.5 mM d-glucose, 0.1%(w/v) fatty acid free bovine serum albumin fraction V (Carl Roth, Karlsruhe, Germany) buffered with 10 mM HEPES at pH 7.4 (NaOH)].

Subsequently, the synthesized compounds and 0.5% DMSO were added, and the plate was sealed and incubated at 37 °C for 90 min. Cells were then lysed with detection agents prepared in lysis buffer according to the manufacturer’s instructions. Plates were stored at room temperature overnight before HTRF measurement on a Tecan Spark equipped with an enhanced fluorescence module. In a filter-based method, fluorescence intensity (FI) at 665 nm (d2, FRET acceptor) and at 620 nm (Terbium cryptate, FRET donor) was recorded following excitation at 340 nm and a 100 μs delay. The HTRF signal was calculated by multiplying FI665 by 10,000 and dividing by FI620. The concentration of IP-1 was determined from a standard curve of unlabeled IP-1 dilutions. Experiments included three technical replicates (*N* = 3), and mean IP-1 values with standard error were calculated in Excel and analyzed in GraphPad Prism 10.2.3 using four-parameter curve fitting (variable hill slope). Dose–response experiments assessing G2A activation with the reference agonist ± 9-HODE (Cayman Chemicals, #38400) were conducted in parallel to ensure assay performance.

### PRESTO-Tango Assay as GPCR Off-Target Screen

White clear bottom 384-well plates from PerkinElmer (cat-no: 6007480) were coated with poly-l-Lysin, and HTLA cells were seeded at 5000 cells/well. After 6 h, the cells were transfected with a plasmid from the PRESTO-Tango kit (Addgene, cat-no: Kit #1000000068) using a protocol based on Kroeze et al.^20^ For each well, a mixture containing 10 ng of plasmid and 0.04 μL of Lipofectamine 2000 was used. GFP served as the transfection control, while 100 μM carbachol at the muscarinic M5 receptor was employed as an assay control. After 24 h, the medium was removed and replaced with 45 μL of serum-free medium. Subsequently, 5 μL of ligand was added, achieving a final concentration of 10 μM, and incubated for approximately 24 h. Following this, the medium was aspirated, and the cells were lysed with 50 μL of a diluted bright-Glo reagent (Promega cat-no: E2610) in PBS (10× dilution). After a 15 min incubation with the lysis buffer, luminescence was measured using a Flexstation 3 (Molecular Devices) with an integration time of 1500 ms.

### Cell Toxicity

Cell viability was assessed using CellTiter-Glo (Promega) following the manufacturer’s instructions with slight modifications. Hep-G2 cells were harvested from collagen G-coated TC flasks using trypsin and resuspended in white DMEM high-glucose medium (Gibco #31053) supplemented with 10% FBS, penicillin (100 U/mL), streptomycin (100 μg/mL), 1 mM pyruvate, and 2 mM l-glutamine. The cell suspension was filtered through a 40 μm cell strainer (PluriSelect #43–50040) to eliminate cell clumps, and the cell density was adjusted to 100,000 cells/mL.

Next, 30 μL of this suspension, corresponding to 3,000 cells, were seeded into 96-well half a rea white polystyrene flat-bottom TC plates (Greiner Bio-One, #675083). Compounds were added in 10 μL of medium containing 2% DMSO to achieve the desired concentrations, resulting in a final DMSO concentration of 0.5% during treatment at 37 °C with 5% CO_2_.

For detection, 20 μL of CellTiter-Glo reagent mix was added per well, protected from light, and allowed to incubate for 30 min at room temperature before luminescence was measured using the standard attenuation protocol on a Tecan SPARK. Wells containing medium only served as the 0% cell viability control, while wells with cells treated solely with DMSO acted as the 100% viability control. Additionally, treatment with 50 μM paclitaxel was performed in parallel as a control for assay performance.

### Aqueous Solubility

Aqueous solubility was evaluated as previously described.^21^ Final concentrations of 2, 5, 8, 12, 17, 26, 39, 59, 88, 132, 198, 296, 444, 667, and 1000 μM of **31** and **65** were prepared in PBS pH 7.4 solution containing 1% DMSO, in a 96-well transparent flat bottom microtiterplate. Precipitation of the compound was measured at 600 and 800 nm after 1 and 24 h at room temperature using a microplate reader (Infinite M200, Tecan Group Ltd., Crailsheim, Germany) and solution clarity was confirmed by eye.

### Metabolic Stability

Compounds were tested according to the following method: A solution of compound (final concentration 1 mM) was prepared in 100% DMSO. 432 μL phosphate buffer (0.1 M, pH 7.4) together with 50 μL NADPH-regenerating system (30 mM glucose-6-phosphate, 4 U/ml glucose-6-phosphate dehydrogenase, 10 mM NADP, 30 mM MgCl2) and 5 μL of the corresponding test compound were preincubated at 37 °C. After 5 min the reaction was started by the addition of 13 μL microsome mix from the liver of Sprague–Dawley rats (Thermo Fisher Scientific, Darmstadt, Germany; 20 mg protein/ml in 0.1 M phosphate buffer). The incubation was performed in a shaking water bath at 37 °C. The reaction was stopped by the addition of 500 μL ice-cold methanol at 0, 30, and 60 min. The samples were centrifuged at 5000*g* for 5 min at 4 °C. The supernatants were analyzed and quantified by HPLC. Control samples were always performed to check the stability of the compounds in the reaction mixture. The first control was without NADPH, which is needed for the enzymatic activity of the microsomes. The second control was with inactivated (microsomes which had been incubated for 20 min at 90 °C). The third control was without test compounds (to determine the baseline). The amounts of the test compounds were quantified by an external calibration curve.

### DSF-Based Selectivity Screening against a BET Bromodomains

The assay was performed as previously described.^17^ Briefly, recombinant BET bromodomains at a concentration of 5 μM were mixed with 20 μM compound **31**, **65**, or **JQ1** in a buffer containing 20 mM HEPES, pH 7.5, and 500 mM NaCl. SYPRO Orange (5000×, Invitrogen) was added as a fluorescence probe (1 μL per mL). Subsequently, temperature-dependent protein unfolding profiles were measured using the QuantStudio 5 realtime PCR machine (Thermo Fisher). Excitation and emission filters were set to 465 and 590 nm, respectively. The temperature was raised with a step rate of 3 °C per min. Data points were analyzed with the internal software (Thermal Shift Software Version 1.4, Thermo Fisher) using the Boltzmann equation to determine the inflection point of the transition curve.

### Isolation of Peripheral Sensory Neurons

Peripheral sensory neurons were isolated from the dorsal root ganglia of mice as described before.^7^ Briefly, male C57Bl/6NRj mice, aged 8 to 12 weeks, were purchased from the commercial breeding company Janvier (Le Genest-Saint-Isle, France). Animals were housed at a 12-h day/night cycle with food and water available ad libitum. For tissue isolation, mice were euthanized with CO_2_ at a flow rate of 0.4 for 1 min and 0.8 for 3 min and a subsequent heart puncture. The dorsal root ganglia were extracted and cultured overnight for calcium imaging experiments.

### Calcium Imaging

In order to observe changes in intracellular calcium levels, sensory neurons from mice were stained with Fura-2-AM (Biotium) for 60 min at 37 °C and washed afterward twice with freshly prepared Ringer’s solution (145 mM NaCl, 1.25 mM CaCl_2_ × 2H_2_O, 1 mM MgCl_2_ × 6 H_2_O, 5 mM KCl, 10 mM d-glucose and 10 mM HEPES dissolved in purified water, pH adjusted to 7.3). During the experiment, cells were exposed to a constant flow of Ringer’s solution (1–2 mL/min) for baseline measurements and in between the application of compounds to wash out any stimulants and allow the cells to recover. To observe the compounds’ ability to modulate the 9-HODE-mediated TRPV1 sensitization, sensory neurons were stimulated twice with capsaicin (50 nM) for 30 s and preincubated with 9-HODE or 9-HODE together with the tested compounds for 4 min before the second capsaicin stimulus. At the end of each experiment, cells were stimulated with KCl (50 mM) for 1 min as a positive control to depolarize neurons. Control experiments were conducted with each respective volume of corresponding vehicle. All compounds were dissolved in Ringer’s solution. Changes in intracellular calcium concentrations were observed with a DMI4000 B Microscope, a compact light source CTR550 HS (Leica) and a ValveBank II perfusion system (AutoMate Scientific). The cells were observed at 340 and 380 nm excitatory wavelengths while the emission was detected at 510 nm. Changes in intracellular calcium concentrations were determined as Δratio of the fluorescence intensities F340/F380.

### Pharmacokinetic Study

The pharmacokinetics studies were performed by CRO Enamine/Bienta (Kiyv, Ukraine). All animal experiments were conducted in accordance with local regulations and approved by the BACUC, approval number #GUF-PK-26022024. The studies were performed in 8 weeks old male CD1 mice (weight range 24.1 g–33.7 g). Compound **65** was dissolved in DMSO–Kolliphor HS 15—Water for injections (10%:20%:70%, v/v/v) and applied i.p. (10 mg/kg). All animals showed a normal behavior and there were no clinical signs observed after dosing. Study design, animal selection, handling and treatment were all in accordance with the Enamine PK study protocols and Institutional Animal Care and Use Guidelines. The plasma concentration was determined *via* LC–MS. Experimental details are described in the Supporting Information.

## Supplementary Material





## Notes

Synthesis methods and characterization of intermediate compounds and reference antagonistsHPLC traces of selected compoundsNMR spectra of selected compoundsMaterials and methods for the mouse PK experimentDose-dependent activation of FPR3Dose–response results of reference compounds in the IP-One assay (PDF)
Molecular formula strings (CSV)

## Abbreviations Used

9-HODE: 9-Hydroxyoctadecadienoic acid
BTFFH: fluor-*N*,*N*,*N*′,*N*′-bis­(tetramethylen)­formamidiniumhexafluorphosphat
FPR3: formyl peptide receptor 3
CHO: chinese hamster ovary
EFα: elongation factor α
G2A: G protein coupled receptor 132
GNA11: G alpha subunit 11
IP-1: inositol monophosphate
IPA: invasive pulmonary aspergillosis
LPC: lysophsophatidylcholine
MW: microwave
OINP: oxaliplatin-induced neuropathic pain
RLU: relative light unit
TRPV1: transient receptor potential cation channel subfamily V member 1
