# Supplementary material for: First Structure–Activity-Relationship Study of Potent G2A Antagonists
Source: J Med Chem. 2026 Jun 11;69(12):14309–32. doi: 10.1021/acs.jmedchem.6c00079 (PMC13312408; doi:10.1021/acs.jmedchem.6c00079)
Supplement: Supplementary file 1 [file jm6c00079_si_001.pdf]

Supporting Information

First Structure-Activity-Relationship study of potent G2A Antagonists

Victor Hernandez-Olmos[a,b], Jan Heering[a,b], Felix F. Lillich[c], Beatrice Marinescu[c], Sheila Nevermann[a,d], Johanna H. M. Ehrler[c], Dmytro S. Radchenko[e], Yurii S. Moroz[e,g,f], Astrid Kaiser[c], Andreas Krämer[c], Stefan Knapp[c], Manfred Schubert-Zsilavecz[c], Mohamad Wessam Alnouri[h], Stefan Offermanns[h,i], Dieter Steinhilber[a,b,c], Marco Sisignano,[a,b,d] and Ewgenij Proschak[a,b,c]*

[a] Fraunhofer Institute for Translational Medicine and Pharmacology ITMP, Theodor-Stern-Kai 7, 60596 Frankfurt am Main

[b] Fraunhofer Cluster of Excellence Immune-Mediated Diseases CIMD, 60596 Frankfurt am Main, Germany

[c] Institute of Pharmaceutical Chemistry, Goethe University Frankfurt, Max-von-Laue-Str. 9, 60438 Frankfurt am Main, Germany

[d] Institute of Clinical Pharmacology, Pharmazentrum Frankfurt/ZAFES, Goethe-University, D-60590 Frankfurt am Main, Germany

[e] Enamine Ltd. 78 Winston Churchill Street, Kyiv, 02094, Ukraine

[f] Chemspace LLC, 85 Winston Churchill Street, Suite 1, Kyiv, 02094, Ukraine

[g] National Taras Shevchenko University of Kyiv, 60 Volodymyrska Street, Kyiv 01601, Ukraine

[h] Department of Pharmacology, Max Planck Institute for Heart and Lung Research, 61231 Bad Nauheim, Germany

[i] Center for Molecular Medicine, Goethe University Frankfurt, 60590 Frankfurt, Germany

* proschak@pharmchem.uni-frankfurt.de

Syntesis of intermediate 2-(3,5-Dimethylisoxazol-4-yl)acetyl chloride S3

Synthesis of SB-583355 S3

Synthesis of NOX-6-18 S5

SI Figure S1: HPLC trace of compound **31**. S7

SI Figure S2: HPLC trace of compound **32** S7

SI Figure S3: HPLC trace of compound **34** S8

SI Figure S4: HPLC trace of compound **65.** S8

SI Figure S5: ^{1}H NMR Spectrum of compound **31.** S9

SI Figure S6: ^{13}C NMR Spectrum of compound **31.** S9

SI Figure S7: ^{1}H NMR Spectrum of compound **65.** S10

SI Figure S8: ^{13}C NMR Spectrum of compound **65.** S10

Materials and methods for the mouse PK experiment with compound **65** (FL319) S11

SI Table S1: Study design. S12

SI Table S2: Other MS parameters. S13

SI Figure S9: Method validation results S14

SI Figure S10: Calibration curve S15

SI Figure S11: Dose-dependent activation of FPR3 by compounds 31 and 65 S16

SI Figure S12: Dose-dependent response of reference compounds **NOX-6-18 and SB-583355** S16

2-(3,5-Dimethylisoxazol-4-yl)acetyl chloride

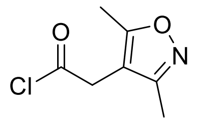

(3,5-Dimethylisoxazol-4-yl)acetic acid (160 mg, 1.03 mmol) was dissolved in dichloromethane (4.0 mL) thionyl chloride (760 µL, 10.3 mmol) was added. The reaction mixture was refluxed for 3h. The solvent was evaporated and the crude product was used in the next step without further purification.

__Synthesis of SB-583355__

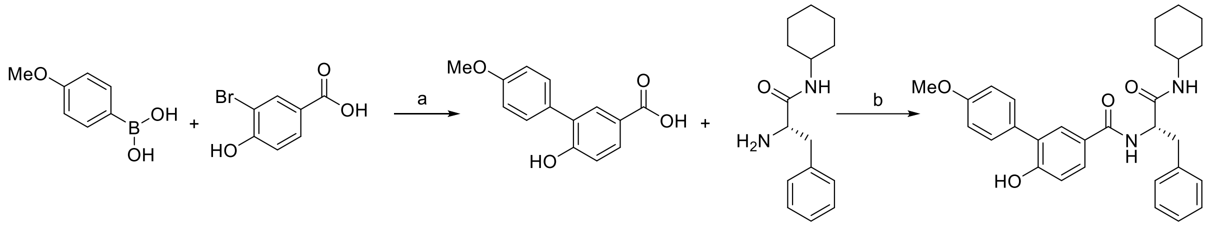

SB-583355

Scheme S1. Synthesis of **SB-583355**. a) $Pd(OAc)_2$, Cs_2CO_3, DMF, H_2O, 45°C, 83% b) HBTU, EDCl, 4-methylmorpholine, DMF, rt, 20%.

6-Hydroxy-4'-methoxy-[1,1'-biphenyl]-3-carboxylic acid

A mixture of 3-bromo-4-hydroxbenzoic acid (1.0 mmol, 217 mg), 4-methoxyphenylboronic acid (1.0 mmol, 157 mg), cesium carbonate (3.0 mmol, 977 mg) and palladium acetate (0.030 mmol, 6.74 mg) was dissolved in DMF (5.0 mL) and water (2.0 mL) under Ar. The resulting solution was

heated at 45°C overnight. After this time, the reaction mixture was diluted with water and the pH adjusted to 3 with an aqueous 1M HCl solution. The aqueous phase was extracted with ethyl acetate (3x). The combined organic layers were dried over magnesium sulfate, filtered and evaporated. The residue was purified by flash chromatography (cyclohexane/ethyl acetate, 8:2 to 4:6) to yield 202 mg (83%) of pure compound. ^{1}H NMR (400 MHz, DMSO-d_6) δ 12.49 (br s, 1H), 10.34 (s, 1H), 7.80 (d, *J* = 2.2 Hz, 1H), 7.73 (dd, *J* = 8.4, 2.2 Hz, 1H), 7.48 (d, *J* = 8.8 Hz, 2H), 6.99 (d, *J* = 8.4 Hz, 1H), 6.98 (d, *J* = 8.8 Hz, 2H), 3.79 (s, 3H).

(*S*)-*N*-(1-(Cyclohexylamino)-1-oxo-3-phenylpropan-2-yl)-6-hydroxy-4'-methoxy-[1,1'-biphenyl]-3-carboxamide (SB-583355)

A mixture of 6-hydroxy-4'-methoxy-[1,1'-biphenyl]-3-carboxylic acid (0.409 mmol, 100 mg) and HBTU (0.614 mmol, 118 mg) were dissolved in DMF (5.0 mL) under Ar and stirred at rt for 15 min. After this time, L-phenylalanine cyclohexylamide (0.45 mmol, 111 mg) and 4-methylmorpholine (2.45 mmol, 269 µL) were added at 0°C and the reaction mixture was stirred at this temperature for 30 min. EDCl (0.614 mmol, 118 mg) was then added at 0°C and the reaction mixture was stirred at rt overnight. After this time, the reaction mixture was evaporated under reduced pressure, and the residue was redissolved in water and ethyl acetate. The aqueous phase was extracted with ethyl acetate (3x). The combined organic layers were dried over magnesium sulfate, filtered and evaporated. The residue was purified by preparative HPLC to yield 38.9 mg (20%) of pure compound. ^{1}H NMR (500 MHz, $CDCl_3$) δ 7.79 (br s, 1H), 7.66 (s, 1H), 7.50 (d, *J* = 8.0 Hz, 1H), 7.39 (d, *J* = 8.6 Hz, 2H), 7.25-7.17 (m, 5H), 7.09 (d, *J* = 4.9 Hz, 1H), 6.94-6.91 (m, 3H), 6.17 (br s, 1H), 4.84 (q, *J* = 6.1 Hz, 1H), 3.81 (s, 3H), 3.65-3.67 (m, 1H), 3.20 (dd, *J* = 13.3, 5.6 Hz, 1H), 3.07 (dd, *J* = 12.9, 8.4 Hz, 1H), 1.71 (d, *J* = 10.2 Hz, 1H), 1.61 (d, *J* = 11.5 Hz, 1H), 1.56-1.48 (m, 3H), 1.26-1.19 (m, 2H), 1.03-0.90 (m, 2H), 0.84 (q, *J* = 11.2 Hz, 1H). ^{13}C NMR (125 MHz, $CDCl_3$) δ 170.3, 166.9, 159.2, 156.6, 136.7, 130.3, 129.9, 129.4, 129.0, 128.6, 128.2, 127.5, 126.9, 125.5, 115.9, 114.2, 55.3, 48.4, 39.1, 32.6, 32.4, 25.2, 24.6; tR HPLC: 12.8 Min (13 Min from 10 to 95% MeCN in

water (0.1 % formic acid), then 7 min 95% MeCN). 99.7 % purity; HRMS (MALDI): m/z found. 495.2261 $[M+Na]^+$ (cal. $C_{29}H_{32}N_2O_4Na^+$ 495.2254).

Synthesis of NOX-6-18

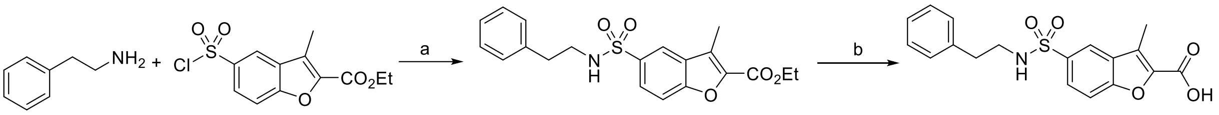

NOX-6-18

Scheme S2. Synthesis of **NOX-6-18**. a) DIPEA, DMF, rt, 77% b) LiOH, THF, MeOH, H_2O, 60°C, 66%.

Ethyl 3-methyl-5-(*N*-phenethylsulfamoyl)benzofuran-2-carboxylate

2-Phenylethylamine (0.363 mmol, 46.2 µL) was added to a solution of ethyl 5-(chlorosulfonyl)-3-methyl-1-benzofuran-2-carboxylate (0.330 mmol, 100 mg) in DMF (3.0 mL) at rt under Ar. DIPEA (0.660 mmol, 113 µL) was then added and the resulting solution was stirred at rt for 3h. Water was added and the aqueous phase was extracted with ethyl acetate (3x). The combined organic layers were dried over magnesium sulfate, filtered and evaporated. The residue was purified by flash chromatography (cyclohexane/ethyl acetate, 9:1 to 6:4) to yield 98.8 mg (77%) of pure compound. ^1H NMR (400 MHz, $CDCl_3$) δ 8.15 (d, *J* = 1.2 Hz, 1H), 7.84 (dd, *J* = 8.7, 1.7 Hz, 1H), 7.61 (d, *J* = 8.8 Hz, 1H), 7.24-7.17 (m, 3H), 7.05 (d, *J* = 6.9 Hz, 1H), 4.48 (q, *J* = 7.1 Hz, 2H), 4.44 (d, *J* = 5.0 Hz, 1H), 3.26 (q, *J* = 6.5 Hz, 2H), 2.77 (t, *J* = 6.8 Hz, 2H), 2.60 (s, 3H), 1.46 (t, *J* = 7.2 Hz, 3H).

3-Methyl-5-(*N*-phenethylsulfamoyl)benzofuran-2-carboxylic acid (NOX-6-18)

Ethyl 3-methyl-5-(N-phenethylsulfamoyl)benzofuran-2-carboxylate (0.250 mmol, 96.9 mg) was dissolved in THF (5.0 mL). Lithium hydroxide (2.50 mmol, 61.1 mg) dissolved in a few µL of water was added. Methanol was added until a monophasic solution was obtained and the reaction was heated at 60 °C overnight. After this time, aqueous 1M HCl solution was added and the aqueous

phase was extracted with ethyl acetate (3x). The combined organic layers were dried over magnesium sulfate, filtered and evaporated. The residue was purified by preparative HPLC to yield 59.4 mg (66%) of pure compound. ^{1}H NMR (500 MHz, DMSO-*d*$_6$) δ 8.17 (d, *J* = 1.5 Hz, 1H), 7.87 (dd, *J* = 8.8, 1.5 Hz, 1H), 7.82 (d, *J* = 8.8 Hz, 1H), 7.74 (t, *J* = 5.8 Hz, 1H), 7.24-7.21 (m, 2H), 7.16-7.12 (m, 3H), 2.98 (q, *J* = 6.1 Hz, 2H), 2.67 (t, *J* = 7.3 Hz, 2H), 2.56 (s, 3H); ^{13}C NMR (125 MHz, DMSO-*d*$_6$) δ 160.9, 154.8, 144.0, 138.7, 135.7, 129.0, 128.6, 128.2, 126.2, 125.6, 123.8, 120.8, 112.8, 44.0, 35.2, 9.0; tR HPLC: 11.8 Min (13 Min from 10 to 95% MeCN in water (0.1 % formic acid), then 7 min 95% MeCN). 99.9% purity; HRMS (MALDI): m/z found. 358.0757 $[M+H]^+$ (cal. $C_{18}H_{18}N_2O_5S^+$ 358.0755).

HPLC traces. Conditions**:** Luna 10 µm C18(2) 100 Å, LC Column 250 x 4.6 mm from Phenomenex. Acetonitrile and aqueous formic acid 0.1% were used as eluents. Gradient from 90% to 5% water for 13 min then 7 min 5% water.

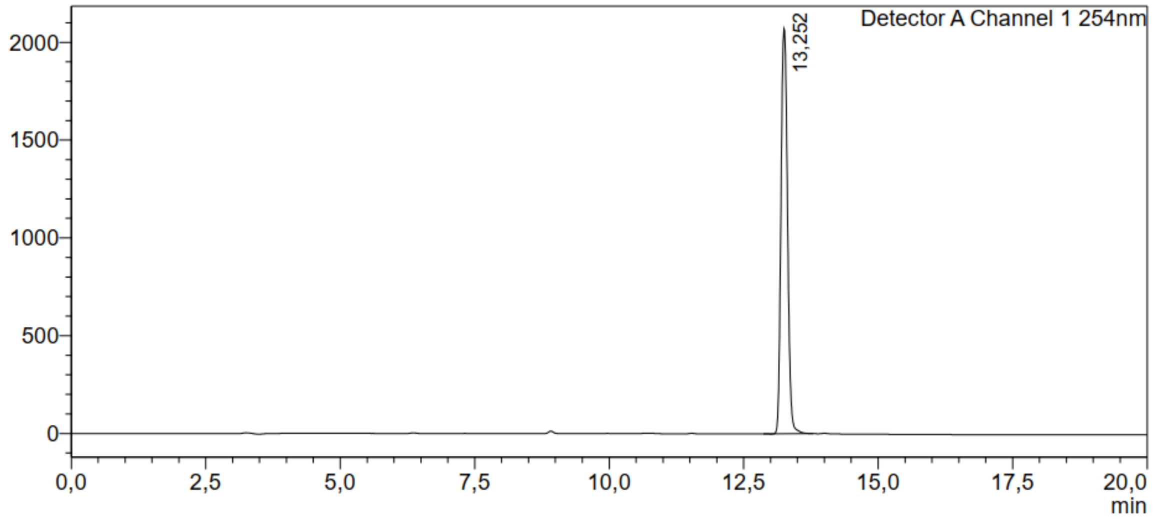

Detector A Channel 1 254nm

Peak#	Ret. Time	Area	Height	Conc.
1	13,252	17449276	2070653	100,000
Total		17449276	2070653	

SI Figure S1: HPLC trace compound **31**.

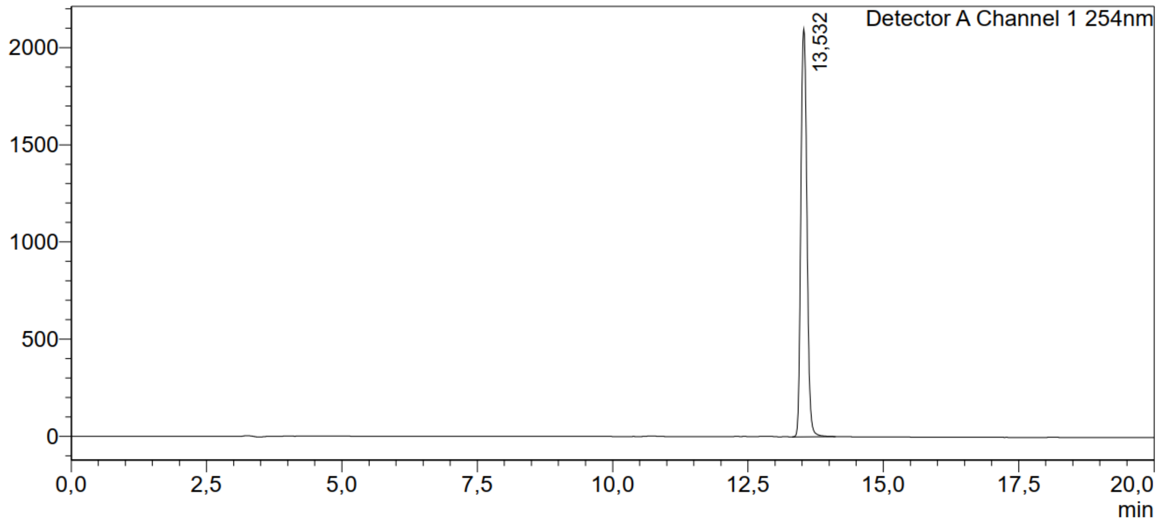

Detector A Channel 1 254nm

Peak#	Ret. Time	Area	Height	Conc.
1	13,532	16479348	2097383	100,000
Total		16479348	2097383	

SI Figure S2: HPLC trace compound **32**.

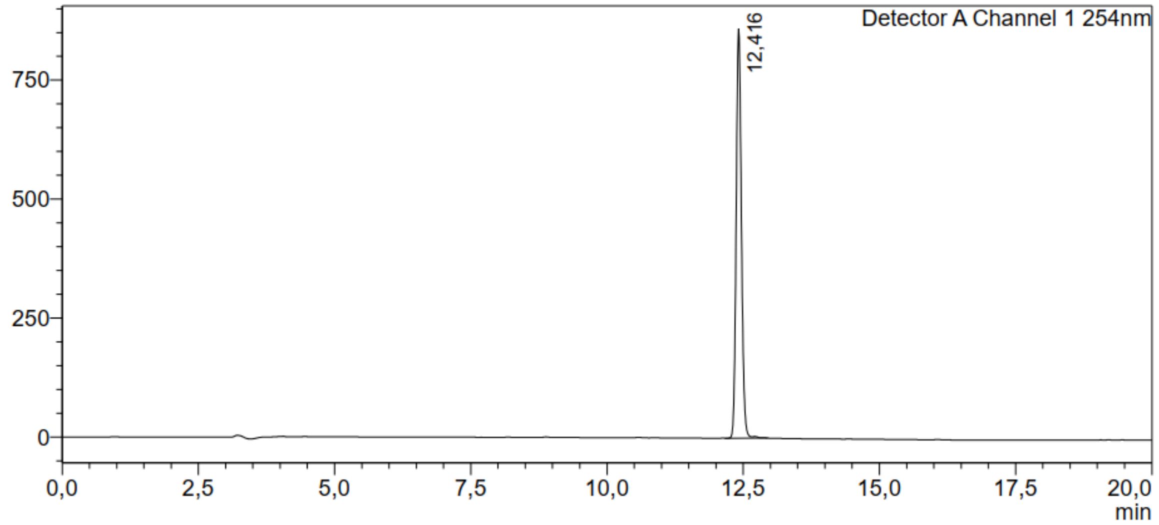

Detector A Channel 1 254nm				
Peak#	Ret. Time	Area	Height	Conc.
1	12,416	5783756	859991	100,000
Total		5783756	859991	

SI Figure S3: HPLC trace compound **34**.

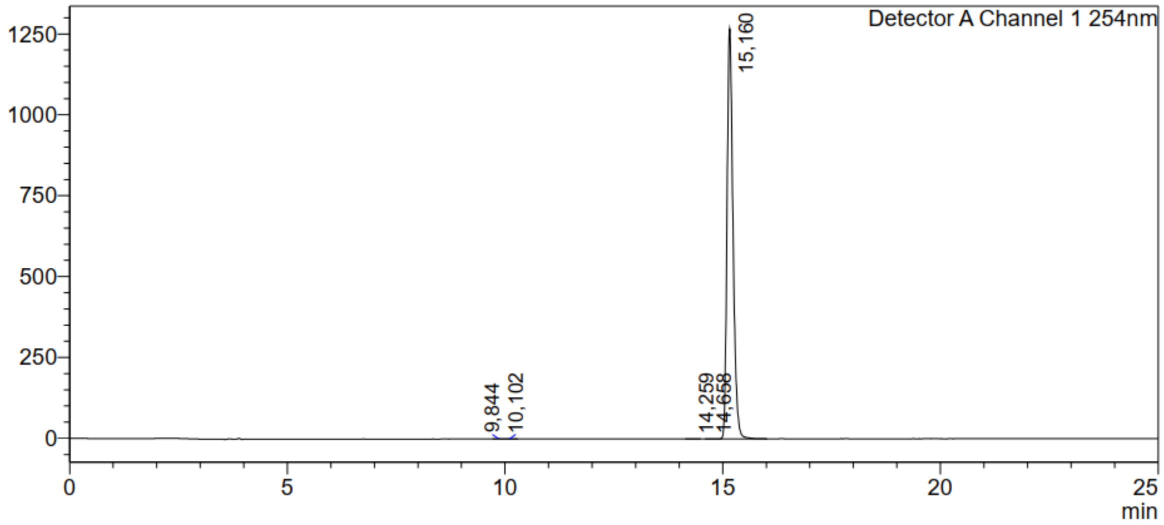

Detector A Channel 1 254nm				
Peak#	Ret. Time	Area	Height	Conc.
1	9,844	7352	1216	0,061
2	10,102	3585	586	0,030
3	14,259	2697	303	0,022
4	14,658	1044	111	0,009
5	15,160	12050336	1266866	99,878
Total		12065013	1269082	

SI Figure S4: HPLC trace compound **65**.

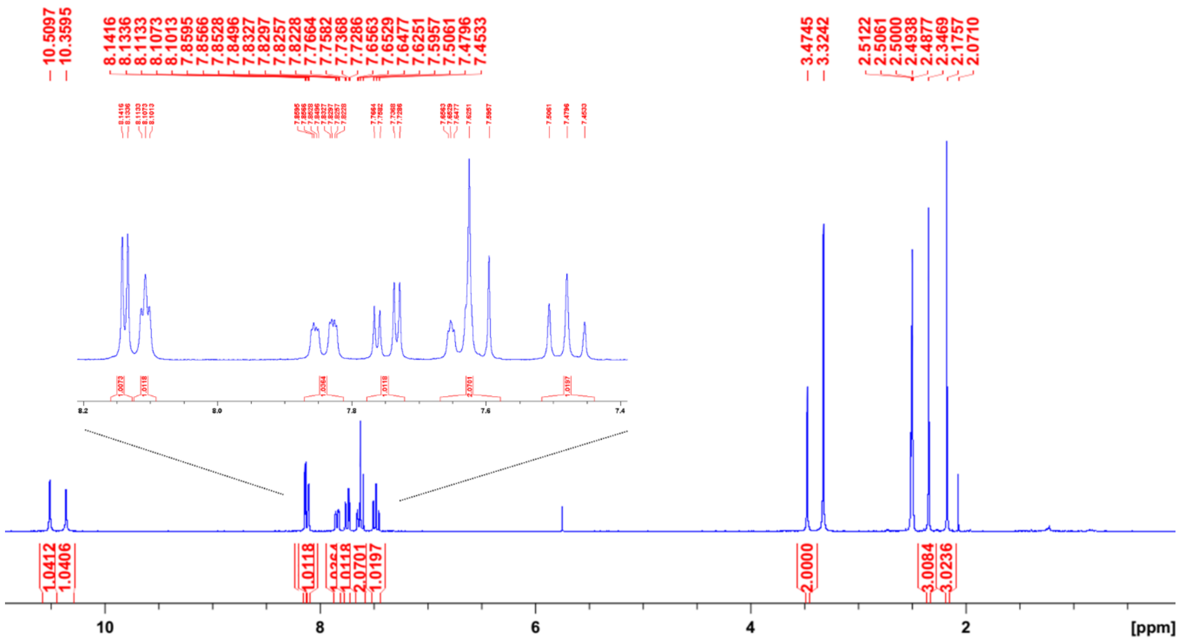

SI Figure S5: ^{1}H NMR (300 MHz, DMSO-d_6) of compound **31**.

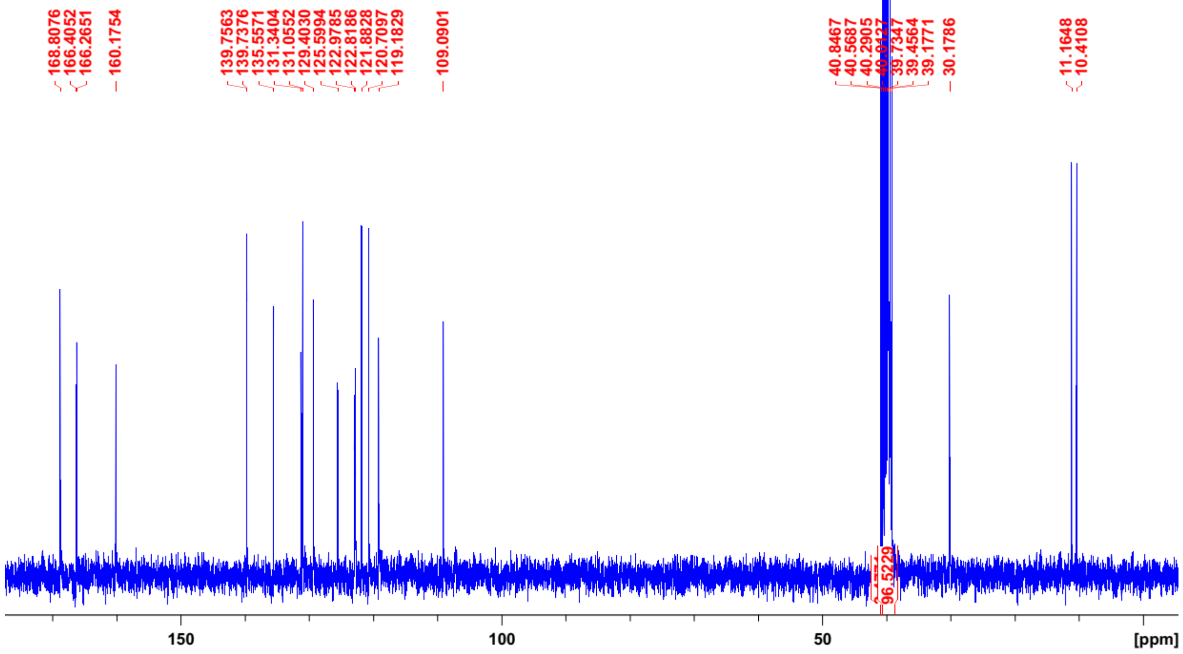

SI Figure S6: ^{13}C NMR (75 MHz, DMSO-d_6) of compound **31**.

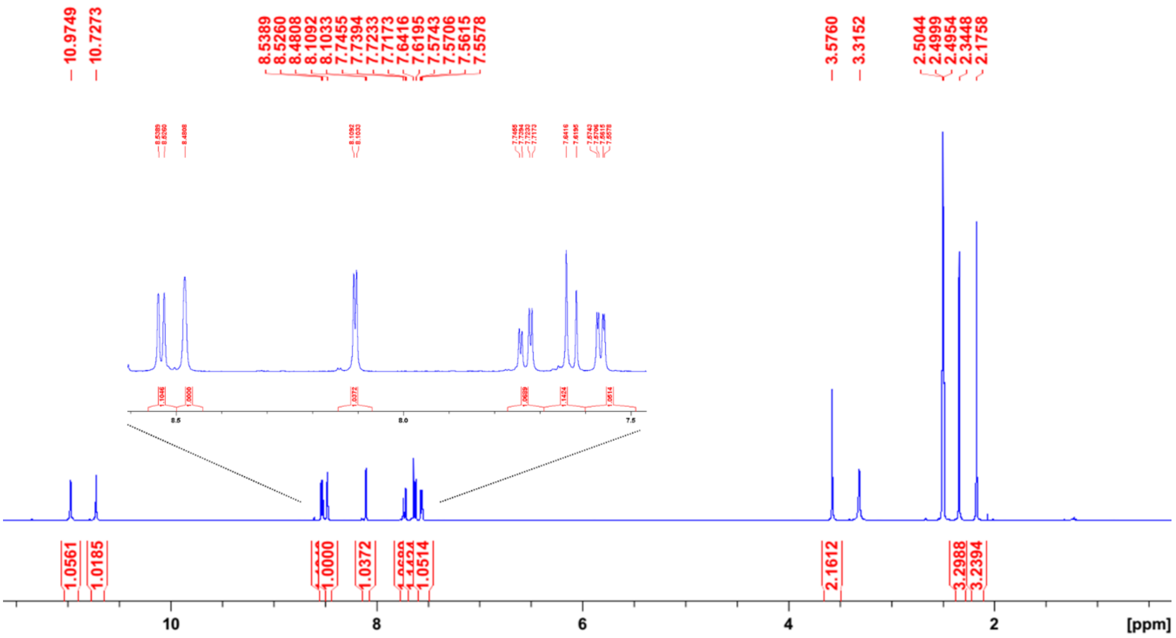

SI Figure S7: ^{1}H NMR (400 MHz, DMSO-d_6) of compound **65**.

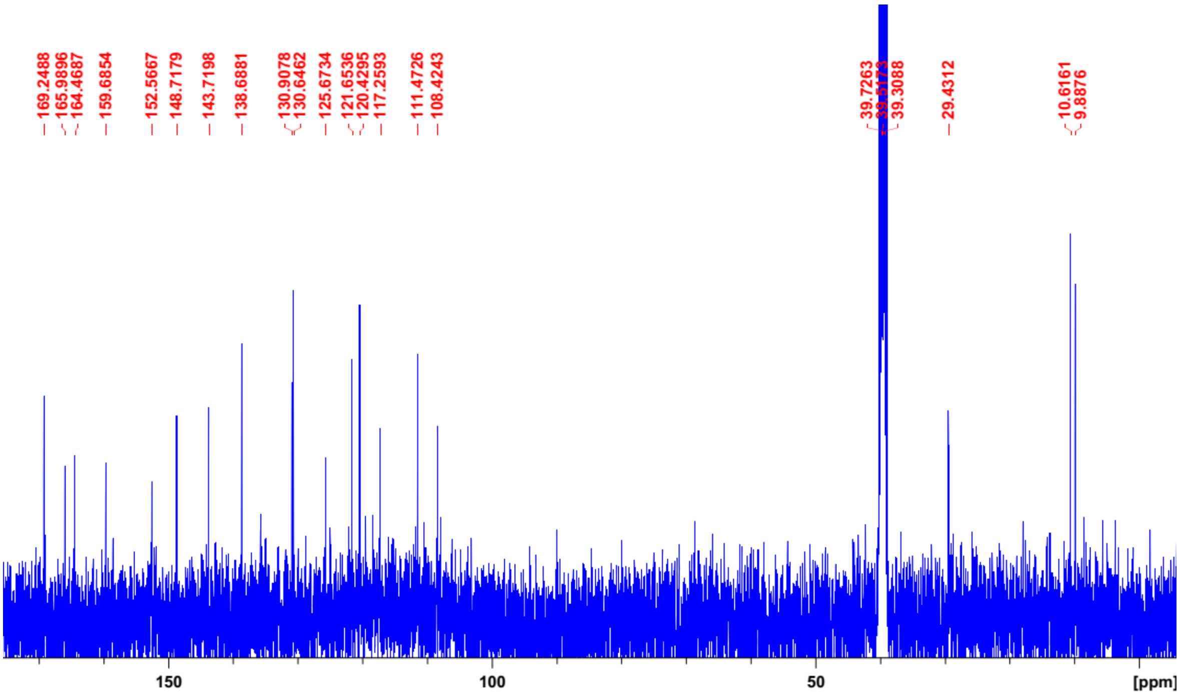

SI Figure S8: ^{13}C NMR (100 MHz, DMSO-d_6) of compound **65**.

Materials and methods for the mouse PK experiment with compound 65 (FL319)

Reagents and consumables

DMSO Chromasolv Plus, HPLC grade, ≥99.7% (Sigma-Aldrich, USA; Cat #34869);

Acetonitrile Chromasolv, gradient grade, for HPLC, ≥99.9% (Sigma-Aldrich, USA; Cat #34851);

Methanol Chromasolv Plus, for HPLC, ≥99.9% (Sigma-Aldrich, USA; Cat #34860);

Formic acid for mass spectrometry, ~98% (Fluka, USA; Cat #94318);

DMSO (USP, BP, Ph. Eur.), Pharma grade (PanReac Applichem, Germany; 191954.1611);

Kolliphor HS 15 (USP, Ph. Eur.), pharma grade (BASF SE, Germany; 50379938);

Water for injections (WFI) ("Yuria-Pharm", Ukraine; Lot #BG312/1-1);

2,2,2-Tribromoethanol 97% (Sigma-Aldrich; Cat # T48402);

Amyl alcohol (UOS, Ukraine);

Tubes (Falcon, 5 ml, 12 x 75 mm, USA);

Microtainer Blood Collection Tubes K_3EDTA, Henso, Lot #191010

Syringes (BD, 1 ml, tuberculin slip tip, USA, REF 309659).

Compound Mandipropamid was used as an internal standard (IS).

Compound **65** was supplied as a dry powder. DMSO – Kolliphor HS 15 – Water for injections

(10%:20%:70%, v/v/v) was used as a formulation vehicle.

To prepare the formulation, 0.490 ml of DMSO was added to the compound (3 mg, 98% purity); the mixture was vortexed for 10 sec and sonicated for 2 min at 45ºC – compound dissolved. Next, 0.980 ml of Kolliphor HS (pre-warmed to 45ºC) was added to the formulation; the mixture was vortexed for 10 sec and To prepare the formulation, 0.490 ml of DMSO was added to the compound (3 mg, 98% purity); the mixture was vortexed for 10 sec and sonicated for 2 min at 45ºC – compound dissolved. Next, 0.980 ml of Kolliphor HS (pre-warmed to 45ºC) was added to the formulation; the mixture was vortexed for 10 sec and sonicated for 1 min at 45ºC – clear solution. After that, 3 ml of water was added to the formulation; the mixture was vortexed for 10 sec and sonicated for 1 min at 45ºC – clear colorless solution (pH 7.31). Then, 430 uL of water was added to the formulation; the mixture was vortexed for 10 sec – clear colorless solution. The batches of working formulations were prepared 5 min prior to the in vivo study.

Equipment

Gradient HPLC system (Shimadzu, Japan);

MS/MS detector API 3000 with TurboIonSpray Electrospray module (AB Sciex, Canada);

IMT-PN 1280 OG Nitrogen Generator (INMATEC Technologies GmbH, Germany);

Water purification system Arium mini (Sartorius, Germany);

VWR Analog Vortex Mixer VM 3000 (VWR, USA);

Centrifuge 4-15C (Qiagen) (Sigma, Germany).

Study design

Study design, animal selection, handling and treatment were all in accordance with the Enamine PK study protocols and Institutional Animal Care and Use Guidelines (BACUC approval number #GUF-PK-26022024). Animal treatment and samples preparation were conducted by the Animal Laboratory personnel at Enamine/Bienta. Male CD-1 mice (8 weeks old, body weight ranged from 24.1 g to 33.7 g and average body weight across all groups 29.4 g, standard deviation (SD) = 2.8 g) were used in this study. The animals were randomly assigned to the treatment groups before the pharmacokinetic study. Intraperitoneal (IP) route of administration and six sampling time points (5, 15, 30, 60, 240, and 480 min) were set for this pharmacokinetic study. Each of the time point treatment groups included 3 animals. There was also a control group of one animal per route. Dosing was done according to the treatment schedule outlined in **Table S1**. Mice were injected IP with 2,2,2-tribromoethanol at the dose of 150 mg/kg prior to drawing the blood. Blood collection was performed from the orbital sinus in microtainers containing K_3EDTA. Animals were sacrificed by cervical dislocation after the blood samples collection. Blood samples were centrifuged for 10 min at 3000 rpm. All samples were immediately processed, flash-frozen at dry ice, and stored at -70°C until subsequent analysis.

SI Table S1. Study design

Number of animals	Compound ID	Formulation	Delivery Route	Target Dose Level (mg/kg)	Target Dose Concentration (mg/mL)	Target Dose Volume (mL/Kg)
18	**65**	DMSO – Kolliphor HS 15 – Water for injections (10%:20%:70%)	IP	3	0.6	5
1	Vehicle dosed		IP	0	0	5

Samples processing

Plasma samples (40 µl) were mixed with 200 µl of IS(90) solution. After mixing by pipetting and centrifuging for 4 min at 6,000 rpm, 1 µl of each supernatant was injected into LC-MS/MS system. The solution of compound Mandipropamid (500 ng/ml in water-methanol mixture 1:9, v/v) was used as an internal standard (IS(90)) for the quantification of **65** in plasma samples.

Samples analysis

Analyses of plasma samples were conducted by the Bioanalytical Laboratory personnel at Enamine/Bienta. The concentrations of **65** in samples were determined using high performance liquid chromatography/tandem mass spectrometry (HPLC-MS/MS) method. Shimadzu HPLC system comprised 2 isocratic pumps LC-20AD, an autosampler SIL-20AC, a sub-controller FCV-14AH and a degasser DGU-14A. Mass spectrometric analysis was performed using an API 3000 (triple-quadrupole) instrument from AB Sciex (Canada) with an electro-spray (ESI) interface. The data acquisition and system control were performed using Analyst 1.6.3 software from AB Sciex.

HPLC-MS/MS Conditions

Chromatographic Conditions:

Column: Synergi 4u Hydro-RP 80A, 2.0 x 30 mm, 4 µm

Mobile phase A: Acetonitrile : Water : Formic acid = 50 : 950 : 1

Mobile phase B: Acetonitrile : Formic acid = 100 : 0.1

Linear gradient: 0 min 25% B, 0.9 min 100% B, 1.2 min 100% B, 1.21 min 25% B, 2.5 min stop

Elution rate: 400 µL/min. A divert valve directed the flow to the detector from 1.4 to 1.8 min

Column temperature: 30°C

<u>MS/MS Detection:</u>

Scan type: Positive MRM, Ion source: Turbo spray, Ionization mode: ESI

Nebulize gas: 15 L/min, Curtain gas: 8 L/min, Collision gas: 4 L/min

Ionspray voltage: 5000 V, Temperature: 400°C

SI Table S2. Other MS Parameters

Compound ID	Parent, m/z	Daughter, m/z	Time, ms	DP, V	FP, V	EP, V	CE, V	CXP, V
**65**	419.019	282.100	80	66	320	11	35	18
Mandipropamid	412.200	328.200	80	46	250	11	21	20

<u>Preparation of calibration standards</u>

Calibration standards for quantification of **65** in plasma samples. Compound **65** was dissolved in DMSO, and the resulting solution with a concentration of 2 mg/ml was used for calibration standards preparation (stock solution). The stock solution was consecutively diluted with IS(90) to get a series of calibration solutions with final concentrations of 10 000, 4 000, 2 000, 1 000, 400, 200, 100, 40, 20, 10, 4, 2, and 1 ng/ml. The calibration curve was constructed using blank mouse plasma samples. To obtain calibration standards, blank plasma samples (40 µl) were mixed with 200 µl of the corresponding calibration solution. After mixing by pipetting and centrifuging for 4 min at 6000 rpm, 1 µl of each supernatant was injected into LC-MS/MS system.

<u>Method validation results</u>

Specificity: **Figure S9** shows that the blank mouse plasma had no interference with compound **65** (FL319) and IS.

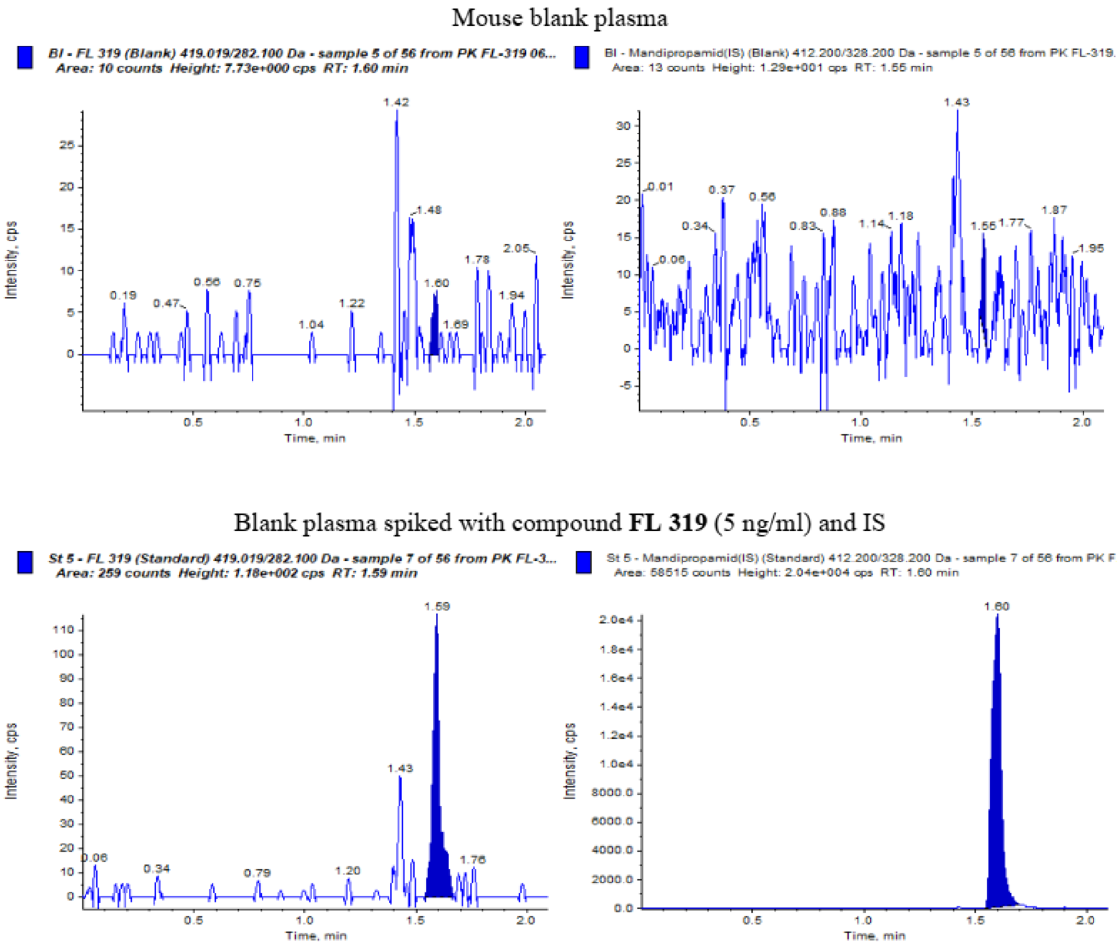

SI Figure S9: Method validations results

<u>Calibration curve</u>

The regression analysis of **65** (FL319) was performed by plotting the peak area ratio (y) against the compound concentration in calibration standards (x, ng/ml). The validity of the calibration curve (relationship between peak area ratio and compound concentration) is proved by the correlation coefficient (R) calculated for the linear regression (**Figure S10**).

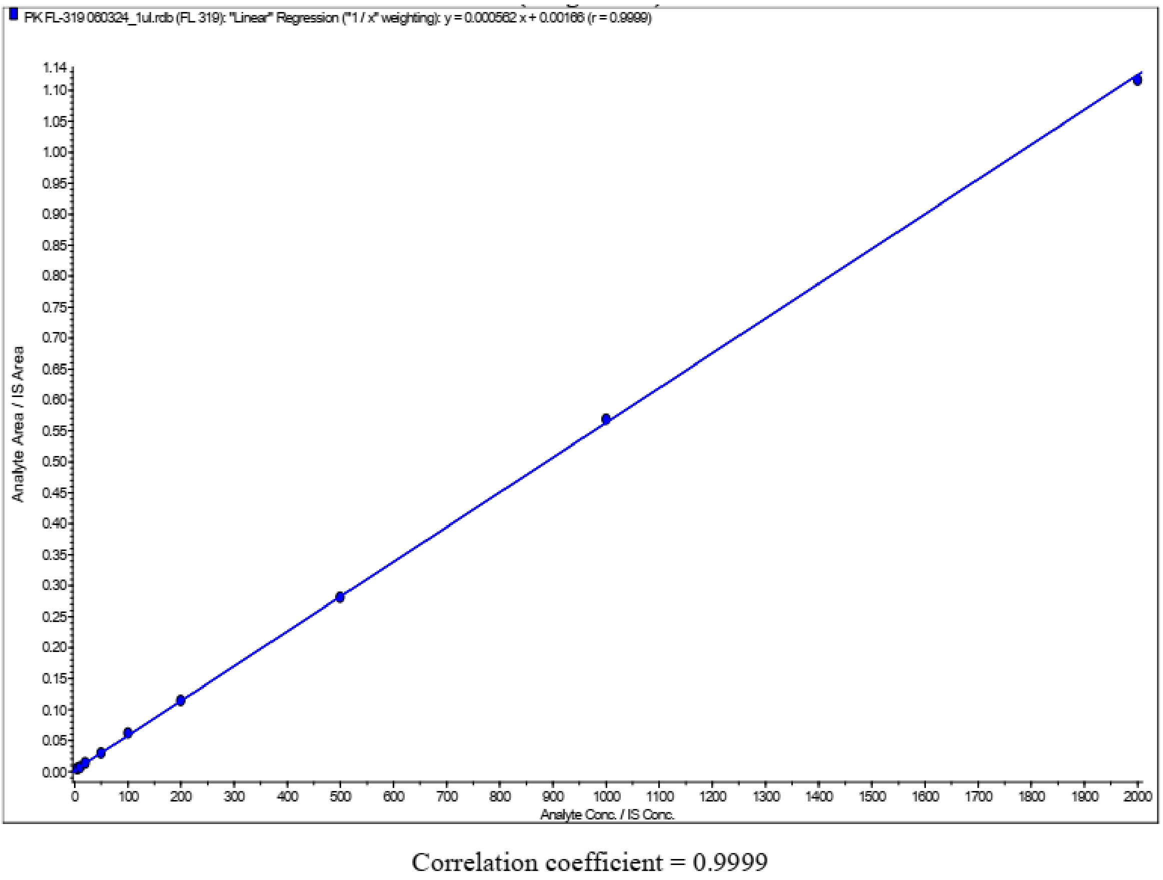

SI Figure S10: Calibration curve for compound **65** (FL319).

<u>Pharmacokinetic method analysis</u>

The concentrations of the test compound below the lower limit of quantitation (LLOQ = 5 ng/ml) were designated as zero. The pharmacokinetic data analysis was performed using noncompartmental, bolus injection or extravascular input analysis models in WinNonlin 5.2 (PharSight). Data below LLOQ were presented as missing to improve the validity of T½ calculations.

For each treatment condition, the final concentration values obtained at each time point were analyzed for outliers using Grubbs' test with the level of significance set at $p < 0.05$.

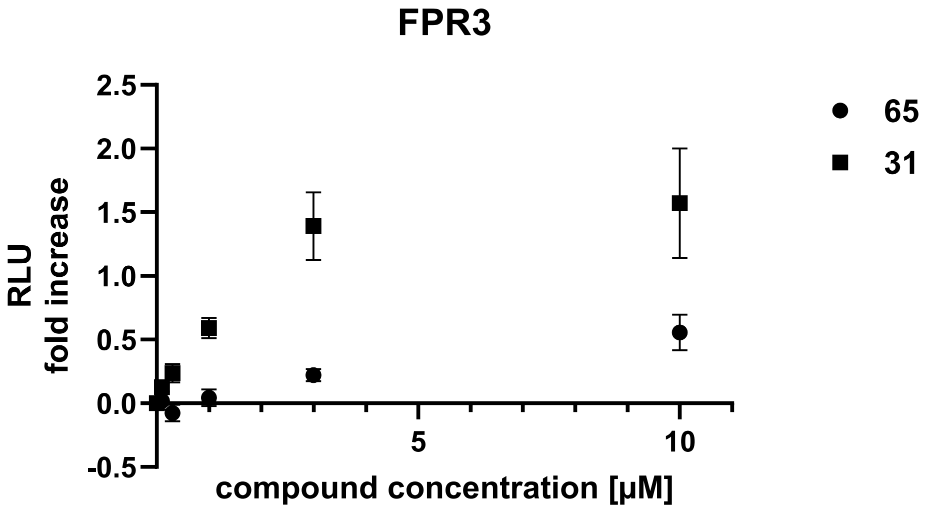

SI Figure S11: Compounds **65 and 31** dose-dependently activate FPR3 in the PRESTO-Tango β-arrestin recruitment assay. The data is normalized to DMSO baseline and the results are presented as mean ± SEM (n = 3, with technical replicates N = 4 for each).

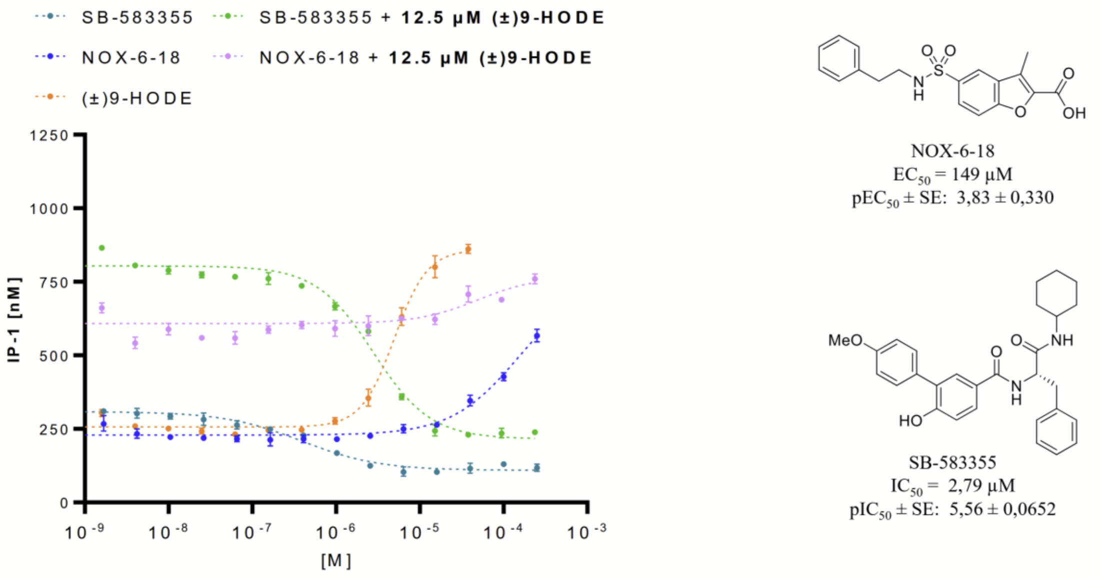

SI Figure S12: Dose-dependent response of reference compounds **NOX-6-18 and SB-583355** in CHO-K1 cell line expressing G2A and for GNA11 in IP-One assay, measured in the agonist and the antagonist (in presence of 12.5 µM (±) 9-HODE) mode.
